# Genome sequences of *Tropheus moorii* and *Petrochromis trewavasae*, two eco-morphologically divergent cichlid fishes endemic to Lake Tanganyika

**DOI:** 10.1038/s41598-021-81030-z

**Published:** 2021-02-22

**Authors:** C. Fischer, S. Koblmüller, C. Börger, G. Michelitsch, S. Trajanoski, C. Schlötterer, C. Guelly, G. G. Thallinger, C. Sturmbauer

**Affiliations:** 1 Institute of Biology, University of Graz, Graz, Austria; 2 Institute of Biomedical Informatics, Graz University of Technology, Graz, Austria; 3 Center for Medical Research, Medical University of Graz, Graz, Austria; 4 Institut für Populationsgenetik, Vetmeduni Vienna, Vienna, Austria; 5 BioTechMed-Graz, Graz, Austria

**Keywords:** Computational biology and bioinformatics, Ecology, Evolution, Genetics

## Abstract

With more than 1000 species, East African cichlid fishes represent the fastest and most species-rich vertebrate radiation known, providing an ideal model to tackle molecular mechanisms underlying recurrent adaptive diversification. We add high-quality genome reconstructions for two phylogenetic key species of a lineage that diverged about ~ 3–9 million years ago (mya), representing the earliest split of the so-called modern haplochromines that seeded additional radiations such as those in Lake Malawi and Victoria. Along with the annotated genomes we analysed discriminating genomic features of the study species, each representing an extreme trophic morphology, one being an algae browser and the other an algae grazer. The genomes of *Tropheus moorii* (TM) and *Petrochromis trewavasae* (PT) comprise 911 and 918 Mbp with 40,300 and 39,600 predicted genes, respectively. Our DNA sequence data are based on 5 and 6 individuals of TM and PT, and the transcriptomic sequences of one individual per species and sex, respectively. Concerning variation, on average we observed 1 variant per 220 bp (interspecific), and 1 variant per 2540 bp (PT vs PT)/1561 bp (TM vs TM) (intraspecific). GO enrichment analysis of gene regions affected by variants revealed several candidates which may influence phenotype modifications related to facial and jaw morphology, such as genes belonging to the Hedgehog pathway (*SHH*, *SMO*, *WNT9A*) and the BMP and GLI families.

## Introduction

With 1727 described species^1^, cichlid fishes are among the most species-rich teleost fish families. Their hotspot of biodiversity lies in East Africa, and in particular the three Great Lakes, Victoria, Malawi and Tanganyika^2^. Despite a large degree of similarity pointing to recurrent evolution of eco-morphologically equivalent species^3^, the three cichlid radiations show important differences with respect to species numbers, evolutionary age of lineages, diversity of parental care patterns and the degree of morphological divergence^2–4^. This is likely due to different sets of colonizing species and most importantly due to their different evolutionary age.

With an age of 9–12 million years (myr)^5,6^, Lake Tanganyika is by far the oldest of these lakes. Due to its old age, the Lake Tanganyika species assemblage is at a mature stage, so that it comprises the largest genetic and phenotypic diversity among the East African cichlid radiations, but further diversification proceeds predominately without much eco-morphological innovation^2^. Upon colonization of the emerging lake, the cichlids took advantage of the window of ecological opportunity and rapidly diversified^4^. In fact, two colonizing lineages underwent hybridization at the very onset of the radiation, an event that might have triggered or boosted the start^6^. The Lake Tanganyika radiation holds a key position for the entire modern African cichlid fauna, in that three of the newly emerging lacustrine lineages managed to colonize surrounding rivers, so that the radiation repeatedly swept over the boundaries of the maturing lake^7–10^. Three of the emerging lineages, the non-mouthbrooding Lamprologini, the mouthbrooding Orthochromini and some early Haplochromini such as the ancestors of the genera *Pseudocrenilabrus* and *Serranochromis*, left the lake at various stages of lake maturation to colonize particular surrounding water bodies^7–9,11–13^. One group of early haplochromines continued to evolve in the lake-swamp-river interface towards more elaborate maternal mouthbrooders, demarcated by increased sexual dimorphism and eggspots on the anal fin^6,9^, the so-called modern haplochromines. These modern haplochromines not only colonized most river systems all over southern and eastern Africa but re-entered the—at this time already much deeper and mature—Lake Tanganyika ecosystem, to evolve into the endemic Lake Tanganyika tribe Tropheini^9,14^. Thus, the Tropheini managed to break into an ongoing and already complex lacustrine radiation, while its non-lacustrine sisters spread across several river systems to seed radiations in emerging lakes along their routes of riverine dispersal^6,8,9,15,16^.

The Lake Tanganyika-endemic tribe Tropheini represents the sister group of all modern haplochromines outside the lake and diverged from these ~ 3–9 mya^6^. That five out of the 29 species of the Tropheini both occur in the lake itself and upstream in tributary rivers and/or parts of the Lukuga River, the lake’s only outflow, might be owed to their swamp-river origin^17^. This is why we decided to sequence and compare the genomes of two ecologically divergent species of the endemic Lake Tanganyika tribe Tropheini. In terms of genetics, the modern haplochromines, including the Tropheini, are iconic as their generalist riverine-adapted genomes repeatedly underwent recurrent adaptive modifications upon ecological opportunity—provided by newly emerging lakes^4^. It has been suggested that ecologically and phenotypically flexible species adapted to seasonally unstable river habitats can outcompete other colonizers in seeding lacustrine radiations, as they can rapidly accommodate empty niche space via phenotypic plasticity^18^. According to the flexible stem hypothesis, a phenotypically plastic population is subdivided into alternative adaptive phenotypes and subsequently adaptive genetic factors are sorted during speciation to proceed further via genetic accommodation and genetic assimilation. In the course of adaptive divergence during repeated adaptive radiations, genomic evolution was likely shaped by ecological opportunity, in combination with geographic fragmentation events, episodes of bottlenecks and population expansions, as well as repeated admixes or fusions in hybridization events caused by climate-induced lake level fluctuations^4,19^. Along with divergence and incidental gene flow^6,20^, gene duplication and selection^6,21^ events apparently reshaped the genotypes. On the phenotype level, the evolutionary success of East African cichlids has been attributed to particular key innovations including (1) the functional decoupling of oral and pharyngeal jaws facilitating the exploitation of diverse trophic niches^22^, (2) the adaptation of the visual system to different water turbidity^23^, and (3) parental care and male mating coloration driven by sexual selection facilitating reproductive isolation^24^. At this stage, the suite of genetic mechanisms modifying the genomic substrate underlying the enormous phenotypic eco-morphospace covered by cichlids remains largely unknown (see^25^ for a recent review).

The first major steps towards understanding the molecular mechanisms behind those divergent morphologies were taken by elucidating the genomes and transcriptomes of five cichlid species: *Oreochromis niloticus* representing an outgroup lineage, *Neolamprologus pulcher* representing a Tanganyikan substrate brooder lineage, and three modern haplochromines, namely *Astatotilapia burtoni* representing a riverine lineage, *Maylandia zebra* representing Lake Malawi and *Pundamilia nyererei* representing Lake Victoria. This study found evidence for an excess of gene duplications in the East African lineage compared to *Oreochromis* and other teleosts, an abundance of non-coding element divergence, accelerated coding sequence evolution, expression divergence along with transposable element insertions, and regulation by novel microRNAs^21^. The study also revealed genome-wide diversifying selection on coding and regulatory variants, some of which recruited from ancient polymorphisms.

**Figure 1:**
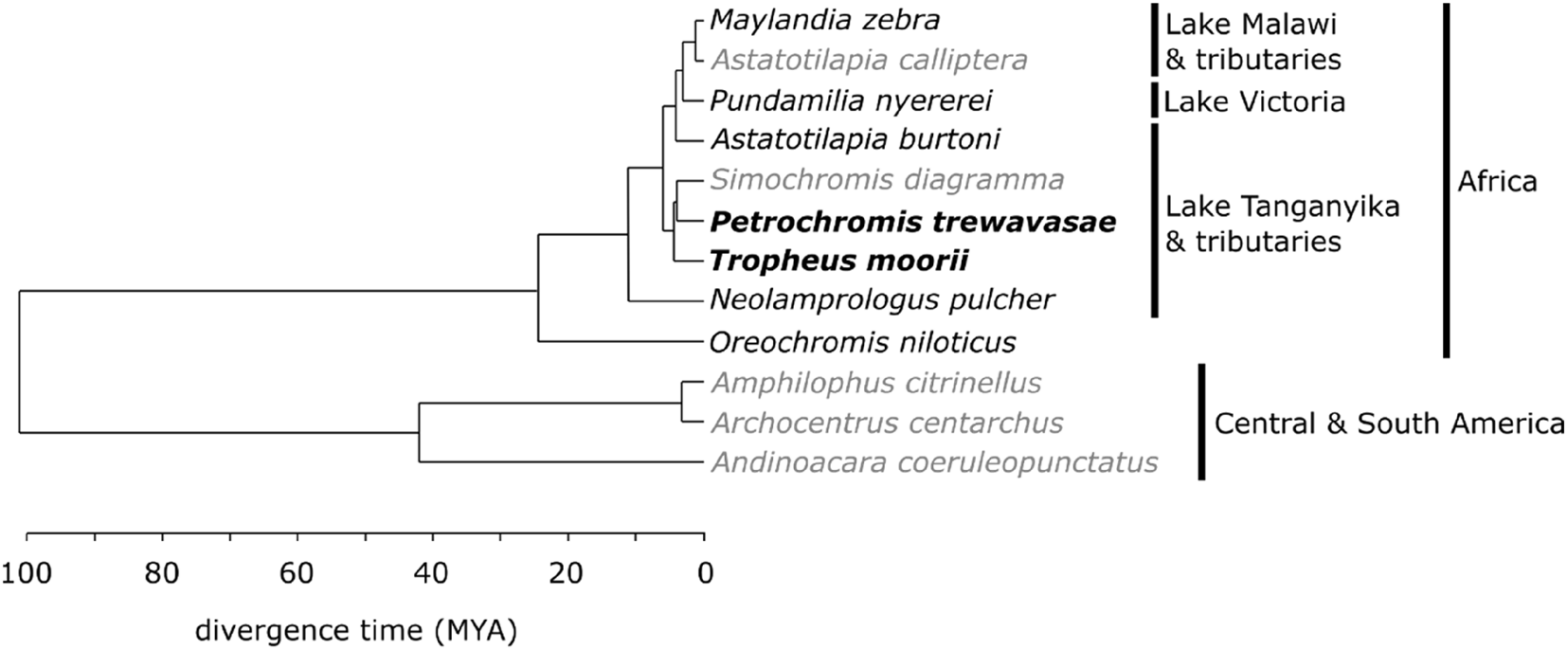
Time calibrated phylogeny of cichlid species with publicly available complete genomes. The phylogeny is based on 519 anchored genome loci either sequenced or extracted from the genome sequence^6^. The study species are highlighted in bold, species with annotated high quality genomes are in black and species with high quality assemblies (but not yet annotated genomes) are indicated in grey (Figure created with Inkscape https://inkscape.org).

High quality (HQ) genome drafts based on Pacific Biosciences (PacBio) data became available especially in the last two years. HQ drafts of *Simochromis diagramma* (East Africa, Lake Tanganyika) and *Astatotilapia calliptera* (East Africa, Lake Malawi) were generated by the Sanger Institute (2018) and a HQ draft of *Archocentrus centrarchus* (Central America) was generated by the G10K-VGP group (2019); assemblies of the South American cichlids *Amphilophus citrinellus* (2014, University of Konstanz) and *Andinoacara coeruleopunctatus* (2015, Sanger Institute)^26^ are also available. The *O. niloticus* (ON) and *M. zebra* (MZ) genomes have recently (2019) been newly assembled and anchored with a high-coverage PacBio + genetic map approach^27^; the genomes of *A. calliptera, A. centrarchus* and *S. diagramma* (not anchored) were reconstructed similarly. *Oreochromis niloticus, M. zebra*, *A. calliptera* and *A. centrarchus* are the only reconstructions on chromosome level (linkage groups). Seven HQ drafts received annotations from the NCBI Annotation Pipeline^28^ (*S. diagramma* not yet); *O. niloticus, A. calliptera* and *A. citrinellus* received annotations from Ensembl^29^ as well. These genomes cover species from the Great Lakes and rivers in Africa and from crater lakes in Central and South America (Fig. 1).

We present reconstructions of the two genomes of *Tropheus moorii* (TM) and *Petrochromis trewavasae* (PT), as well as sets of structural and functional annotations. The two species belong to two sublineages within the Tropheini that diverged ~ 2–6.5 mya^5^ and represent the deepest split within the tribe Tropheini. Aside from generating the genomic and transcriptomic data basis for two key species representing the modern haplochromines in Lake Tanganyika, the main interest in this study was in the genetic origin of the divergent facial and jaw morphologies of these morphologically diverse species. To this end we provide first insights on genetic variants potentially being involved in the morphological differentiation of the two study species.

## Results

### Assemblies

Based on the estimated genome sizes of ~ 900 Mbp (Supplementary Table S11), our sequencing efforts yielded sequence data with an average base coverage of ~ 1.5×, ~ 88× , ~ 34× and ~ 10.5× (PT) and ~ 1.2× , ~ 38× , ~ 29× and ~ 9.1× (TM) for Roche 454, Illumina PE, Illumina MP and PacBio, respectively (see Supplementary Table S23). The filtered sequence data was used to generate primary assemblies derived from different reconstruction algorithms (assemblers) and data combinations (see Methods). The final genome reconstructions of the two species are based on meta-assemblies of these sets of primary assemblies. The meta-assemblies with the best scores based on misassemblies, contiguity and gene predictions were used in subsequent analyses.

### *Petrochromis trewavasae*

The primary assemblies exhibit assembly sizes from ~ 779 Mbp to ~ 966 Mbp (907 Mbp PacBio only; see Supplementary Table S11). The final assembly consists of 7261 scaffolds with a N50 of 1.84 Mbp, 1.44% of nucleotides are undetermined (N) and 90% of the assembled genome is contained in 885 fragments longer than 70 kbp. The total assembly size is 917.57 Mbp (Table 1).

**Table 1:**
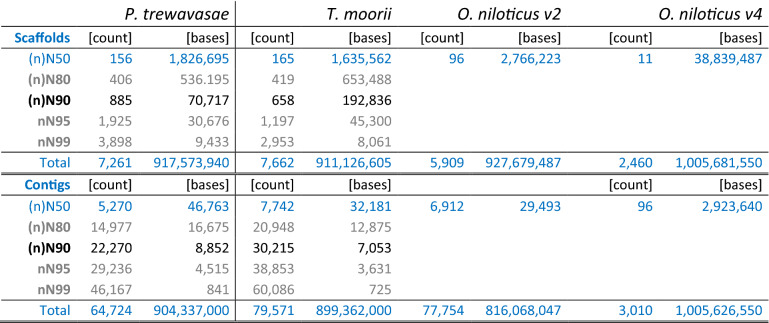
Assembly contiguity and size statistics: The assembled genomes consist of 917.57 and 911.13 Mbp for *P. trewavasae* and *T. moorii*, respectively. Count and number of bases for scaffolds and contigs are reported. Scaffolds were broken to contigs at stretches of Ns of length ≥ 10. Statistics on O. niloticus were obtained from NCBI and extended as necessary (in blue); technology-wise version 2 is comparable, version 4 is based on high-coverage PacBio and optical mapping data.

### *Tropheus moorii*

The primary assemblies exhibit assembly sizes from ~ 754 Mbp to ~ 952 Mbp (879 Mbp PacBio only; see Supplementary Table S11). The final assembly consists of 7662 scaffolds with a N50 of 1.64 Mbp, 1.29% of nucleotides are undetermined (N) and 90% of the assembled genome is contained in 657 fragments longer than 192 kbp. The total assembly size is 911.13 Mbp (Table 1). Both assembly sizes are in the expected range; k-mer spectra-based predictions hint to genome sizes of close to 900 Mbp (see Supplementary Table S11) and 900–1000 Mbp have also been reported for other cichlid genomes^21,30^.

In the following, we compare our results to published genomes and annotations of several cichlid fish with emphasis on *O. niloticus* and *M. zebra* due to their well-developed state. The latest versions (v4) of *O. niloticus* (44 × PacBio, newly anchored) and *M. zebra* (now 65 × PacBio and anchored) were published by Conte et al*.*^27^; the tendency with respect to earlier versions is clear, qualities of sequences and annotations are improved and the numbers of annotated structures were further increased. With respect to the gene length distributions (Supplementary Table S1), the contiguity measures achieved for PT and TM are satisfying and fall in the typical range, given the applied sequencing technologies and coverage (Table 1; for a comparison with *O. niloticus* versions see Supplementary Table S2, and for a general comparison with published fish genomes see Supplementary Table S23 of Vij et al.^31^).

### Annotations

Structural annotation yielded ~ 40,300 (PT) and 39,600 (TM) genes and ~ 54,200 (PT) and 56,800 (TM) transcripts, respectively (Table 2); this is in line with the results of different annotation versions of ON (~ 30,200 to 42,600 genes). As to annotated features, PT and TM show similar numbers which often lie between those of version 2 and 3 of the respective ON annotations. For comparison, statistics for ON v2–v4 (the latest) are added, as ON received the most community effort and data for genome assembly and annotation of all cichlids (Supplementary Table S2). Prediction of long non-coding RNAs yielded 2782 and 2112 lncRNAs for PT and TM, respectively. With 57.7% and 63.2% a slight preference for the sense strand could be observed (Supplementary Table S3). Homology based functional annotation could be made for 41,970 (PT) and 43,918 (TM) of the coding sequences (CDSs); putative secretory signals were predicted for 5899 (PT) and 6016 (TM) of them, respectively (Table 3). Pfam domain mapping yielded 78,900 (PT) and 84,158 (TM) hits, respectively. RepeatMasker^27^ identified 31.1% (PT) and 30.0% (TM) of the genomes as repetitive, respectively; the largest proportions of classified repeat types were held by DNA transposons, LINEs and LTR transposons with ~ 13%, ~ 7% and ~ 2% (Table 4).

**Table 2:**
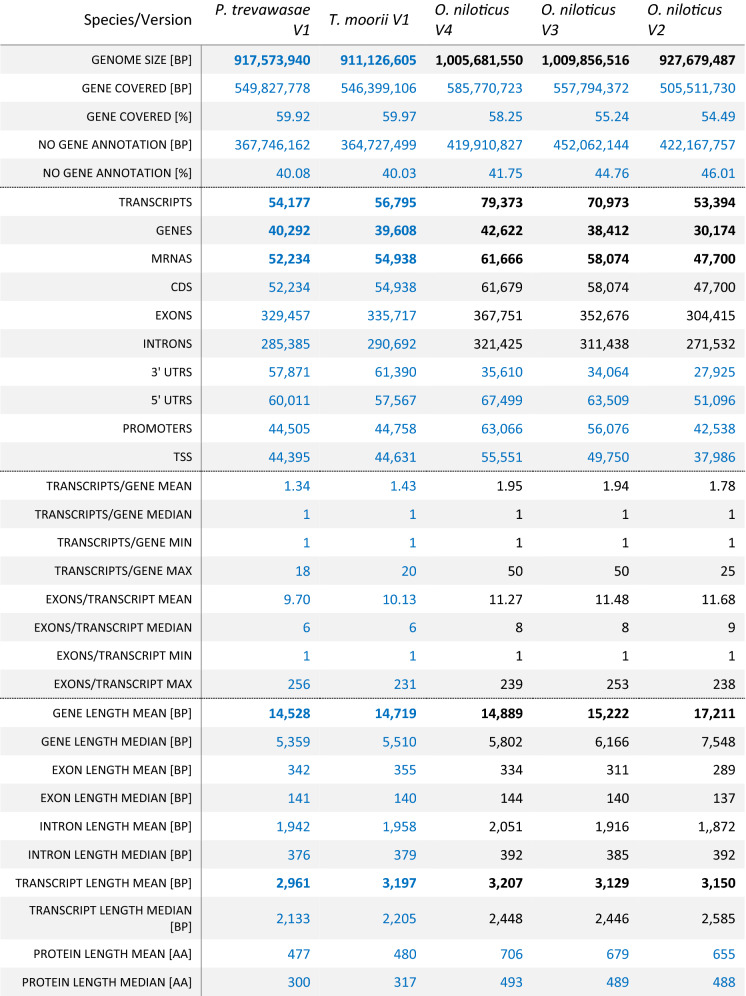
Structural annotation statistic of PT and TM in comparison with ON: Structural annotation yielded ~ 40,300 and 39,600 genes, respectively. This is in line with the results of different annotation versions of ON (~ 30,200 to 42,600).

**Table 3:**
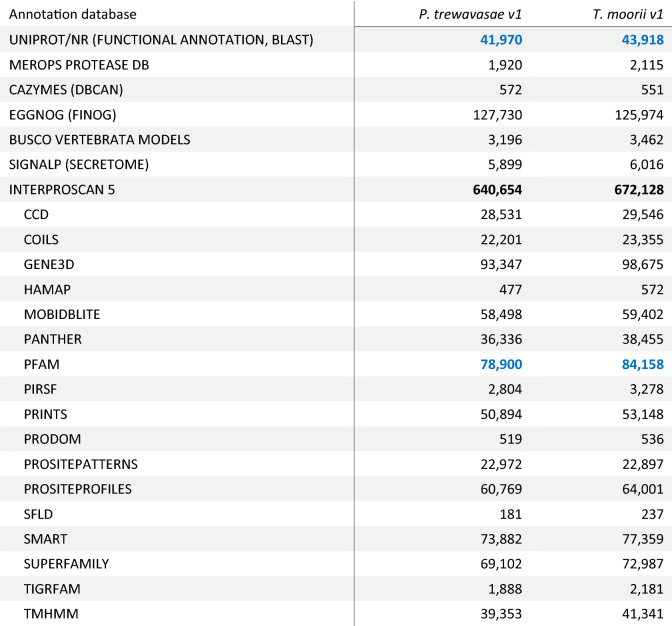
Functional annotation statistics: The number of proteins found in UniProt and NR are given. Furthermore, the table contains the number of proteins with putative protease (Merops) and carbohydrate activity (CAZymes), the number of orthologs in fiNOG, the number of proteins matching the BUSCO vertrebrate models and the number of proteins with putative secretory signals (SignalP). Finally, the number of hits of the protein sequences for the various InterPro domain databases are presented.

**Table 4:**
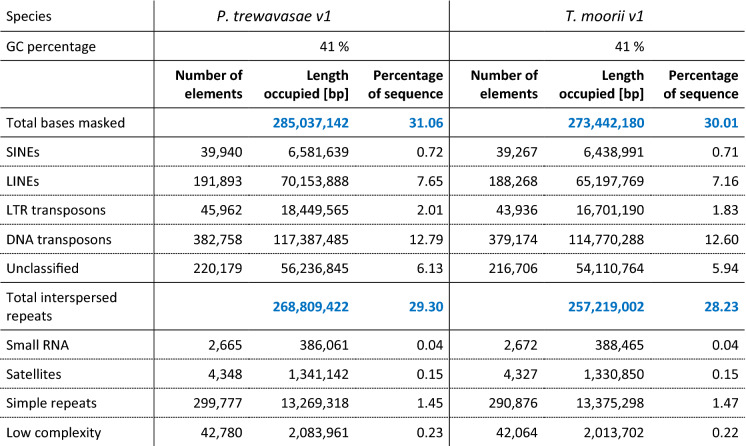
Repeat annotation statistics as determined by RepeatMasker^32^.

### Data availability and visualization

**Figure 2:**
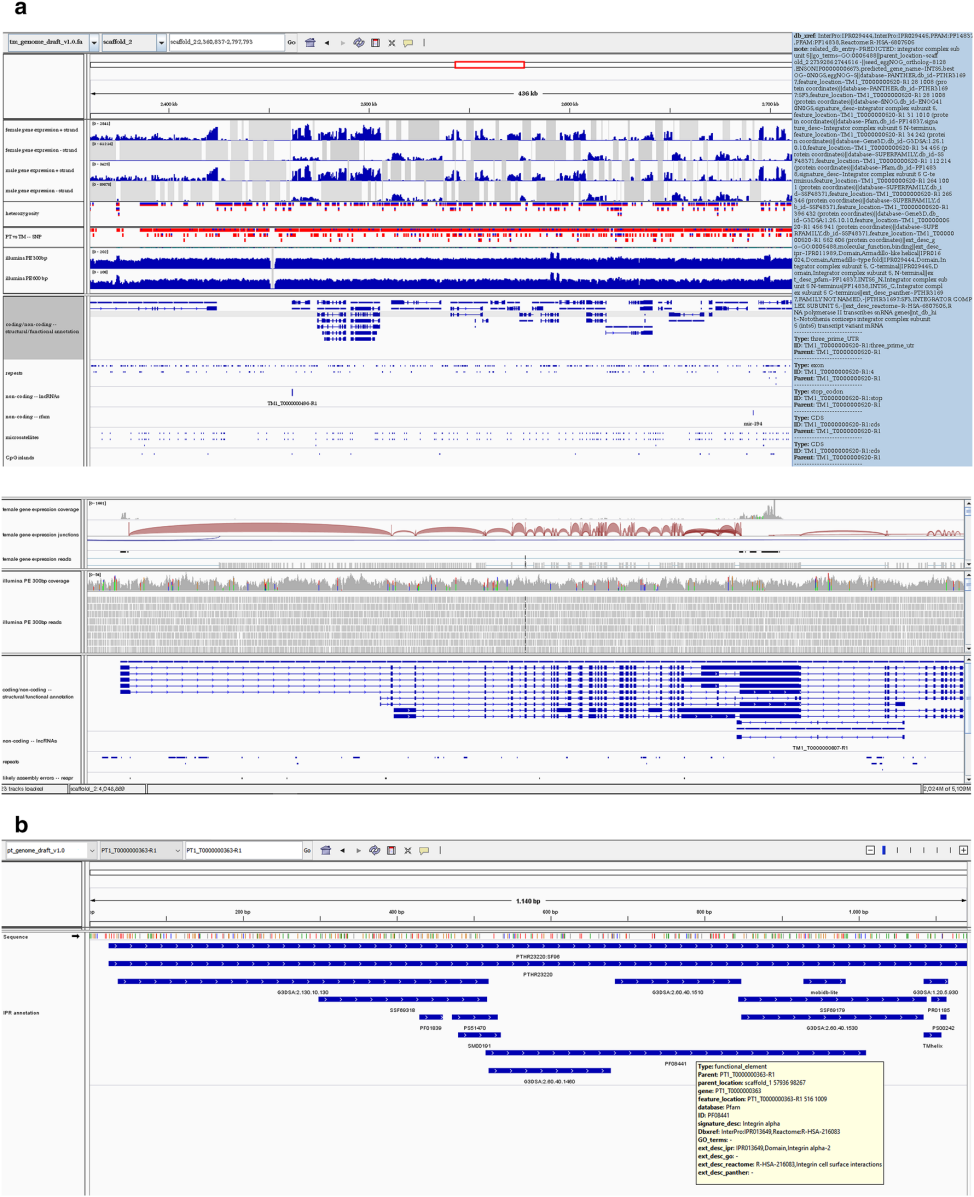
IGV tracks and extended annotation example view. (**a**) The sequences, along with structural and functional information (mouse-over), are provided via IGV tracks. We added an extensive set of data tracks and annotations (not all shown) to facilitate quick downstream analyses. (**b**) Protein sequence-based data set with annotations of identified functional domains (Figures represent screenshots from https://cichlidgenomes.tugraz.at).

The genome and transcriptome assemblies (FASTA), the structural and functional annotations (GFF3), read mappings (BAM) and additional Integrative Genomics Viewer (IGV)^33^ track files (short and long non-coding RNAs, repeats, ORFs, CpG islands, microsatellites, IPR and eggNOG domains, variant calls, read mappings, alternative splicing, and REAPR error calls; Fig. 2) are available at https://cichlidgenomes.tugraz.at.

### Quality evaluation

Assembly quality was assessed with BUSCO^34^ and CEGMA^35^. BUSCO identified 98.3% and 98% of the 4584 proteins in the Actinopterygii database in complete form for PT and TM, respectively; 1.7% and 2% of the benchmarking universal single-copy orthologs (BUSCOs) were either fragmented or missing. These results compare well with those of published genomes and are generally on a par with those of the later versions of the *O. niloticus* genome drafts (Table 5). CEGMA identified all of the 248 core eukaryotic genes (CEGs) for both PT and TM (Table 6); CEGMA results for PT and TM transcriptome assemblies can be found in Supplementary Table S6. However, REAPR reports 17,166/11,992 (PT/TM) likely assembly errors (Supplementary Table S10); there are IGV tracks highlighting questionable regions to guide caution when analyzing in the vicinity (see Fig. 2). Completeness of conserved protein domains was assessed with DOGMA^36^. DOGMA found 91.8% and 90.5% of the 1051 expected conserved domains at a conserved domain arrangement size of 1 for PT and TM, respectively (Table 7).

**Table 5:**
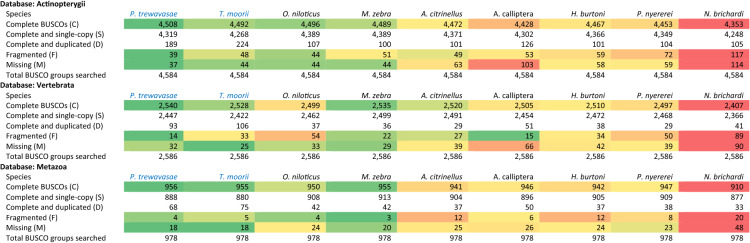
**BUSCO results:** Identified genes are classified as ‘complete’ when their lengths are within two standard deviations of the BUSCO group mean length (i.e., within ∼95% expectation). ‘Complete’ genes found with more than one copy are classified as ‘duplicated’; BUSCOs are expected to evolve under single-copy control, hence recovery of many duplicates may indicate erroneous assembly of haplotypes. Genes only partially recovered are classified as ‘fragmented’, and genes not recovered are classified as ‘missing’^34^. The latest versions of assemblies were used in all cases (i.e., V4 of *O. niloticus* and *M. zebra*). See BUSCO results for PT and TM transcriptome assemblies in Supplementary Table S5. Values are color coded according to the rank: Dark green, best; dark red, worst. BUSCO stands for benchmarking universal single-copy ortholog.

**Table 6:**
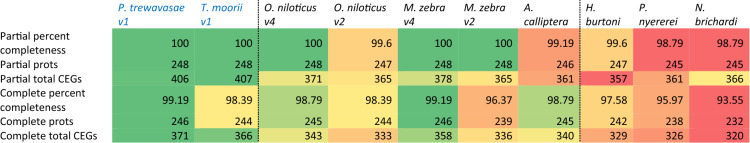
**CEGMA results:** Shown are the latest versions in all cases; for ON and MZ additionally to v4 (PacBio-based) v2 (Illumina PE + MP-based) is listed for comparison (as PT and TM were primarily constructed using the same technologies). Values are color coded according to the rank: Dark green, best; dark red, worst. CEG stands for core eukaryotic gene.

**Table 7:**
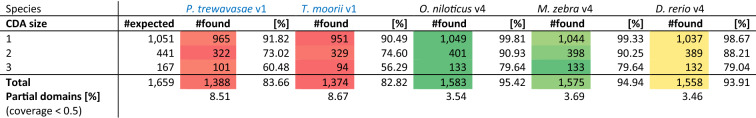
**DOGMA results:** DOGMA^36^ scores a sample transcriptome/proteome regarding its completeness of conserved protein domains provided as percentage of a defined core set (conserved domains are structural and functional building blocks of proteins). The analysis supports the notion (see mean and median protein lengths in Table 2) that gene models of protein-coding genes need improvement. Values are color coded according to the rank: Dark green, best; dark red, worst. CDA stands for conserved domain arrangement.

### Comparative analysis

We compared the genomes of PT and TM by mapping the raw reads of one species to the genome of the other species. This yielded 4,105,604 and 4,178,777 small variants (SMV; SNPs and InDels) for PT and TM, respectitvely. Furthermore, 356,428 and 577,124 SMVs were identified for PT and TM, when mapping the reads of the same species to the respective genomes. On average 1 variant per ~ 220 bp (interspecies), and 1 variant per 2540 bp (PT vs PT)/1561 bp (TM vs TM) (intraspecies) has been called (Table 8). For the two species, 93,842 and 89,489 large structural variants (SV; insertions, deletions, duplications, inversions and translocations) between species were detected, the majority being deletions with 60% and 65.6%, respectively (Table 8).

**Table 8:** Overview on inter- and intraspecies variant analysis result: The numbers represent heterogeneity between species (for PT vs TM and TM vs PT) and heterogeneity within species (for PT vs PT and TM vs TM). These numbers may include the net effect of technical issues (e.g., with assembly, annotation, mapping and calling algorithms).

Basic statistics	PT vs TM	TM vs PT	PT vs PT	TM vs TM
Genome	TM draft v1.0	PT draft v1.0	PT draft v1.0	TM draft v1.0
Data	PT filtered reads	TM filtered reads	PT filtered reads	TM filtered reads
Number of variants processed	4,105,604	4,178,777	356,428	577,124
Number of multi-allelic vcf entries	17,885	26,099	2,607	3,940
Number of variants with effects^1^	9,046,617	8,610,805	742,872	1,305,064
Genome total length	911,126,605	917,573,940	917,573,940	911,126,605
Genome effective length	906,396,323	913,305,292	905,543,956	901,211,269
Variant rate	**1 variant in 220 bases**	**1 variant in 218 bases**	**1 variant in 2540 bases**	**1 variant in 1561 bases**
**Small-scale local variants (SMV)**				
Snp	3,081,328	3,159,251	241,245	416,002
Ins	511,111	487,934	54,489	75,775
Del	513,165	531,592	60,694	85,347
Total	**4,105,604**	**4,178,777**	**356,428**	**577,124**
**Large-scale structural variants (SV)**				
Duplication	3559	2247	2870	3628
Deletion	56,396	58,692	11,989	21,047
Inversion	1853	1218	1343	1692
Translocation	18,829	12,351	11,566	15,022
Insertions	13,205	14,981	1316	2147
Total	**93,842**	**89,489**	**29,084**	**43,536**

^1^*as determined by SNPeff*^37^.

**Figure 3:**
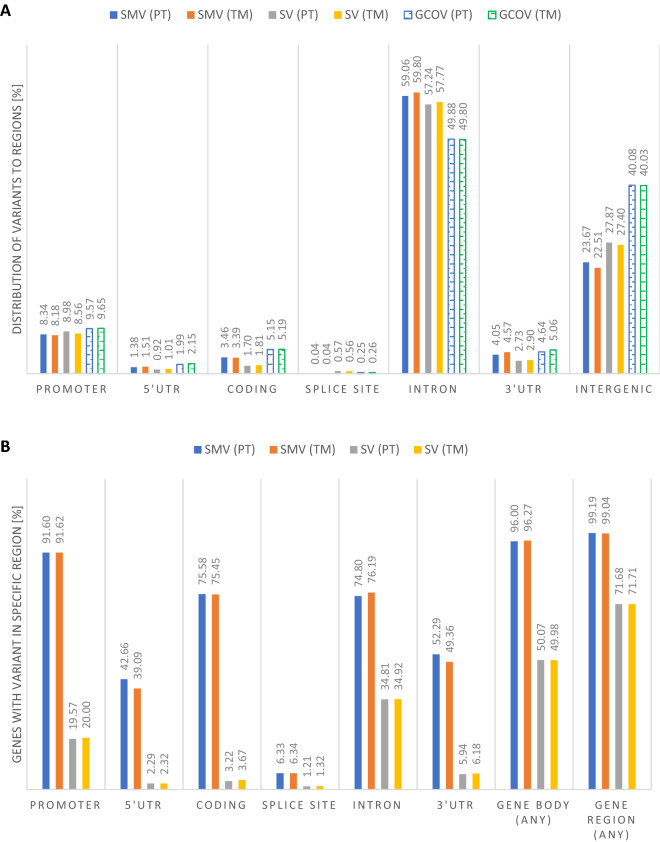
Variant location distribution and proportion of genes exhibiting a variant in a particular structural/functional region. (**A**) With respect to structural/functional regions the distribution of called small and structural variants is typical—i.e., it largely follows the proportion of the respective genomic region with respect to the entire genome. Around 60% of variants are located in gene introns, followed by ~ 25% in intergenic regions and ~ 8.5% in gene promoters. (**B**) Under the applied parameters for calling and filtering almost all genes (~ 96%) are affected by some small variant and ~ 50% by some structural variant. Interestingly, the proportions of genes exhibiting a SMV in the promoter (~ 92%; defined as 2 kbp upstream and 200 bp downstream of the TSS) or coding regions (~ 75%) are very high. Gene regions are defined as the gene body plus 5 kbp up- and downstream. SMV, small variant; SV structural variant; GCOV, genome coverage of specific region; TSS, transcription start site.

The distribution of SMV and SV observed by the comparative analysis largely follows the genome coverage of particular structural/functional regions. There are small, but noticable devations: (1) SMV are (slightly) underrepresented in promoter, 5′ UTR, coding, splice site, 3′ UTR and intergenic regions; they are overrepresented in introns; (2) SV are (slightly) underrepresented in promoter, 5′ UTR, coding, 3′ UTR and intergenic regions; they are overrepresented in introns and splice sites (via overlap) (Fig. 3A).

SNPeff^37^ categorises variant effects on the gene into four groups based on the location and nature of the variant: ‘HIGH’, ‘MODERATE ‘ ,‘LOW’, or ‘MODIFIER’, where the latter denotes non-coding variants or variants affecting non-coding genes, where predictions are difficult or there is no evidence of impact. In our analysis, more than 97% of the identified variants are classified as ‘MODIFIER’ (Table 9).

**Table 9:** Overview on putative effects of intra- and interspecies variants: Shown are variant effect annotations as determined by SNPeff^37^. The numbers represent heterogeneity between species (for PT vs TM and TM vs PT) and heterogeneity within species (for PT vs PT and TM vs TM). These numbers may include the net effect of technical issues (e.g., with assembly, annotation, mapping and calling algorithms).

	PT vs TM		TM vs PT		PT vs PT		TM vs TM	
Expected impact category	[Count]	[%]	[Count]	[%]	[Count]	[%]	[Count]	[%]
High	13,761	0.15	12,131	0.14	2348	0.32	3679	0.28
Moderate	84,185	0.93	82,142	0.95	7579	1.02	11,802	0.90
Low	149,561	1.65	142,956	1.66	11,728	1.58	19,951	1.53
Modifier	8,799,110	97.26	8,373,576	97.25	721,217	97.09	1,269,632	97.29
**Effect on coding sequences**								
Nonsense	788	0.44	791	0.45	77	0.52	132	0.54
Missense	78,503	43.48	76,737	43.35	6708	45.34	10,871	44.59
Silent	101,260	56.08	99,477	56.20	8010	54.14	13,378	54.87

Genes of interest, which are possibly affected by mutations, are highlighted in Table 10 (see Supplementary Information for details on the gene selection and GO enrichment analysis, respectively). Here, we started to analyze genes which are related to the development of the viscerocranium (untargeted gene selection) and the pharyngeal system (targeted, i.e., biased gene selection based on literature—see Table S17); however, there are other GO terms of interest which are consistently enriched over different analysis approaches such as BMP signaling, for instance. A condensed GO analysis result for an untargeted approach (A2, see Table S14b) is shown in Table 11; here, gene categories are based on variant comparison groups (within and between species groups) combined with quantile ranking and thresholding (*p* = 0.5, i.e., median), and variant counts (‘mutation loads’) were used as criterion. The term ‘embryonic viscerocranium morphogenesis’ is enriched in the within and the between species gene sets over all approaches (see Supplementary Table S16; genes belonging to this term were combined with genes from the targeted approach and used for further downstream analyses (see Supplementary Table S14a, Table S14b, Table S15, Table S16, Table S18 and Table S21). In the comparative analysis, biological species are coded as A (PT) and B (TM) (Table 11). The categories (AA, AB, BA and BB) refer to the within and between group comparisons. That is, there are mutations *at the same genomic locations* (nucleotides) which are either identical within and between species (referred to as, e.g., identical (AA) and redundantly identical (AB)) or nonidentical (referred to as, e.g., nonidentical (BB) and nonidentical (BA)); moreover, there are mutations which are unique to a group (referred to as, e.g., unique (AA) and unique (AB), i.e., at the genomic location there is only a variant in species A (unique (AA)) or there is only a variant between species A and B (unique (AB)), respectively). In the shown example, for SMV the calls for *viscerocranium morphogenesis* are symmetric except for the AB category (which fell below the threshold), i.e., the GO term is consistently enriched within and between species. Further analyses on the genes belonging to the term clearly verify the presence of shared and species-specific mutations in these genes (see example in Supplementary section *Identification of genes putatively related to facial and jaw morphology*). Hence, there is substantial variation in these genes which may drive changes in the manifestation of morphology. However, we cannot yet delineate possible effects from shared and non-shared variants.

**Table 10:** **Selected genes affected by variants.** To narrow down the list of genes carrying variants, a targeted approach and **GO enrichment analysis** were performed; this table lists **genes related to facial and jaw morphology**. Shown results are filtered and simplified: (1) variant types and locations have been unified for transcript isoforms and (annotated) gene duplicates, and (2) they have been intersected between species comparisons. SMV, small variant(s); SV, structural variant(s).

Gene	Description	Variant type	Variant location
Predicted: Insulin-Like Growth Factor-Binding Protein 3 (igfbp-3)	*IGFBP-3* plays a role in regulating pharyngeal cartilage and inner ear development and growth in zebrafish^135^	SMV	3′ UTR, intron, exon, downstream, upstream
		SV	–
Predicted: Fibroblast Growth Factor 8-Like (fgf-8)	*FGF-8* is active in mouse and rat bone cells in vitro, stimulating osteoblast proliferation in a *MAPK-*independent pathway and inhibiting osteoclastogenesis via a *RANKL/OPG*-independent mechanism^136^. Plays an important role in the regulation of embryonic development, cell proliferation, cell differentiation and cell migration. Required for normal brain, eye, ear and limb development during embryogenesis [UniProt]	SMV	3′ UTR, intron, exon, downstream, upstream, splice
		SV	–
Predicted: Barx Homeobox 1 (barx1)	*BARX1* represses joints and promotes cartilage formation in the craniofacial skeleton in zebrafish^137^	SMV	5′ UTR, 3′ UTR, intron, exon, downstream, upstream, splice
		SV	-
Predicted: T-box 10 (tbx10) **	Mutations in the *Tbx10* gene in mice and humans are thought to be a cause of isolated cleft lip with or without cleft palate^138,139^. T-box genes make major contributions to craniofacial development (***Tbx1***, ***Tbx10***, *Tbx15*, *Tbx22*) and to development of the brain (*Tbr1*, *Eomes*), mammary gland (*Tbx2*, *Tbx3*), pituitary gland (*Tbx3*, *Tbx19*), thymus (*Tbx1*), liver (*Tbx3*), lung (*Tbx2*, *Tbx4*, *Tbx5*), pigmentation (*Tbx15*) and the immune system (*Tbx21*), among others^140^	SMV	3′ UTR, intron, exon, downstream, upstream, splice
		SV	downstream
Predicted: Secreted Protein Acidic Cysteine-Rich (Osteonectin) (sparc)	*SPARC* is a cysteine-rich acidic matrix-associated protein which is required for the collagen in bone to become calcified, and it is also involved in extracellular matrix synthesis and promotion of changes to cell shape. *SPARC* is required for normal growth of zebrafish otoliths^141,142^	SMV	5′ UTR, 3′ UTR, intron, exon, downstream, upstream, splice
		SV	downstream
Predicted: Potassium Channel Tetramerization Domain Containing 15 (kctd15) *	*KCTD15* regulates neural crest formation by affecting *Wnt* signaling and the activity of transcription factor *AP-2* in zebrafish embryos and human cells^143^. In humans, expression of *KCTD15* showed a highly focal effect limited to the nasal tip^52^; *KCTD15* has been shown to regulate *TFAP2A*, which has a critical role in neural crest formation and, when mutated, results in reduced snout length in mice, among other defects. Perhaps *KCTD15* affects nasal tip shape in humans by influencing chondrocyte proliferation in the nasal septum	SMV	5′ UTR, 3′ UTR, intron, downstream, upstream
		SV	–
Predicted: T-box 1 (tbx1) **	*Tbx1* has been related to abnormal pharyngeal arch and facial development in mouse^144,145^. T-box genes make major contributions to craniofacial development (***Tbx1***, ***Tbx10***, *Tbx15*, *Tbx22*) and to development of the brain (*Tbr1*, *Eomes*), mammary gland (*Tbx2*, *Tbx3*), pituitary gland (*Tbx3*, *Tbx19*), thymus (*Tbx1*), liver (*Tbx3*), lung (*Tbx2*, *Tbx4*, *Tbx5*), pigmentation (*Tbx15*) and the immune system (*Tbx21*), among others^140^	SMV	5′ UTR, 3′ UTR, intron, exon, downstream, upstream, splice
		SV	downstream, upstream
Predicted: FAS-Associated Factor 1-Like (FAF1)	*FAF1* is disrupted in cleft palate and has conserved function in zebrafish. Knockdown of zebrafish FAF1 leads to pharyngeal cartilage defects and jaw abnormality^146^	SMV	5′ UTR, 3′ UTR, intron, exon, downstream, upstream, splice
		SV	intron
Predicted: PR Domain Containing 1 With ZNF domain (prdm1)	In zebrafish, misexpression of *prdm1* inhibits the formation of dorsoanterior structures and reduces expression of chordin, which encodes a *BMP* antagonist. Later in development *prdm1/blimp1* is expressed in many tissues, including the pharyngeal arches^147^	SMV	3′ UTR, intron, exon, downstream, upstream, splice
		SV	downstream
Predicted: Arginine-Glutamic Acid Dipeptide (RE) repeats (rere)	*RERE/Atrophin‐2* is thought to function as a transcriptional co-repressor during embryonic development in *Drosophila*^148^. This transcriptional regulator is required for the normal patterning of the early vertebrate embryo, including the central nervous system, pharyngeal arches, and limbs. Consistent with a role as a transcriptional corepressor, *RERE* binds histone deacetylase 1 and 2 (*HDAC1/2*), and orphan nuclear receptors such as *Tlx* in zebrafish^149^. It plays a role in bilateral symmetry in mice^57^ and variants are also related to craniofacial structures in humans^150^	SMV	5′ UTR, 3′ UTR, intron, exon, downstream, upstream, splice
		SV	intron, upstream
Predicted: Distal-Less Homeobox 2 (dlx2) **	The DLX proteins are postulated to play a role in forebrain and craniofacial development. The gene family has been shown to be under positive selection in East African cichlid fishes^151^. There are *DLX1-DLX2*, *DLX3-DLX4*, *DLX5-DLX6* clusters in vertebrates, linked to Hox gene clusters *HOXD*, *HOXB*, and *HOXA* respectively^152^	SMV	5′ UTR, 3′ UTR, intron, exon, downstream, upstream
		SV	–
Predicted: Retinoic Acid Receptor Gamma-A-Like (RARGA)	RARs are involved in embryonic patterning and organogenesis—with craniofacial skeletal deficiencies affecting *Rara/g*-null mutants^153^. In zebrafish combinatorial roles for retinoic acid receptors in the hindbrain, limbs and pharyngeal arches have been identified^154^	SMV	5′ UTR, 3′ UTR, intron, exon, downstream, upstream
		SV	intron
Predicted: Wingless-Type Mmtv Integration Site Family Member 9A (wnt9a)	Shown to play a role in zebrafish palate morphogenesis^155^. Ligand for members of the frizzled family of seven transmembrane receptors. Functions in the canonical *Wnt/beta-catenin* signaling pathway. Required for normal timing of IHH expression during embryonic bone development, normal chondrocyte maturation and for normal bone mineralization during embryonic bone development. Plays a redundant role in maintaining joint integrity [UniProt]	SMV	3′ UTR, intron, downstream, upstream
		SV	–
Predicted: Transforming Growth Factor Beta 2 (tgfb2)	Shown to play a role in zebrafish palate morphogenesis^155^. The majority of osteoblasts and chondrocytes in the craniofacial region are derived from cranial neural crest cells (CNCC), which produce the facial skeleton. *TGF*-β signaling plays a crucial role in craniofacial development, and loss of *TGF*-β signaling in CNCC results in craniofacial skeletal malformations^156^	SMV	Downstream, upstream
		SV	–
Predicted: Mothers Against Decapentaplegic Homolog 5 (SMAD5)	Shown to play a role in zebrafish palate morphogenesis^155^. Transcriptional modulator activated by BMP (bone morphogenetic proteins) type 1 receptor kinase. *SMAD5* is a receptor-regulated SMAD [UniProt]	SMV	5′ UTR, 3′ UTR, intron, exon, downstream, upstream
		SV	–
Predicted: Paired Box 9 (pax9)	Shown to play a role in zebrafish palate morphogenesis^155^. Transcription factor required for normal development of thymus, parathyroid glands, ultimobranchial bodies, teeth, skeletal elements of skull and larynx as well as distal limbs [UniProt]	SMV	5′ UTR, 3′ UTR, intron, exon, downstream, upstream
		SV	–
Predicted: Fibroblast Growth Factor 10-Like (FGF10)	Shown to play a role in zebrafish palate morphogenesis^155^. Plays an important role in the regulation of embryonic development, cell proliferation and cell differentiation. Required for normal branching morphogenesis [UniProt]. *FGF10*, is largely expressed in mesenchymal tissues and is essential for postnatal life because of its critical role in development of the craniofacial complex. Genetic mouse models have demonstrated that the dysregulation or absence of *FGF10* function affects the process of palate closure, the development of salivary and lacrimal glands, the inner ear, eye lids, tongue taste papillae, teeth, and skull bones. Mutations within the *FGF10* locus have been described in connection with craniofacial malformations in humans^157^	SMV	3′ UTR, intron, exon, downstream, upstream
		SV	–
Predicted: Ectodysplasin a Receptor (edar) *	*EDAR*, which has previously been linked to ear and tooth shape and hair texture, affects chin protrusion in humans^50^	SMV	5′ UTR, 3′ UTR, intron, exon, downstream, upstream
		SV	intron
Predicted: Dachsous Cadherin-Related 2 (dchs2) *	*DCHS2*, also related to cartilage, controls nose pointiness in humans^50^	SMV	Intron, exon, downstream, upstream, splice
		SV	intron
Predicted: GLI Family Zinc Finger 1 (gli1)	*GLI1* (2 and 3) are involved in craniofacial development in mice^158^	SMV	Exon, downstream, upstream
		SV	–
Predicted: GLI Family Zinc Finger 3 (gli3) *	*GLI3* known to be involved in cartilage growth is linked to the breadth of a person’s nostrils in humans^50^	SMV	5′ UTR, 3′ UTR, intron, exon, downstream, upstream, splice
		SV	intron
Predicted: Runt-Related Transcription Factor 2 (runx2) *	*RUNX2*, which drives bone development, is associated with the width of the nose bridge, the upper area of the nose in humans^50^	SMV	5′ UTR, 3′ UTR, intron, exon, downstream, upstream
		SV	–
Predicted: Paired box 1 (PAX1) *	*PAX1* known to be involved in cartilage growth is linked to the breadth of the nostrils in humans^50^	SMV	5′ UTR, 3′ UTR, intron, exon, downstream, upstream
		SV	upstream
Predicted: Paired box 3 (PAX3) *	*PAX3* influences the position of the nasion in humans^51^	SMV	5′ UTR, intron, exon, upstream
		SV	intron

*Also identified in human to play a role in craniofacial morphology.

**Other family member identified in human to play a role in craniofacial morphology.

**Table 11:**
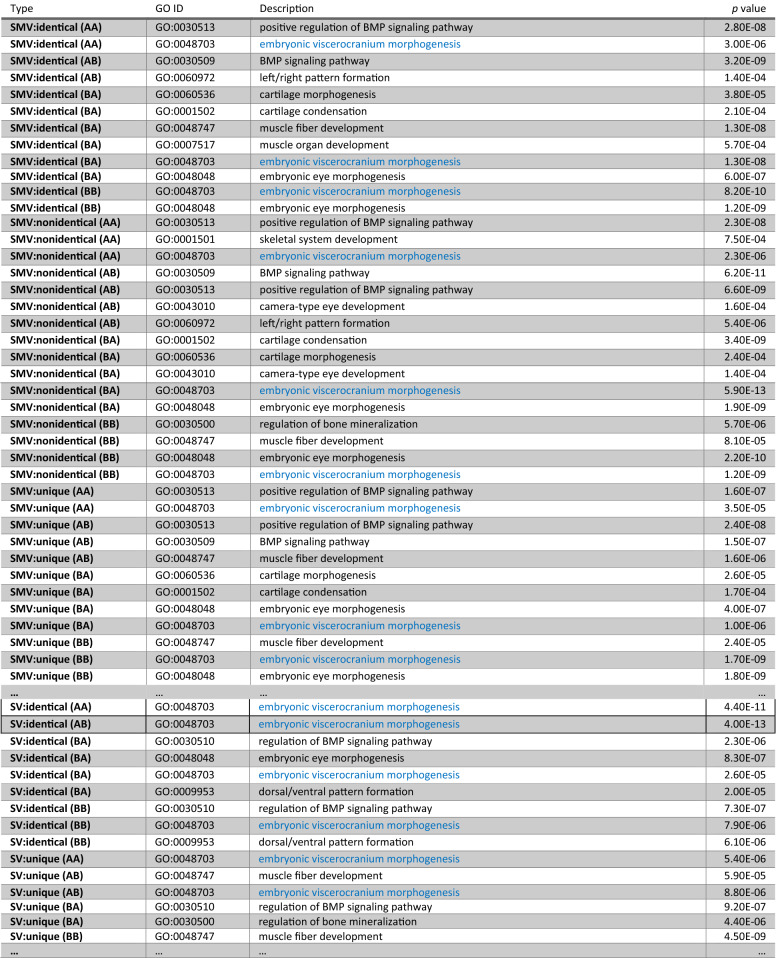
***GO enrichment analysis result—biological process terms (condensed). This table shows results*** from approach **A2** (see Supplementary Table S14b). Enrichment was assessed via a Fisher’s exact test with a cutoff of *p* ≤ 0.001 and GO topology was accounted for (R package topGO, method *weight*). In the **Type** column biological species are coded as A (PT) and B (TM); identical and nonidentical variants at same nucleotide positions, and unique variants are indicated. The categories (AA, AB, BA and BB) refer to the within and between comparison: *identical* (AA) means that the intraspecific variant(s) (SMV and SV) in this group have also been called in the related interspecific (AB) comparison at the same location, with *nonidentical* (AA) a different variant has been called at the same location (e.g., A → T within and A → G between species), with *unique* (AA) only within species A and with *unique* (AB) only between species A and B a variant was called at that position; the same holds for species B and the BB and BA categories. Comparisons have been conducted two-way, i.e., A vs B and B vs A; the groups were tested against a gene universe containing all genes with GO information (the dataset contains 7905 (PT) and 7688 (TM) GO terms in total). **SMV**: small variant(s) (SNPs and InDels); **SV**: structural variant(s) (insertions, deletions, duplications, inversions and translocations). See Supplementary Table S15 for detailed lists.

Besides variants in the DNA structure, alternative splicing (AS) was analyzed. There are ~ 6200 AS events in ~ 2600 genes between sexes of each species and ~ 39,000 AS events in ~ 9400 genes between the two species (see Supplementary Table S13).

## Discussion

### Assembly and annotation

Meta-assembly of a set of primary assemblies yielded high quality genome drafts with 918 and 911 Mbp for *Petrochromis trewavasae* and *Tropheus moori*, respectively. This is in line with the sizes between 900 and 1000 Mbp reported for other cichlid genomes^21,30^ and with the ~ 940 Mbp estimated by our assembly validation with REAPR^38^. The latest *Oreochromis niloticus* assembly spans ~ 1 Gbp;—this variation in genome size may be due to biological differences but could also indicate that some portions of the repetitive DNA in the respective genome reconstruction (PT/TM) are not included in the assembly as a consequence of sequence collapse (e.g., collapsed repeats)^39^.

Typically, assemblies are based on genomic data from a single individual, which ideally stems from an inbred line. In this project, we assembled from 6 (PT) and 5 (TM) non-inbred individuals, respectively; this called for a more complex assembly approach. Furthermore, we performed de novo sequencing without any linkage or optical map data (as seen in the latest genome drafts of *O. niloticus*, and *A. calliptera*) and the PacBio coverage was (with ~ 9–10 ×) considerably lower than that used for the assemblies of *O. niloticus* (44 × for v3^30^ and v4^27^) and *M. zebra* (16.5 × for v3^40^, added on top of already high Illumina PE and MP coverages, and 65 × for v4^27^). Still, both assemblies compare well with published genome drafts of comparable species with respect to typical metrics regarding gene content. BUSCO results (Table 5) show a low rate of ~ 4–8% (depending on database) of duplicated BUSCOs in PT and TM. This is slightly higher than the ~ 2–4% reported for other cichlid genomes, which may be a consequence of incorrect assembly of haplotypes from the non-inbred individuals. With respect to total BUSCOs identified, fragmented and missing BUSCOs, both the PT and TM genome reconstruction perform very well.

For both species, the annotations proved valid for first sensible downstream analyses, but certainly there are some gene models which may need further improvement, e.g., by repeated training of gene predictors; however, for AUGUSTUS, the central predictor, model evaluations already show good training states (see Supplementary Table S12). The most relevant sources of insufficient gene models might include gene fusions, splits and especially truncations, which are obvious under closer inspection—this is typical for early annotations, especially when the annotation pipeline is still under development. We observe a relatively low mean and median length of protein sequences (see Table 2) in both assemblies/annotations. This may reflect a systematic error in the generation process, e.g., InDels leading to frame shifts and, hence, wrong translations and premature stop codons. Investigation of this phenomenon showed non-triplet InDels; however, these are also found between, e.g., *O. niloticus* and *M.* *zebra* transcript models. Moreover, the rate of identified nonsense mutations in PT and TM is low (Table 9). The NCBI and Ensembl annotation pipelines are state-of-the-art; additionally, the amount and diversity of RNA-seq data used for the annotation of, e.g., *O. niloticus* was much larger than was the case for either species in this project. Hence, the larger number of identified transcript isoforms (as well as the higher average numbers of exons per transcript) may be seen as straight-forward consequences. However, the total number of exons in both species is on a par with the ON annotations. Interestingly, the number of gene models in PT and TM are also on a par with ON v3. As there is no well-established method to score the *correctness* of gene models (perhaps by a general structure check and a database-based similarity majority scoring), this is merely a comparison of numbers of elements, though. Moreover, there are, as mentioned, some gene fusions and splits in the PT and TM gene model sets, which will distort the gene count to some degree. As another quality measure for the annotated protein-coding genes, DOGMA^36^ and PfamScan^41^ were used; the results support the notion of bad gene models in the set, which do not contain certain protein domains or only fragments thereof (Table 7).

### Comparative analysis

We picked the two study species for the following reasons. *Tropheus moorii* is a highly successful algae browser found in large numbers in all types of rocky shore, while *Petrochromis trewavasae* is an algae grazer distributed at rocky shores on the western side of the lake, living in sympatry with *Tropheus*. The Tropheini comprise 3 predatory species, one omnivore, 10 algae browsers and 15 algae grazers. Algae grazers have chisel-like teeth to bite off filamentous algae from the rocky substrate, while algae grazers have comb-like teeth in multiple rows to comb off unicellular algae and detritus from the rocks. Due to the old age of the tribe Tropheini, amounting to about 2–6.5 myr for the onset of their radiation^6^, the degree of eco-morphological divergence is greater than in the much younger eco-morphological equivalents in Lake Victoria, but comparable with the eco-morphospace covered by the entire Lake Malawi flock. Interestingly, the genus *Tropheus* comprises about 120 mostly allopatric and in terms of color distinct populations and sister species that are morphologically similar. They all remained in the same trophic niche at all rocky shorelines throughout the lake. *Petrochtomis trewavasae* does not show much color variation, has a restricted distribution at the southwestern shoreline of the lake and is a member of a complex and morphologically distinct grazer lineage including the much more diverse *P. polyodon* species complex. When considering the entire lineage, it underwent a similar evolutionary trajectory as *Tropheus*. It should be noted here that the generally much lower species number in Lake Tanganyika when compared to Lakes Malawi and Victoria also results from the different species concepts employed, in that several allopatric entities are treated as species in Lakes Victoria and Malawi, whereas as geographical varieties in the older Lake Tanganyika radiation.

The comparative analysis presented here yielded, as expected, a large number of variant regions between the two species and even a considerable amount within each species. The large amount of variation at the intraspecific level may in fact be owed to our approach of using several non-inbred F1 individuals of a single population sampled in the natural environment, but better reflects intra-population diversity and ultimately the old evolutionary age of the lineage. We used GATK^42^ and DELLY^43^, two well established tools, for variant calling; however, the calling of variants is still not a well solved problem with often little overlap between results of different algorithmic routes (e.g., see^44,45^). As to the reported statistics on variant effects, it is known that the state of the structural annotation and the used variant effect annotator strongly influence the results^46^. The analysis results presented here reflect the state of the genome reconstructions (v1).

The relatively large number of reciprocally sorted SV and SMV among the two study species is remarkable and might reflect the relative old divergence time among the two study species amounting to about 2.5–6 Mya for the two clades^6^. In fact, it is expected that structural mutations affecting coding information need more time to evolve than regulatory mutations. Thus, when comparing species from the much younger Lake Victoria and Malawi, one would not expect such a marked degree in reciprocally distinct coding variation. The SV and SMV can also be interpreted in the light of the flexible stem hypothesis^4,18^. The flexible stem of cichlid radiations is formed by ecologically and phenotypically flexible species adapted to seasonally unstable river habitats. Once they seed lacustrine radiations, they can rapidly accommodate empty niche space in this more stable environment due to their large scope of phenotypic plasticity^18^. Subsequently, the phenotypically plastic population is subdivided into alternative adaptive phenotypes and subsequently adaptive genetic factors are sorted during speciation to proceed further via genetic accommodation and genetic assimilation^47^. Phenotypic or developmental plasticity refers to the ability of a single genotype to produce multiple phenotypes under different environmental conditions. The flexible stem hypothesis postulates that plasticity in a population can influence the direction of evolution by exposing cryptic genetic variation to selection in a novel environment. Under this model, subsets of an ancestral population exploit distinct ecological niches in a new habitat, such as different food types. Within a single generation, plasticity in anatomy may lead to a fitness increase, e.g., more efficient food capture or processing, in each niche. Newly exposed phenotypic variation will be targeted by selection, and if the new environment is stable, the plastic phenotypes may be canalized through genetic assimilation. The assumption is that the molecular mechanisms for the plastic response also underlie the evolution of key phenotypes, i.e., genetic variation in the same molecules/signaling pathways, which enable plasticity, is targeted by selection and fixed in order to canalize the phenotype. In a recent study, the role of hedgehog (Hh) signaling in the craniofacial plasticity in teleosts has been highlighted, demonstrating that Hh levels tune the sensitivity to mechanical signals related to foraging conditions—where adaptive morphological changes in immediately affected structures, e.g., the pharyngeal bones, may propagate morphological changes to other craniofacial structures^48^.

Variants have been called in virtually all gene regions. About 99% have at least one—under the applied parameter settings—possible variant in the gene body or 5 kb up/downstream (Fig. 3B). Genes with at least one mutation were subjected to Gene Ontology (GO) analysis to get hints on possible interesting functional groups affected by more variants—i.e., the number of variants (or ‘mutation load’) was used as pointer for the probability of effective changes. The rationale behind this approach was the assumption of correctness of the infinitesimal model or the omnigenic model^49^, respectively. One may expect that the observed phenotype shifts are not due to few high impact (usually coding region) variants but rather due to several ‘lower impact’ variants (in the used categories probably the ‘modifier variants’ which typically represent > 90% of the mutation load). Even if at this stage the relevance of the variation in the selected genes is not clear, all listed genes have multiple calls regarding SMV and SV (Fig. 3B) which may increase chances of effective influences on phenotypes. Given their assigned functions reported in other organisms (Table 10), however, these genes are well worth being probed. For instance, five genes being related to nose and chin shape definition (*DCHS2*, *RUNX2*, *GLI3*, *PAX1* and *EDAR*) have recently been identified in a human GWAS study^50^; several variants in all these genes have also been found between the two species. Additionally, *PAX3*, *KCTD15* and *TBX* family members (*TBX1* and *TBX10*, but not *TBX15* as previously reported) are in the result set; these genes have been related to facial morphology in humans in two other recent GWAS studies^51,52^ (Table 10). Particular focus of future downstream analyses should be on genes with stable differences in gene expression among the study species. As stated earlier, our focus lies on the differences in facial and pharyngeal shapes (see Supplementary Fig. S1). It is interesting that this simple method of unbiased variant counting (‘mutation load’) output the GO terms related to the morphogenesis of the viscerocranium reproducibly (see Supplementary Information), without giving a rather unspecific long list of GO terms. From the GO result follows the highlighting of several important signaling pathways: BMP signaling (e.g., bmp2, bmp4), Hedgehog (Hh) signaling (e.g., Shh, Gli family, Sec family, smo, med12, plcb3), endothelin signaling (e.g., edn1, furin, dlx family), retinoic acid (RA) signaling (e.g., rere, rerea), and fibroblast growth factor (FGF) signaling (e.g., fgf8, fgf20b) (see Table 10 and Supplementary Table S21). All of these signaling networks are known to play roles in the regulation of vertebrate facial morphogenesis, and they interact. There are, for instance, strong co-operative and functional interactions between Shh and retinoic acid^53–58^. A more in-depth comparative analysis of the observed gene variant distribution across the two species and its respective phenotypes was not carried out at this stage; this will be an important task for follow-up studies.

To summarize, the two new draft genomes add two monophyletic and eco-morphologically divergent key species that fill an important phylogenetic gap. Moreover, they represent the earliest offshoot of the so-called modern haplochromine cichlids, the most species-rich lineage of East African cichlids. While the Tropheini radiated within the confines of Lake Tanganyika, their allies spread over several rivers to seed additional radiations such as those in Lake Malawi and Victoria, where those reached comparable eco-morphological diversity.

## Methods

### Study species

The sampled specimens of *T. moorii* are F2 offspring of wild caught individuals from the Zambian section of the southwestern shore of Lake Tanganyika (08°38′ S 30°52′ E) near the village Nakaku, which were brought to the University of Graz in 2005. The *P. trewavasae* specimens used in this study are F1 offspring of wild fish also from the southwestern shore, but further northeast near the village Katete (08°20′S 30°30′E) and were obtained from an ornamental fish importer. Collection of the parental generation of fish was carried out in the framework of a Memorandum of Understanding between the Department of Fisheries, Ministry of Agriculture and Cooperatives, Zambia, the Department of Biological Sciences at the University of Zambia in Lusaka, the Department of Zoology at the University of Graz, Austria, the Department of Behavioural Ecology at the University of Bern, Switzerland, and the Department of Zoology at the University of Basel, Switzerland, under the research permit issued to CSt by the Zambian Ministry of Home Affairs (permit number: SP006515). Sequence data presented here are based on DNA extractions of 6 *P. trewawasae* and 5 T*. moorii* individuals; the specimens included both sexes and were about one year old.

### Sequencing and laboratory procedures

We sequenced the genomic DNA extracted from the specimens above using several sequencing technologies: Illumina HiSeq paired-end 2 × 101 bp (300 bp and 600 bp fragment size), Illumina Nextera mate-pair 2 × 100 bp (1–6 kbp fragment size), 454 Life Sciences (~ 350 bp average read length; 8 and 20 kbps fragment size) and single-molecule real-time (SMRT) sequencing technology from Pacific Biosciences (PacBio) (~ 8000–9000 bp average read length after correction).

Laboratory-related methods (DNA extraction, library preparation and sequencing) have, in part, been previously described in the accompanying paper on the mitochondrial genomes^59^. In addition, we carried out two sequencing runs using second-generation Pacific Biosciences sequencing technology based upon one individual per species. DNA extraction was carried out in Graz, library preparation and sequencing at the Lausanne Genomic Technologies Facility: DNA was sheared in a Covaris g-TUBE (Covaris, Woburn, MA, USA) to obtain 20 kbp fragments. After shearing the DNA size distribution was checked on a Fragment Analyzer (Advanced Analytical Technologies, Ames, IA, USA). 5 μg of the sheared DNA was used to prepare one SMRTbell library with the PacBio SMRTbell Template Prep Kit 1 (Pacific Biosciences, Menlo Park, CA, USA) according to the manufacturer's recommendations. The resulting library was size selected on a BluePippin system (Sage Science, Inc.; Beverly, MA, USA) for molecules larger than 11 kbp. The recovered library was sequenced on thirteen/sixteen (TM/PT) SMRT cells with P6/C4 chemistry and MagBeads on a PacBio RSII system (Pacific Biosciences, Menlo Park, CA, USA) at 240 min movie length.

For RNA-seq, total RNA from one male and female individual per species (pooled from the following tissues: liver, spleen, brain, heart and skeletal muscle) was extracted with Trizol as follows: tissue was homogenized with MagnaLyser and incubated with Trizol-tube 5 min at room temperature (RT); 200 µl Chloroform (/ml of Trizol) was added and shaken vigorously for 15 s, incubated for 2–3 min/RT and centrifuged at 12,200 rpm/4 °C/15 min; supernatant was transferred to a new 1.5 ml tube and 500 µl isopropanol (/ml of Trizol) were added; after vortexing, incubation for 10 min/RT, centrifugation at 12,200 rpm/4 °C/10 min supernatant was discarded and the pellet placed on ice immediately. The pellets were washed 2 times: add 1 ml EtOH 80% (–20 °C), centrifuge: full speed/4 °C/5 min discard supernatant and finally dried at 37 °C. Dried pellets were resuspended in 20 µl distilled water. RNA-seq libraries were derived from total RNA which was rRNA-depleted, normalized and sequenced on a single Illumina HiSeq 2500 lane per species.

### General data (pre)processing

All pipelining and higher-level processing was done with R/Bioconductor, some minor pipelining in Bash and some workhorse functionality was written in C +  + (called from R). For details on parameter settings for important steps/tools see Supplementary Table S22.

FastQC v0.10.1^60^ was used for basic read quality evaluation. A custom k-mer spectrum-based approach using JELLYFISH v2.0^61^ (in conjunction with a database of known technical sequences) and a *De Bruijn*-based approach (implemented in Minion from the Kraken v13-274 package^62^) were used for the automatic identification of technical contaminants and suspicious sequences (based on expected frequencies). In addition, FastQScreen v0.4.4^63^ was utilized for the species-specific identification of biological contamination and DeconSeq v0.4.3^64^ for its removal. Cutadapt v1.5^65^ was used for the removal of technical contaminants, Scythe v0.994^66^ for additional 3′ adapter trimming, CLC quality trim v4.2^67^ for quality-score-based read trimming and Reaper v13-274^62^ for further quality and complexity-based filtering. BBmerge v33.40^68^ was used for overlapping paired-end read merging and FastUniq v1.1^69^ for duplicate removal. Nextclip v1.2^70^ was used for Nextera mate-pair read filtering and classification. 454 datasets were additionally filtered with sffToCA (Celera Assembler utility). BAMtools v2.4.0^71^, SAMtools/BCFtools/HTSlib v1.4^72^ and Picard tools v1.119^73^ were used for mapping and sequence file manipulations such as indexing, merging, sorting, and generation of subsets, removal of duplicate reads, and removal of PE contamination from MP libraries in sequence files. Proovread v2.13.10^74^ was used for PacBio read correction utilizing all available Illumina PE data and the *unitigs* created by MaSuRCA v2.3.2^75^. SEECER v0.1.3^76^ and Rcorrector v1.0.2^77^ were used for RNA-seq and Musket v1.1^78^ for DNA-seq base-call correction. DNA-seq and RNA-seq datasets were preprocessed using the same pipeline (with different parameter settings); in general, two filter regimes were applied to each data set (‘stringent’/’standard’ and ‘relaxed’) in preparation for different downstream use cases (see Supplementary Table S22). Genome sizes were estimated by a k-mer spectrum-based approach implemented in GCE v1.0.2^79^.

### Genome assembly

From the perspective of the conducted meta-assembly, the algorithm implemented in MaSuRCA v2.3.2^75^ (utilizes Celera Assembler v6.5^80^) served as the core assembly procedure; all at this time available data sets (i.e., Illumina PE and MP, Illumina Nextera MP and 454 MP and SE) were used. Celera Assembler v8.3rc2 (CA)^80^ was used for the ‘PacBio only’ assemblies. As several individuals per species (all non-inbred diploids) have been sequenced in this project, heterozygosity was a concern. Hence, assembly algorithms specifically designed to better handle divergence were incorporated into the reconstruction approach: Platanus v1.2.1^81^ is a recent assembler tailored to more sensibly deal with heterozygosity issues in genomic data (5 iterations; all Illumina data sets were used); Redundans v0.12c^82^ (utilizes SSPACE3^83^, GapCloser^84^, bwa^85^ and last^86^) also aims at providing more accurate and contiguous assemblies of highly heterozygous genomes (5 iterations; all Illumina data sets were used). The PBJelly Suite v15.8.24^87^ (utilizes BLASR^88^) was used to incorporate the long sequence reads (PacBio) in a reference-guided assembly process into the established drafts (5 iterations). The diverse set of generated genome drafts was subjected to Metassembler^89^ in an attempt to generate high quality consensus sequences. A custom algorithm, which takes into account several measures on probable misassemblies, contiguity and gene predictions (drawing information from QUAST^90^ and REAPR^38^), was applied to determine the best order of successive meta-assemblies.

### Genome finishing

For another round of inter-scaffold gap closing, GMcloser^91^ (utilizes Nucmer^92^ / BLAST^93^ and Bowtie2^94^) was applied on the meta-assemblies with PacBio and Illumina PE data. Finally, Sealer^95^ (utilizes *Konnector*, a part of the ABYSS assembler pipeline^96^) was used with the Illumina PE (liberal) libraries for final gap filling and a custom GATK-based^42^ genome finishing (via Illumina PE back mapping and consensus recalling) was applied.

### Genome validation

REAPR v1.0.18^38^ (utilizing SMALT v0.7.0.1^97^) was used with the Illumina Nextera mate-pair (6 kbp) and Illumina PE (600 bp) libraries to evaluate the correctness of assemblies and QUAST v4.1^90^ was applied for contiguity and gene prediction statistics. Completeness of the assemblies was assessed using CEGMA v2.5^35^ (utilizing GeneWise v2.4.1^98^, HMMER v3.0^99^ and NCBI BLAST + v2.2.29 + ^93^) with parameter optimization for vertebrate genomes (–vrt) and BUSCO v3.0.2^34^ (utilizing NCBI BLAST + v2.2.29 + , HMMER v3.1^99^ and AUGUSTUS v3.2.1^100^).

### Transcriptome assembly and RNA-seq read mapping

The transcriptome assemblies were conducted with Trinity v2.3.2^101,102^ and the PASA2 v2.0.2 pipeline^103^ (utilizing GMAP v2014-12–06^104^, BLAT v36.1^105^ and MySQL v5.7.12^106^); also Transdecoder v3.0.1^102^ was applied to identify candidate coding regions (used with MAKER3^107^). RNA-seq read alignments for other analyses were generally conducted with STAR v2.4.2a^108^ using default parameters.

### Genome annotation

Structural annotations were performed based on experimental data from mRNA-Seq datasets. Additionally, information was drawn from transcript and protein models from selected publicly available datasets (*Danio* *rerio*, *H. burtoni, M. zebra, N. brichardi, O. niloticus*, and *P. nyererei*) and from further models in UniProt|Swiss-Prot, nr/nt and UniRef90|teleost. Functional annotation was primarily conducted via BLAST-based comparisons against mentioned databases and via a host of databases coordinated by InterProScan 5 (see Table 2).

Structural annotation of coding genes and tRNAs was generated using the pipelines MAKER v3.0^107^ (utilising the gene finders GeneMark-ES v4.32^109^, AUGUSTUS v3.2.1^100^, SNAP v2013-11–29^110^ and tRNAscan v1.3.1^111^), Funannotate v0.5.5-v0.7.0^112^ (FA) and BRAKER1 v1.9^113^ (utilising GeneMark-ET v4.32^114^ and AUGUSTUS v3.2.1^100^); BRAKER1 was also used for AUGUSTUS training. In addition, gene models were created with StringTie v1.3.2d^115^ and Cufflinks v2.2.1^116^. All models were combined by EVidenceModeler v1.1.1^117^ (EVM) under the control of MAKER3. For non-coding RNAs, Infernal v1.1.2^118^, Rfam v12.1^119^ and FEELnc v0.1.0^120^ were utilized. The mRNA training set for FEELnc was derived from the FA/MAKER annotation data, where presumed ‘good’ gene models with similar structure to previously published models were selected; the lncRNA training set was generated by shuffling of the mRNA sequences. Microsatellites were called with MISA v1.0^121^, CpG islands with EMBOSS v6.6.0^122^ cpgplot and ORFs with EMBOSS v6.6.0 getorf (and R post-processing). Repeats were determined using RepeatMasker v4.0.6^32^ (with RepBase v20160321^123^ and species-specific libraries generated with RepeatModeler v1.0.8^124^), RepeatScout v1.0.5^125^ and TRF v406^126^.

Functional annotation was conducted using InterProScan v5.24–63.0^127^ (utilizing the databases CDD-3.14, Coils-2.2.1, Gene3D-3.5.0, Hamap-201605.11, MobiDBLite-1.0, PANTHER-11.1, Pfam-30.0, PIRSF-3.01, PRINTS-42.0, ProDom-2006.1, ProSitePatterns-20.119, ProSiteProfiles-20.119, SFLD-2, SMART-7.1, SUPERFAMILY-1.75, TIGRFAM-15.0 and TMHMM-2.0c). Furthermore, under the control of FA the databases eggNOG v4.5.1^128^ (fiNOG), MEROPS v12.0^129^, dbCAN v5.0^130^ and BUSCO vertebrata v3^34^ were used for similarity searches and SIGNALP v4.1^131^ for identification of target location signal sequences.

Final integration of all annotations was done with R 3.4.3/Bioconductor 3.6 using the packages data.table 1.12.2, GenomicFeatures 1.30.3, VariantAnnotation 1.24.5 and their dependencies.

### DNA-seq read mapping

Preprocessed reads were aligned in paired‐end mode with BWA mem^85^ using the default parameters with ‐M and ‐R flags. Aligned reads were coordinate sorted with Picard SortSam v1.119^73^ and indexed with SAMtools index v1.4^72^. Duplicates were removed with Picard MarkDuplicates v1.119. The quality of the mappings was assessed with QualiMap v2.0^132^.

### Comparative analysis—small (SMV) and structural variant (SV) calling—variant effect prediction

The Genome Analysis Toolkit (GATK) v3.7 was used for local realignment of reads and the detection and filtering of SNP/InDel variants (referred to as small variant/s, SMV)^42^ as recommended by the GATK documentation; the HaplotypeCaller was applied—with a minimum score for variant emission of 10, for calling of 30, and a minimum pruning of 10. SMV with a quality score ≥ 30 were included in further analyses. DELLY v0.7.7^43^ was applied to call structural variant/s (SV, insertions, deletions, duplications, inversions and translocations) with an insert size cut‐off of 3 (for deletions) and a minimum paired‐end mapping quality of 20. All variants with a minimum of 5 broken read pairs supporting the variant as well as with a minimum length of 300 bp (for deletions, inversions, and duplications) were included in further analyses, as recommended by the DELLY documentation. Presumed variant effects were called with SNPeff v4.3r^37^. Whippet v0.11.1^133^ was used for the calling of alternative splicing events. The comparative analyses were conducted in R 3.4.3/Bioconductor 3.6 using the packages data.table 1.12.2, GenomicFeatures 1.30.3, VariantAnnotation 1.24.5 and their dependencies.

### GO analysis

To narrow down the candidate gene list, GO enrichment analysis was performed on the gene regions carrying variants using the R package topGO v2.30.1^134^; the custom GO annotations were generated based on the InterProScan mappings. GO topology was accounted for (method *weight*) and enrichment was assessed via a Fisher’s exact test with a cutoff of *p* ≤ 0.001. See details on the GO analysis in the Supplementary Information.

### Ethics approval and consent to participate

Animal treatment reported in this paper complies with the standards of the Animal Welfare Act in Austria and the European Community Directive 86/609. Fish were kept in our certified aquarium facility at the Institute of Biology, University of Graz. Individuals were sampled by CSt and SK, euthanized using an overdose of clove oil and decapitated conforming to the Austrian Animal Welfare legislation. According to the Austrian Animal Experiments Acts (TVG, BGBI. Nr. 501/1989, last changed by BGBI. I Nr. 162/2005), approval was not required because no experimental treatment was performed.

### Consent for publication

Not applicable.

## Supplementary Information

Supplementary Information.

## Data Availability

The genome drafts were uploaded to EBI, TM: [GCA_902810505], PT: [GCA_902810495]; the genome and transcriptome assemblies (FASTA), the structural and functional annotations (GFF3), read mappings (BAM) and additional IGV^33^ track files are available at https://cichlidgenomes.tugraz.at.
