# Supplementary material for: Genome sequences of Tropheus moorii and Petrochromis trewavasae, two eco-morphologically divergent cichlid fishes endemic to Lake Tanganyika
Source: Sci Rep. 2021 Feb 22;11:4309. doi: 10.1038/s41598-021-81030-z (PMC7900123; doi:10.1038/s41598-021-81030-z)
Supplement: Supplementary file 1 — Supplementary Information. [file 41598_2021_81030_MOESM1_ESM.pdf]

Supplementary Information

Genome sequences of *Tropheus moorii* and *Petrochromis trewavasae*, two eco-morphologically divergent cichlid fishes endemic to Lake Tanganyika

Fischer, C.,[1,2] Koblmüller, S.,[1] Börger, C.,[1] Michelitsch, G.[3], Trajanoski, S.,[3] Schlötterer, C.,[4] Guelly, C.,[3] Thallinger, G. G.[2,5*] & C. Sturmbauer[1,5*]

[1] Institute of Biology, University of Graz, Graz, Austria

[2] Institute of Biomedical Informatics, Graz University of Technology, Graz, Austria

[3] Center for Medical Research, Medical University of Graz, Graz, Austria

[4] Institut für Populationsgenetik, Vetmeduni Vienna, Vienna, Austria

[5] BioTechMed-Graz, Graz, Austria

* corresponding authors

Petrochromis trewavasae

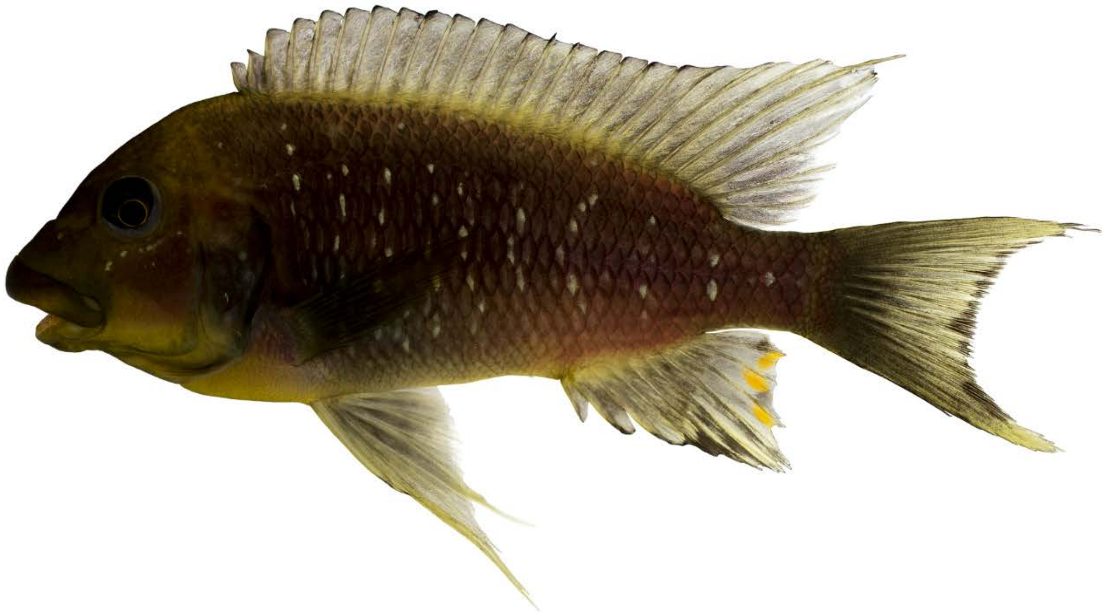

Tropheus moorii

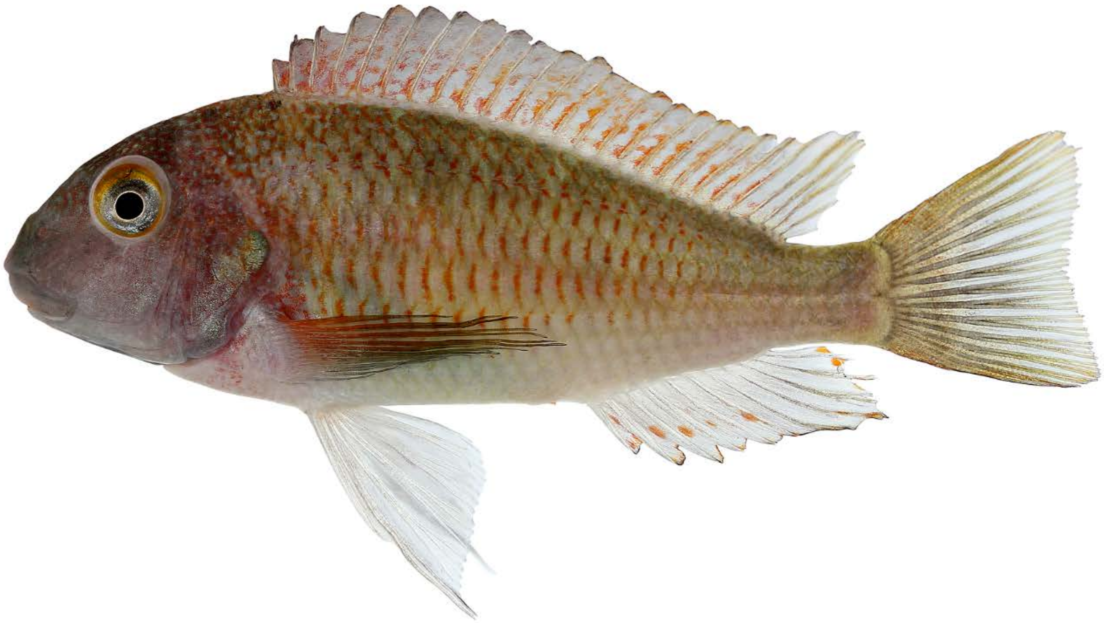

Figure S1 Species morphologies. Shown are *Petrochromis trewavasae* (PT) and *Tropheus moorii* Nakaku (TM) specimens in lateral view. These species are evolutionary closely related. Notice the distinct shapes of the viscerocrania. Images courtesy of Wolfgang Gessl.

Table S1 Gene length distributions

	P. trewavasae	*T. moorii*
**Quantile [%]**	**length [bp]**	**length [bp]**
**50**	5,360	5,519
**80**	18,390	18,897
**90**	35,343	35,824
**95**	59,130	59,568
**99**	146,750	146,035
**100**	619,290	860,082

Table S2   Statistics for different *Oreochromis niloticus* assembly versions

**Species/version**	***O. niloticus* v4**	***O. niloticus* v3**	***O. niloticus* v2**
**Sequence length [bp]**	1,005,681,550	1,009,856,516	927,679,487
**Ungapped length [bp]**	1,005,626,550	1,005,626,516	816,068,047
**Scaffolds**	2,460	2,567	5,909
**Scaffold N50 [bp]**	38,839,487	37,007,722	2,766,223
**Scaffold L50**	11	12	96
**Contigs**	3,010	2,990	77,754
**Contig N50 [bp]**	2,923,640	3,090,215	29,493
**Contig L50**	96	93	6,912

Table S3 Information on lncRNAs

	P. trewavasae	*T. moorii*
**Location/orientation**	**[%]**	**[%]**
**Containing**	32.94	34.86
**Convergent**	10.16	8.97
**Divergent**	10.26	8.68
**Nested**	15.79	14.70
**Overlapping**	8.13	10.65
**Same strand**	22.72	22.14
**Antisense**	42.28	36.82
**Sense**	57.72	63.18
**Intergenic**	43.14	39.79
**Genic**	56.86	60.21
**Upstream**	21.35	19.99
**Exonic**	48.18	52.95
**Intronic**	8.68	7.26
**Downstream**	21.79	19.80
**# of lncRNAs**	2,782	2,112

Table S4 CEGMA results for different assembly versions. Values are color coded according to the rank: Dark green, best; dark red, worst.

	P. trewavasae	*T. moorii*	*O. niloticus v4*	*O. niloticus v3*	*O. niloticus v2*	*M. zebra v4*	*M. zebra v3*	*M. zebra v2*
**Partial percent completeness**	100	100	100	100	99.60	100	100	100
**Partial prots**	248	248	248	248	247	248	248	248
**Partial total CEGS**	406	407	371	367	365	378	369	365
**Complete percent completeness**	99.19	98.39	98.79	98.79	98.39	99.19	98.39	96.37
**Complete prots**	246	244	245	245	244	246	244	239
**Complete total CEGS**	371	366	343	342	333	358	345	336

Table S5 BUSCO results for transcriptome assemblies

**Database: Actinopterygii**				
**Species**	*Petrochromis trewavasae*		*Tropheus moorii*	
**Assembler**	**PASA**	**Trinity**	**PASA**	**Trinity**
**Complete BUSCOs (C)**	4,015	3,465	4,054	3,764
**Complete and single-copy BUSCOs (S)**	1,619	98	1,453	87
**Complete and duplicated BUSCOs (D)**	2,396	3,367	2,601	3,677
**Fragmented BUSCOs (F)**	280	487	261	352
**Missing BUSCOs (M)**	289	632	269	468
**Total BUSCO groups searched**	4,584	4,584	4,584	4,584
**Database: Vertebrata**				
**Species**	*Petrochromis trewavasae*		*Tropheus moorii*	
**Assembler**	**PASA**	**Trinity**	**PASA**	**Trinity**
**Complete BUSCOs (C)**	2,340	2,104	2,362	2,217
**Complete and single-copy BUSCOs (S)**	869	58	770	42
**Complete and duplicated BUSCOs (D)**	1,471	2,046	1,592	2,175
**Fragmented BUSCOs (F)**	181	343	162	260
**Missing BUSCOs (M)**	65	139	62	109
**Total BUSCO groups searched**	2,586	2,586	2,586	2,586
**Database: Metazoa**				
**Species**	*Petrochromis trewavasae*		*Tropheus moorii*	
**Assembler**	**PASA**	**Trinity**	**PASA**	**Trinity**
**Complete BUSCOs (C)**	963	945	959	953
**Complete and single-copy BUSCOs (S)**	361	7	315	5
**Complete and duplicated BUSCOs (D)**	602	938	644	948
**Fragmented BUSCOs (F)**	12	32	12	21
**Missing BUSCOs (M)**	3	1	7	4
**Total BUSCO groups searched**	978	978	978	978

Table S6 CEGMA results for transcriptome assemblies. Values are color coded according to the rank: Dark green, best; dark red, worst.

	**PASA transcriptome**		**Trinity transcriptome**	
	P. trewavasae	*T. moorii*	*P. trewavasae*	*T. moorii*
**Partial percent completeness**	100	99.6	98.79	100
**Partial prots**	248	247	245	248
**Partial total CEGS**	1,070	1,066	1,220	1,238
**Complete percent completeness**	97.58	98.39	95.16	96.37
**Complete prots**	242	244	236	239
**Complete total CEGS**	902	937	1,141	1,176

Table S7 Augustus model parameter evaluation for BUSCO

BUSCO utilizes the Augustus gene predictor as central functional element. As we have trained Augustus for PT and TM but not for the other published genomes, we used the publicly available (well trained) model for *D. rerio* for all species. This table shows the effect of substituting the model in the cases of PT and TM. We see that the *D. rerio* model performs slightly better in recognizing *complete* BUSCOs but slightly worse in finding them (i.e., there are more missing BUSCOs) with the most specific database (Actinopterygii) which changes to performing worse on identifying complete BUSCOs with more general databases (Vertebrata and Metazoa). However, the approach of model substitution seems valid with respect to sensitivity and specificity shifts – especially, as the number of complete BUSCOs is of main interest.

Augustus model parameter evaluation

Database: actinopterygii

	Danio rerio	*Petrochromis trewavasae*	$\Delta_{rel\%}$	*Danio rerio*	*Tropheus moorii*	$\Delta_{rel\%}$
**Complete BUSCOs (C)**	4508	4484	-0,532	4492	4479	-0,289
**Complete and single-copy BUSCOs (S)**	4319	4288	-0,718	4268	4250	-0,422
**Complete and duplicated BUSCOs (D)**	189	196	3,571	224	229	2,183
**Fragmented BUSCOs (F)**	39	68	42,647	48	68	29,412
**Missing BUSCOs (M)**	37	32	-13,514	44	37	-15,909
**Total BUSCO groups searched**	4584	4584		4584	4584	

Database: vertebrata

	Danio rerio	*Petrochromis trewavasae*	$\Delta_{rel\%}$	*Danio rerio*	*Tropheus moorii*	$\Delta_{rel\%}$
**Complete BUSCOs (C)**	2540	2539	-0,039	2528	2524	-0,158
**Complete and single-copy BUSCOs (S)**	2447	2449	0,082	2422	2416	-0,248
**Complete and duplicated BUSCOs (D)**	93	90	-3,226	106	108	1,852
**Fragmented BUSCOs (F)**	14	32	56,250	33	47	29,787
**Missing BUSCOs (M)**	32	15	-53,125	25	15	-40,000
**Total BUSCO groups searched**	2586	2586		2586	2586	

Database: metazoa

	Danio rerio	*Petrochromis trewavasae*	$\Delta_{rel\%}$	*Danio rerio*	*Tropheus moorii*	$\Delta_{rel\%}$
**Complete BUSCOs (C)**	956	961	0,520	955	963	0,831
**Complete and single-copy BUSCOs (S)**	888	892	0,448	880	886	0,677
**Complete and duplicated BUSCOs (D)**	68	69	1,449	75	77	2,597
**Fragmented BUSCOs (F)**	4	3	-25,000	5	4	-20,000
**Missing BUSCOs (M)**	18	14	-22,222	18	11	-38,889
**Total BUSCO groups searched**	978	978		978	978	

Table S8 BUSCO result comparison for different assembly versions. Values are color coded according to the rank: Dark green, best; dark red, worst.

Database: Actinopterygii

**Species**	*P. trewavasae v1*	*T. moorii v1*	*O.niloticus v4*	*M.zebra v4*	*O. niloticus v3*	*M. zebra v3*	*O. niloticus v2*
**Complete BUSCOs (C)**	4,508	4,492	4,496	4,483	4,492	4,489	4,503
**Complete and single-copy (S)**	4,319	4,268	4,389	4,326	4,388	4,389	4,398
**Complete and duplicated (D)**	189	224	107	157	104	100	105
**Fragmented (F)**	39	48	44	45	41	51	37
**Missing (M)**	37	44	44	56	51	44	44
**Total BUSCO groups searched**	4,584	4,584	4,584	4,584	4,584	4,584	4,584

* common Augustus model: *Danio rerio*

Database: Vertebrata

**Species**	*P. trewavasae v1*	*T. moorii v1*	*O.niloticus v4*	*M.zebra v4*	*O. niloticus v3*	*M. zebra v3*	*O. niloticus v2*
**Complete BUSCOs (C)**	2,540	2,528	2,499	2,539	2,536	2,535	2,540
**Complete and single-copy (S)**	2,447	2,422	2,462	2,467	2,499	2,499	2,511
**Complete and duplicated (D)**	93	106	37	72	37	36	29
**Fragmented (F)**	14	33	54	13	19	22	16
**Missing (M)**	32	25	33	34	31	29	30
**Total BUSCO groups searched**	2,586	2,586	2,586	2,586	2,586	2,586	2,586

* common Augustus model: *Danio rerio*

Database: Metazoa

**Species**	*P. trewavasae v1*	*T. moorii v1*	*O.niloticus v4*	*M.zebra v4*	*O. niloticus v3*	*M. zebra v3*	*O. niloticus v2*
**Complete BUSCOs (C)**	956	955	950	952	950	955	951
**Complete and single-copy (S)**	888	880	908	884	908	913	913
**Complete and duplicated (D)**	68	75	42	68	42	42	38
**Fragmented (F)**	4	5	4	4	4	3	3
**Missing (M)**	18	18	24	22	24	20	24
**Total BUSCO groups searched**	978	978	978	978	978	978	978

* common Augustus model: *Danio rerio*

Table S9 (Back) mapping results. (Back)mappings of all relevant libraries to the genome drafts show high mapping percentages, suggesting a good reconstruction with respect to available data. Mappings were performed with BWA mem (DNA-seq), BLASR (DNA-seq, PacBio) and STAR (RNA-seq).

	P. trewavasae		***T. moorii***	
**PacBio BWA**	99.98	%	99.97	%
**PacBio BLASR**	99.95	%	99.95	%
**Illumina PE 300bp**	99.54	%	99.49	%
**Illumina PE 600bp**	99.52	%	99.51	%
**Illumina SE**	99.42	%	99.25	%
**Illumina MP 1-3k**	98.22	%	96.97	%
**Illumina MP 3-6k**	97.51	%	96.66	%
**RNA-seq male**	95.96	%	95.98	%
**RNA-seq female**	93.97	%	96.19	%

Table S10 REAPR results. Both assemblies still have a number of assembly errors which may need additional data for correction. Some errors can be problematic with respect to downstream analyses, hence there are REAPR [10] IGV tracks (GFF files) available for (visual) support when analyzing features in the vicinity.

	P. trewavasae	***T. moorii***
**FCD errors within a contig**	3,378	1,760
**FCD errors over a gap**	9,521	9,120
**Low fragment coverage within a contig**	1,688	396
**Low fragment coverage over a gap**	2,579	716
	**17,166**	**11,992**

Table S11 Genome size estimations. This table shows the genomes sizes as obtained by assemblies and estimated by k-mer spectra analysis (using GCE).

	k-mer spectra-based predictions		***reconstructions***		***final reconstruction***	
**Species**	**PT**	**TM**	**PT**	**TM**	**PT**	**TM**
**Size/mean [Mbp]**	**874**	**877**	**856**	**833**	**917**	**911**
**Stdev [Mbp]**	12	9	62	67	-	-
**Min [Mbp]**	860	866	779	754	-	-
**Max [Mbp]**	888	886	966	952	-	-

Table S12 AUGUSTUS training evaluation. This table shows the sensitivity and specificity values for the gene predictor AUGUSTUS for the different levels of organization; these values indicate well trained hidden Markov models (HMMs).

	**nucleotide level**		**exon level**		**gene level**	
**species**	**sensitivity**	**specificity**	**sensitivity**	**specificity**	**sensitivity**	**specificity**
PT	0.961	0.966	0.844	0.827	0.652	0.631
TM	0.962	0.968	0.864	0.847	0.648	0.633

Table S13 Alternative splicing results. This table shows the numbers of called alternative splicing (AS) events (as determined by Whippet) and the number of genes affected by AS for intraspecific comparisons (male vs female) and interspecific (male+female species A vs male+female species B). There are ~6,200 AS events in ~2,600 genes between sexes and ~39,000 AS events in ~9,400 genes between species.

	**intraspecific (male vs female)**		**interspecific**	
**Species**	**AS genes**	**AS events**	**AS genes**	**AS events**
PT	2,732	6,225	9,591	38,841
TM	2,559	6,292	9,189	39,260

Identification of genes putatively related to facial and jaw morphology (Table 11)

In this downstream analysis the variant calls were used. As virtually all gene regions do carry variants, the genes with at least one mutation were subjected to Gene Ontology (GO) analysis to get hints on possible interesting functional groups affected by more variants – i.e., the number of variants was used as pointer for the probability of effective changes. The GO categories for the Fisher's exact test were either created by the type assignment only (see below) or by type assignment and additional grouping according to a quantile threshold – the gene universe consisted of all gene models with a GO annotation. For the quantile approaches, the number of variants per gene, i.e., the 'mutation load' was used for ranking. The predicted effect impacts were also incorporated in some approaches but were eventually not used; however, the analyses incorporating a count weighting based on predicted effect category (high, moderate, low, modifier) (via coefficients and/or logarithmic scaling) yielded similar results with respect to the GO terms of interest (facial development) – when the weight-based skewing was within reason. The rationale behind the decision to use unweighted counts was the assumption of correctness of the infinitesimal model or the omnigenic model, respectively. One may expect that the observed phenotype shifts are not due to few high impact (usually coding region) variants but rather due to several 'lower impact' variants (in the used categories probably the 'modifier variants' which typically represent >90% of the mutation load). Moreover, the weights assigned to variants of the different categories could only have been arbitrary – with an arbitrary degree of introduced bias. For the same reason, variant counts were eventually not normalized on gene length. This may bias the GO analysis towards longer genes which statistically carry more mutations, but this bias is a biological one – and hence we welcome it. Still, also analyses with gene length normalization were conducted; again, the GO terms of interest were present but less prominent; generally, as can be expected, the normalization changes the GO term profile considerably.

Enrichment was assessed via a Fisher's exact test with a cutoff of $p<=0.001$ and GO topology was accounted for (R package topGO, method *weight*). In the following, compared biological species are coded as A and B; identical and nonidentical variants at same nucleotide positions (within and between species), and unique variants (only seen within or between species) have been determined.

Comparisons have been conducted two-way, i.e., A vs B and B vs A. The categories used below (AA, AB, BA and BB) refer to the within and between comparison; identical (AA) means that the intraspecific variants (SMVs and SVs) in this group have also been called in the related interspecific (AB) comparison at the same location, with nonidentical (AA) a different variant has been called at the same location (e.g., A→T within and A→G between species), with unique (AA) only within species A and with unique (AB) only between species A and B a variant was called at that position; the same holds for species B and the BB and BA categories (Table S14a).

Table S14a Gene-level statistics on the two-way comparison of species (A vs B and B vs A). This table shows between (AB and BA) and within species (AA and BB) variant statistics regarding the gene level (with base-resolution in the variant location comparison). ***AB unique***, for instance, refers to a variant which is present between species A and B (AB) but not within species A (AA) in the respective gene at the same position; ***AB & AA*** refers to genes which exhibit the same variants at the same locations between and within species. SMV, small variant/s; SV, structural variant/s.

Type	# variants (AB)	# variants (AA)	# genes (AB)	# genes (AA)	# genes (AB unique)	# genes (AA unique)	# genes (AB & AA)
**SMV**	4,026,311	227,454	34,942	26,580	8,675	313	26,267
**SV**	85,705	25,306	35,915	9,884	31,230	5,199	4,685

Type	# variants (BA)	# variants (BB)	# genes (BA)	# genes (BB)	# genes (BA unique)	# genes (BB unique)	# genes (BA & BB)
**SMV**	3,870,992	356,457	34,415	27,383	7,032	383	27,383
**SV**	87,250	36,591	34,495	13,397	27,581	6,483	6,914

The following GO analysis result tables are subsets based on keywords (e.g., *morphogenesis*, *growth*, *embryonic*, *development*, and *formation*); the complete tables for the listed approaches - for **biological process** (**BP**), **molecular function** (**MF**) and **cellular component** (**CC**) terms - are available on the data repository. An overview of GO term profile sizes and subset sizes is given in Tab. S14b.

Table S14b Overview on analysis approaches and resulting numbers of GO terms. This table shows the number of GO terms in the respective biological process terms result tables - i.e., molecular function and cellular component terms are omitted. The marked entries are shown below in Tab. S15 and Tab. S18. Variant effect-based approaches with variant weighting are omitted.

Approach	Description	#GO Terms	#GO Terms in subset
A1	gene categories based on variant comparison groups; variant counts (mutation load) as measure; no ranking and quantile threshold	188	65
**A2**	gene categories based on variant comparison groups; variant counts (mutation load) as measure; with ranking and quantile threshold of p=0.5 (median)	246	78
A3	gene categories based on variant comparison groups; variant counts (mutation load) as measure; with ranking and quantile threshold of p=0.75 (highest quartile)	129	34
A4	gene categories based on variant comparison groups; variant counts (mutation load) as measure; counts normalized on gene length; with ranking and quantile threshold of p=0.5 (median)	314	75
A5	gene categories based on variant comparison groups; variant counts (mutation load) as measure; counts normalized on gene length; with ranking and quantile threshold of p=0.75 (highest quartile)	254	51
**T1**	gene categories based on presence in target list	81	17

For comparison, the dataset contains 7,905 (PT) and 7,688 (TM) GO terms in total, the respective dataset of *D. rerio* (Zebrafish) contains 10,006 GO terms in total (http://geneontology.org, release 2020-07-16), and the current general GO database statistic (http://geneontology.org, release 2020-07-16) lists:

- Biological process terms 28923
- Molecular function terms 11136
- Cellular component terms 4185

Table S15 GO enrichment analysis result – biological process terms. This table shows results from approach **A2** (see Tab. S14b). Enrichment was assessed via a Fisher's exact test with a cutoff of p<=0.001 and GO topology was accounted for (R package topGO, method *weight*). In the **Type** column biological species are coded as A and B; identical and nonidentical variants at same nucleotide positions, and unique variants are indicated. The categories (AA, AB, BA and BB) refer to the within and between comparison; identical (AA) means that the intraspecific variants (SMVs and SVs) in this group have also been called in the related interspecific (AB) comparison at the same location, with nonidentical (AA) a different variant has been called at the same location (e.g., A→T within and A→G between species), with unique(AA) only within species A and with unique(AB) only between species A and B a variant was called at that position; the same holds for species B and the BB and BA categories. Comparisons have been conducted two-way, i.e., A vs B and B vs A; the groups were tested against a gene universe containing all genes with GO information. SMV: small variant/s (SNP and InDel); SV: structural variant/s (insertion, deletion, duplication, inversion and translocation).

Type	GO ID	Description	p-value
**SMV:identical (AA)**	GO:0030513	positive regulation of BMP signaling pathway	2.80E-08
**SMV:identical (AA)**	GO:0016048	detection of temperature stimulus	3.40E-14
**SMV:identical (AA)**	GO:0050974	detection of mechanical stimulus involved in sensory perception	2.70E-05
**SMV:identical (AA)**	GO:0050976	detection of mechanical stimulus involved in sensory perception of touch	6.50E-04
**SMV:identical (AA)**	GO:0021772	olfactory bulb development	2.70E-06
**SMV:identical (AA)**	GO:0001822	kidney development	4.30E-04
**SMV:identical (AA)**	GO:0007399	nervous system development	7.30E-04
**SMV:identical (AA)**	GO:0048703	**embryonic viscerocranium morphogenesis**	3.00E-06
**SMV:identical (AA)**	GO:0033334	fin morphogenesis	1.20E-05
**SMV:identical (AA)**	GO:0001841	neural tube formation	2.30E-08
**SMV:identical (AA)**	GO:0001578	microtubule bundle formation	2.70E-05
**SMV:identical (AA)**	GO:0038084	vascular endothelial growth factor signaling pathway	7.50E-07
**SMV:identical (AA)**	GO:0048009	insulin-like growth factor receptor signaling pathway	4.10E-05
**SMV:identical (AA)**	GO:0035775	pronephric glomerulus morphogenesis	4.90E-04
**SMV:identical (AB)**	GO:0030513	positive regulation of BMP signaling pathway	1.50E-09
**SMV:identical (AB)**	GO:0030509	BMP signaling pathway	3.20E-09
**SMV:identical (AB)**	GO:0016048	detection of temperature stimulus	1.90E-11
**SMV:identical (AB)**	GO:0050974	detection of mechanical stimulus involved in sensory perception	2.40E-04
**SMV:identical (AB)**	GO:0021915	neural tube development	2.00E-07

Type	GO ID	Description	p-value
**SMV:identical (AB)**	GO:0031175	neuron projection development	1.70E-04
**SMV:identical (AB)**	GO:0007508	larval heart development	5.20E-04
**SMV:identical (AB)**	GO:0001822	kidney development	6.70E-04
**SMV:identical (AB)**	GO:0021772	olfactory bulb development	8.40E-04
**SMV:identical (AB)**	GO:0051764	actin crosslink formation	1.30E-09
**SMV:identical (AB)**	GO:0001841	neural tube formation	8.60E-07
**SMV:identical (AB)**	GO:0060972	left/right pattern formation	1.40E-04
**SMV:identical (AB)**	GO:0010842	retina layer formation	5.80E-04
**SMV:identical (AB)**	GO:0038084	vascular endothelial growth factor signaling pathway	2.40E-05
**SMV:identical (AB)**	GO:0022604	regulation of cell morphogenesis	6.20E-05
**SMV:identical (BA)**	GO:0060536	cartilage morphogenesis	3.80E-05
**SMV:identical (BA)**	GO:0001502	cartilage condensation	2.10E-04
**SMV:identical (BA)**	GO:0016048	detection of temperature stimulus	2.60E-05
**SMV:identical (BA)**	GO:0048747	muscle fiber development	1.30E-08
**SMV:identical (BA)**	GO:0033333	fin development	8.00E-05
**SMV:identical (BA)**	GO:0001654	eye development	9.70E-05
**SMV:identical (BA)**	GO:0021915	neural tube development	1.20E-04
**SMV:identical (BA)**	GO:0001822	kidney development	4.50E-04
**SMV:identical (BA)**	GO:0021591	ventricular system development	5.50E-04
**SMV:identical (BA)**	GO:0007517	muscle organ development	5.70E-04
**SMV:identical (BA)**	GO:0048703	**embryonic viscerocranium morphogenesis**	1.30E-08
**SMV:identical (BA)**	GO:0048048	embryonic eye morphogenesis	6.00E-07
**SMV:identical (BA)**	GO:0035166	post-embryonic hemopoiesis	1.20E-05
**SMV:identical (BA)**	GO:0033334	fin morphogenesis	4.30E-06
**SMV:identical (BA)**	GO:0035142	dorsal fin morphogenesis	9.10E-04
**SMV:identical (BA)**	GO:0060347	heart trabecula formation	8.20E-07
**SMV:identical (BA)**	GO:0038084	vascular endothelial growth factor signaling pathway	4.80E-08
**SMV:identical (BA)**	GO:0030947	regulation of vascular endothelial growth factor receptor signaling pathway	2.90E-06
**SMV:identical (BA)**	GO:0040014	regulation of multicellular organism growth	2.10E-04
**SMV:identical (BA)**	GO:0045887	positive regulation of synaptic growth at neuromuscular junction	9.10E-04
**SMV:identical (BA)**	GO:0045197	establishment or maintenance of epithelial cell apical/basal polarity	4.30E-08
**SMV:identical (BA)**	GO:0061001	regulation of dendritic spine morphogenesis	3.80E-10
**SMV:identical (BA)**	GO:0048814	regulation of dendrite morphogenesis	7.90E-04
**SMV:identical (BA)**	GO:0014814	axon regeneration at neuromuscular junction	9.10E-04
**SMV:identical (BA)**	GO:0048680	positive regulation of axon regeneration	9.10E-04
**SMV:identical (BB)**	GO:0016048	detection of temperature stimulus	3.40E-06
**SMV:identical (BB)**	GO:0033333	fin development	4.90E-05
**SMV:identical (BB)**	GO:0042478	regulation of eye photoreceptor cell development	6.90E-04
**SMV:identical (BB)**	GO:0048703	**embryonic viscerocranium morphogenesis**	8.20E-10
**SMV:identical (BB)**	GO:0048048	embryonic eye morphogenesis	1.20E-09
**SMV:identical (BB)**	GO:0035162	embryonic hemopoiesis	1.50E-08
**SMV:identical (BB)**	GO:0035166	post-embryonic hemopoiesis	1.00E-06
**SMV:identical (BB)**	GO:0033334	fin morphogenesis	7.70E-06
**SMV:identical (BB)**	GO:0035142	dorsal fin morphogenesis	3.10E-04
**SMV:identical (BB)**	GO:0038084	vascular endothelial growth factor signaling pathway	2.10E-09
**SMV:identical (BB)**	GO:0030947	regulation of vascular endothelial growth factor receptor signaling pathway	2.70E-06
**SMV:identical (BB)**	GO:0040014	regulation of multicellular organism growth	2.90E-05
**SMV:identical (BB)**	GO:0045197	establishment or maintenance of epithelial cell apical/basal polarity	3.00E-11
**SMV:identical (BB)**	GO:0061001	regulation of dendritic spine morphogenesis	1.60E-10
**SMV:nonidentical (AA)**	GO:0030513	positive regulation of BMP signaling pathway	2.30E-08
**SMV:nonidentical (AA)**	GO:0016048	detection of temperature stimulus	2.70E-14
**SMV:nonidentical (AA)**	GO:0050976	detection of mechanical stimulus involved in sensory perception of touch	6.10E-04
**SMV:nonidentical (AA)**	GO:0050974	detection of mechanical stimulus involved in sensory perception	9.90E-04
**SMV:nonidentical (AA)**	GO:0031175	neuron projection development	7.30E-06
**SMV:nonidentical (AA)**	GO:0007399	nervous system development	1.00E-05
**SMV:nonidentical (AA)**	GO:0001501	skeletal system development	7.50E-04
**SMV:nonidentical (AA)**	GO:0048703	**embryonic viscerocranium morphogenesis**	2.30E-06
**SMV:nonidentical (AA)**	GO:0001841	neural tube formation	1.40E-09
**SMV:nonidentical (AA)**	GO:0038084	vascular endothelial growth factor signaling pathway	1.50E-05
**SMV:nonidentical (AA)**	GO:0048009	insulin-like growth factor receptor signaling pathway	3.70E-05
**SMV:nonidentical (AA)**	GO:0022604	regulation of cell morphogenesis	8.80E-07
**SMV:nonidentical (AA)**	GO:0035775	pronephric glomerulus morphogenesis	4.30E-04
**SMV:nonidentical (AB)**	GO:0030509	BMP signaling pathway	6.20E-11
**SMV:nonidentical (AB)**	GO:0030513	positive regulation of BMP signaling pathway	6.60E-09
**SMV:nonidentical (AB)**	GO:0016048	detection of temperature stimulus	1.70E-13
**SMV:nonidentical (AB)**	GO:0021915	neural tube development	5.50E-09
**SMV:nonidentical (AB)**	GO:0031175	neuron projection development	2.80E-05
**SMV:nonidentical (AB)**	GO:0007399	nervous system development	1.40E-04
**SMV:nonidentical (AB)**	GO:0043010	camera-type eye development	1.60E-04
**SMV:nonidentical (AB)**	GO:0021549	cerebellum development	6.60E-04
**SMV:nonidentical (AB)**	GO:0051764	actin crosslink formation	1.70E-11
**SMV:nonidentical (AB)**	GO:0001841	neural tube formation	9.90E-08
**SMV:nonidentical (AB)**	GO:0060972	left/right pattern formation	5.40E-06
**SMV:nonidentical (AB)**	GO:0010842	retina layer formation	7.40E-05

Type	GO ID	Description	p-value
**SMV:nonidentical (AB)**	GO:0038084	vascular endothelial growth factor signaling pathway	3.60E-05
**SMV:nonidentical (AB)**	GO:0022604	regulation of cell morphogenesis	3.10E-05
**SMV:nonidentical (BA)**	GO:0001502	cartilage condensation	3.40E-09
**SMV:nonidentical (BA)**	GO:0060536	cartilage morphogenesis	2.40E-04
**SMV:nonidentical (BA)**	GO:0016048	detection of temperature stimulus	4.40E-06
**SMV:nonidentical (BA)**	GO:0021915	neural tube development	1.40E-06
**SMV:nonidentical (BA)**	GO:0007275	multicellular organism development	1.70E-05
**SMV:nonidentical (BA)**	GO:0033333	fin development	6.50E-05
**SMV:nonidentical (BA)**	GO:0043010	camera-type eye development	1.40E-04
**SMV:nonidentical (BA)**	GO:0021591	ventricular system development	1.50E-04
**SMV:nonidentical (BA)**	GO:0001822	kidney development	3.10E-04
**SMV:nonidentical (BA)**	GO:0042478	regulation of eye photoreceptor cell development	8.10E-04
**SMV:nonidentical (BA)**	GO:0048703	**embryonic viscerocranium morphogenesis**	5.90E-13
**SMV:nonidentical (BA)**	GO:0048048	embryonic eye morphogenesis	1.90E-09
**SMV:nonidentical (BA)**	GO:0035166	post-embryonic hemopoiesis	1.40E-06
**SMV:nonidentical (BA)**	GO:0010172	embryonic body morphogenesis	1.40E-05
**SMV:nonidentical (BA)**	GO:0033334	fin morphogenesis	1.60E-06
**SMV:nonidentical (BA)**	GO:0035142	dorsal fin morphogenesis	3.60E-04
**SMV:nonidentical (BA)**	GO:0038084	vascular endothelial growth factor signaling pathway	7.90E-08
**SMV:nonidentical (BA)**	GO:0030947	regulation of vascular endothelial growth factor receptor signaling pathway	2.60E-07
**SMV:nonidentical (BA)**	GO:0040014	regulation of multicellular organism growth	3.70E-05
**SMV:nonidentical (BA)**	GO:0045197	establishment or maintenance of epithelial cell apical/basal polarity	4.40E-11
**SMV:nonidentical (BA)**	GO:0061001	regulation of dendritic spine morphogenesis	2.60E-10
**SMV:nonidentical (BB)**	GO:0030500	regulation of bone mineralization	5.70E-06
**SMV:nonidentical (BB)**	GO:0016048	detection of temperature stimulus	1.50E-06
**SMV:nonidentical (BB)**	GO:0021591	ventricular system development	4.20E-08
**SMV:nonidentical (BB)**	GO:0072015	glomerular visceral epithelial cell development	6.10E-08
**SMV:nonidentical (BB)**	GO:0033333	fin development	1.90E-05
**SMV:nonidentical (BB)**	GO:0048747	muscle fiber development	8.10E-05
**SMV:nonidentical (BB)**	GO:0042478	regulation of eye photoreceptor cell development	4.20E-04
**SMV:nonidentical (BB)**	GO:0048048	embryonic eye morphogenesis	2.20E-10
**SMV:nonidentical (BB)**	GO:0048703	**embryonic viscerocranium morphogenesis**	1.20E-09
**SMV:nonidentical (BB)**	GO:0035162	embryonic hemopoiesis	2.30E-09
**SMV:nonidentical (BB)**	GO:0035166	post-embryonic hemopoiesis	3.80E-07
**SMV:nonidentical (BB)**	GO:0033334	fin morphogenesis	2.30E-06
**SMV:nonidentical (BB)**	GO:0035142	dorsal fin morphogenesis	2.10E-04
**SMV:nonidentical (BB)**	GO:0038084	vascular endothelial growth factor signaling pathway	1.80E-08
**SMV:nonidentical (BB)**	GO:0030947	regulation of vascular endothelial growth factor receptor signaling pathway	1.00E-06
**SMV:nonidentical (BB)**	GO:0040014	regulation of multicellular organism growth	1.30E-05
**SMV:nonidentical (BB)**	GO:0045197	establishment or maintenance of epithelial cell apical/basal polarity	8.40E-12
**SMV:nonidentical (BB)**	GO:0061001	regulation of dendritic spine morphogenesis	3.50E-11
**SMV:unique (AA)**	GO:0030513	positive regulation of BMP signaling pathway	1.60E-07
**SMV:unique (AA)**	GO:0016048	detection of temperature stimulus	2.90E-13
**SMV:unique (AA)**	GO:0050974	detection of mechanical stimulus involved in sensory perception	1.00E-04
**SMV:unique (AA)**	GO:0021772	olfactory bulb development	6.80E-07
**SMV:unique (AA)**	GO:0007399	nervous system development	3.30E-04
**SMV:unique (AA)**	GO:0048703	**embryonic viscerocranium morphogenesis**	3.50E-05
**SMV:unique (AA)**	GO:0033334	fin morphogenesis	4.40E-05
**SMV:unique (AA)**	GO:0001841	neural tube formation	1.50E-08
**SMV:unique (AA)**	GO:0003262	endocardial progenitor cell migration to the midline involved in heart field formation	3.40E-05
**SMV:unique (AA)**	GO:0001578	microtubule bundle formation	1.00E-04
**SMV:unique (AA)**	GO:0038084	vascular endothelial growth factor signaling pathway	2.40E-06
**SMV:unique (AA)**	GO:0048009	insulin-like growth factor receptor signaling pathway	1.10E-04
**SMV:unique (AA)**	GO:0001763	morphogenesis of a branching structure	1.80E-04
**SMV:unique (AB)**	GO:0030513	positive regulation of BMP signaling pathway	2.40E-08
**SMV:unique (AB)**	GO:0030509	BMP signaling pathway	1.50E-07
**SMV:unique (AB)**	GO:0016048	detection of temperature stimulus	1.60E-11
**SMV:unique (AB)**	GO:0048747	muscle fiber development	1.60E-06
**SMV:unique (AB)**	GO:0001822	kidney development	1.20E-04
**SMV:unique (AB)**	GO:0031175	neuron projection development	1.50E-04
**SMV:unique (AB)**	GO:0051764	actin crosslink formation	1.10E-09
**SMV:unique (AB)**	GO:0001841	neural tube formation	7.20E-07
**SMV:unique (AB)**	GO:0010842	retina layer formation	5.00E-04
**SMV:unique (AB)**	GO:0038084	vascular endothelial growth factor signaling pathway	2.20E-05
**SMV:unique (AB)**	GO:0022604	regulation of cell morphogenesis	5.60E-05
**SMV:unique (BA)**	GO:0060536	cartilage morphogenesis	2.60E-05
**SMV:unique (BA)**	GO:0001502	cartilage condensation	1.70E-04
**SMV:unique (BA)**	GO:0016048	detection of temperature stimulus	2.10E-05
**SMV:unique (BA)**	GO:0033333	fin development	6.00E-05
**SMV:unique (BA)**	GO:0007275	multicellular organism development	1.60E-04
**SMV:unique (BA)**	GO:0001822	kidney development	3.50E-04
**SMV:unique (BA)**	GO:0021591	ventricular system development	4.70E-04
**SMV:unique (BA)**	GO:0048048	embryonic eye morphogenesis	4.00E-07
**SMV:unique (BA)**	GO:0048703	**embryonic viscerocranium morphogenesis**	1.00E-06

Type	GO ID	Description	p-value
**SMV:unique (BA)**	GO:0035166	post-embryonic hemopoiesis	9.50E-06
**SMV:unique (BA)**	GO:0033334	fin morphogenesis	3.90E-10
**SMV:unique (BA)**	GO:0035142	dorsal fin morphogenesis	8.10E-04
**SMV:unique (BA)**	GO:0060347	heart trabecula formation	6.50E-07
**SMV:unique (BA)**	GO:0038084	vascular endothelial growth factor signaling pathway	3.40E-08
**SMV:unique (BA)**	GO:0030947	regulation of vascular endothelial growth factor receptor signaling pathway	2.20E-06
**SMV:unique (BA)**	GO:0040014	regulation of multicellular organism growth	1.70E-04
**SMV:unique (BA)**	GO:0045887	positive regulation of synaptic growth at neuromuscular junction	8.10E-04
**SMV:unique (BA)**	GO:0045197	establishment or maintenance of epithelial cell apical/basal polarity	3.10E-08
**SMV:unique (BA)**	GO:0061001	regulation of dendritic spine morphogenesis	2.40E-10
**SMV:unique (BA)**	GO:0014814	axon regeneration at neuromuscular junction	8.10E-04
**SMV:unique (BA)**	GO:0048680	positive regulation of axon regeneration	8.10E-04
**SMV:unique (BB)**	GO:0016048	detection of temperature stimulus	4.20E-06
**SMV:unique (BB)**	GO:0021591	ventricular system development	6.30E-06
**SMV:unique (BB)**	GO:0048747	muscle fiber development	2.40E-05
**SMV:unique (BB)**	GO:0033333	fin development	6.30E-05
**SMV:unique (BB)**	GO:0042478	regulation of eye photoreceptor cell development	7.90E-04
**SMV:unique (BB)**	GO:0048703	**embryonic viscerocranium morphogenesis**	1.70E-09
**SMV:unique (BB)**	GO:0048048	embryonic eye morphogenesis	1.80E-09
**SMV:unique (BB)**	GO:0035162	embryonic hemopoiesis	2.40E-08
**SMV:unique (BB)**	GO:0035166	post-embryonic hemopoiesis	1.30E-06
**SMV:unique (BB)**	GO:0033334	fin morphogenesis	1.00E-05
**SMV:unique (BB)**	GO:0035142	dorsal fin morphogenesis	3.50E-04
**SMV:unique (BB)**	GO:0038084	vascular endothelial growth factor signaling pathway	2.90E-09
**SMV:unique (BB)**	GO:0030947	regulation of vascular endothelial growth factor receptor signaling pathway	3.50E-06
**SMV:unique (BB)**	GO:0040014	regulation of multicellular organism growth	3.60E-05
**SMV:unique (BB)**	GO:0045197	establishment or maintenance of epithelial cell apical/basal polarity	4.10E-11
**SMV:unique (BB)**	GO:0061001	regulation of dendritic spine morphogenesis	2.40E-10

Type	GO Term	Description	p-value
**SV:identical (AA)**	GO:0048703	**embryonic viscerocranium morphogenesis**	4.40E-11
**SV:identical (AA)**	GO:0035775	pronephric glomerulus morphogenesis	5.10E-11
**SV:identical (AA)**	GO:0072078	nephron tubule morphogenesis	3.30E-10
**SV:identical (AB)**	GO:0021954	central nervous system neuron development	5.20E-08
**SV:identical (AB)**	GO:0048703	**embryonic viscerocranium morphogenesis**	4.00E-13
**SV:identical (AB)**	GO:0001578	microtubule bundle formation	9.00E-09
**SV:identical (AB)**	GO:0035775	pronephric glomerulus morphogenesis	1.70E-15
**SV:identical (AB)**	GO:0072078	nephron tubule morphogenesis	2.40E-14
**SV:identical (BA)**	GO:0030510	regulation of BMP signaling pathway	2.30E-06
**SV:identical (BA)**	GO:0048048	embryonic eye morphogenesis	8.30E-07
**SV:identical (BA)**	GO:0048703	**embryonic viscerocranium morphogenesis**	2.60E-05
**SV:identical (BA)**	GO:0009953	dorsal/ventral pattern formation	2.00E-05
**SV:identical (BA)**	GO:0045197	establishment or maintenance of epithelial cell apical/basal polarity	9.20E-09
**SV:identical (BA)**	GO:0061001	regulation of dendritic spine morphogenesis	5.40E-09
**SV:identical (BA)**	GO:0035775	pronephric glomerulus morphogenesis	2.60E-06
**SV:identical (BA)**	GO:0048514	blood vessel morphogenesis	1.10E-05
**SV:identical (BA)**	GO:0072078	nephron tubule morphogenesis	1.70E-05
**SV:identical (BB)**	GO:0030510	regulation of BMP signaling pathway	7.30E-07
**SV:identical (BB)**	GO:0048703	**embryonic viscerocranium morphogenesis**	7.90E-06
**SV:identical (BB)**	GO:0009953	dorsal/ventral pattern formation	6.10E-06
**SV:identical (BB)**	GO:0035775	pronephric glomerulus morphogenesis	1.30E-06
**SV:identical (BB)**	GO:0072078	nephron tubule morphogenesis	8.50E-06
**SV:identical (BB)**	GO:0048514	blood vessel morphogenesis	1.00E-04
**SV:nonidentical (AA)**	GO:0060041	retina development in camera-type eye	1.10E-05
**SV:nonidentical (AA)**	GO:0031101	fin regeneration	7.20E-06
**SV:nonidentical (AB)**	GO:0060041	retina development in camera-type eye	8.10E-05
**SV:nonidentical (AB)**	GO:0048917	posterior lateral line ganglion development	8.30E-04
**SV:nonidentical (BA)**	GO:0072015	glomerular visceral epithelial cell development	2.10E-16
**SV:nonidentical (BA)**	GO:0007420	brain development	3.80E-06
**SV:nonidentical (BB)**	GO:0072015	glomerular visceral epithelial cell development	3.00E-19
**SV:nonidentical (BB)**	GO:0001889	liver development	1.10E-14
**SV:unique (AA)**	GO:0048703	**embryonic viscerocranium morphogenesis**	5.40E-06
**SV:unique (AA)**	GO:0035775	pronephric glomerulus morphogenesis	9.80E-08
**SV:unique (AA)**	GO:0072078	nephron tubule morphogenesis	5.70E-07
**SV:unique (AB)**	GO:0016048	detection of temperature stimulus	7.00E-13
**SV:unique (AB)**	GO:0050974	detection of mechanical stimulus involved in sensory perception	9.70E-04
**SV:unique (AB)**	GO:0048747	muscle fiber development	5.90E-05
**SV:unique (AB)**	GO:0048703	**embryonic viscerocranium morphogenesis**	8.80E-06
**SV:unique (AB)**	GO:0001578	microtubule bundle formation	1.80E-04
**SV:unique (AB)**	GO:0038084	vascular endothelial growth factor signaling pathway	4.00E-06

Type	GO Term	Description	p-value
**SV:unique (AB)**	GO:0035775	pronephric glomerulus morphogenesis	2.60E-05
**SV:unique (AB)**	GO:0072078	nephron tubule morphogenesis	2.10E-04
**SV:unique (BA)**	GO:0030510	regulation of BMP signaling pathway	9.20E-07
**SV:unique (BA)**	GO:0030500	regulation of bone mineralization	4.40E-06
**SV:unique (BA)**	GO:0009584	detection of visible light	1.00E-11
**SV:unique (BA)**	GO:0046549	retinal cone cell development	4.10E-11
**SV:unique (BA)**	GO:0045682	regulation of epidermis development	3.60E-10
**SV:unique (BA)**	GO:0048919	posterior lateral line neuromast development	3.70E-06
**SV:unique (BA)**	GO:0072015	glomerular visceral epithelial cell development	1.10E-05
**SV:unique (BA)**	GO:0001824	blastocyst development	9.00E-05
**SV:unique (BA)**	GO:0007417	central nervous system development	3.80E-04
**SV:unique (BA)**	GO:0061008	hepaticobiliary system development	5.80E-04
**SV:unique (BA)**	GO:0035162	embryonic hemopoiesis	2.20E-07
**SV:unique (BA)**	GO:0016332	establishment or maintenance of polarity of embryonic epithelium	2.00E-05
**SV:unique (BA)**	GO:0001841	neural tube formation	1.10E-04
**SV:unique (BA)**	GO:0042138	meiotic DNA double-strand break formation	7.70E-04
**SV:unique (BA)**	GO:0038084	vascular endothelial growth factor signaling pathway	4.40E-06
**SV:unique (BA)**	GO:0030947	regulation of vascular endothelial growth factor receptor signaling pathway	3.80E-04
**SV:unique (BA)**	GO:0045197	establishment or maintenance of epithelial cell apical/basal polarity	6.10E-06
**SV:unique (BA)**	GO:0045199	maintenance of epithelial cell apical/basal polarity	2.10E-04
**SV:unique (BA)**	GO:0048514	blood vessel morphogenesis	3.00E-13
**SV:unique (BA)**	GO:0010771	negative regulation of cell morphogenesis involved in differentiation	3.60E-10
**SV:unique (BA)**	GO:0061001	regulation of dendritic spine morphogenesis	1.50E-09
**SV:unique (BA)**	GO:0061072	iris morphogenesis	3.40E-07
**SV:unique (BB)**	GO:0045682	regulation of epidermis development	5.30E-15
**SV:unique (BB)**	GO:0048919	posterior lateral line neuromast development	1.10E-10
**SV:unique (BB)**	GO:0072015	glomerular visceral epithelial cell development	3.20E-09
**SV:unique (BB)**	GO:0048747	muscle fiber development	4.50E-09
**SV:unique (BB)**	GO:0021744	dorsal motor nucleus of vagus nerve development	1.10E-06
**SV:unique (BB)**	GO:0007275	multicellular organism development	3.10E-04
**SV:unique (BB)**	GO:0043588	skin development	5.70E-04
**SV:unique (BB)**	GO:0016332	establishment or maintenance of polarity of embryonic epithelium	1.30E-08
**SV:unique (BB)**	GO:0035162	embryonic hemopoiesis	4.60E-06
**SV:unique (BB)**	GO:0001841	neural tube formation	4.80E-05
**SV:unique (BB)**	GO:0038084	vascular endothelial growth factor signaling pathway	3.30E-08
**SV:unique (BB)**	GO:0030947	regulation of vascular endothelial growth factor receptor signaling pathway	3.30E-07
**SV:unique (BB)**	GO:0040014	regulation of multicellular organism growth	3.10E-06
**SV:unique (BB)**	GO:0045199	maintenance of epithelial cell apical/basal polarity	1.70E-07
**SV:unique (BB)**	GO:0048514	blood vessel morphogenesis	5.60E-16
**SV:unique (BB)**	GO:0010771	negative regulation of cell morphogenesis involved in differentiation	1.90E-09
**SV:unique (BB)**	GO:0055008	cardiac muscle tissue morphogenesis	3.20E-06
**SV:unique (BB)**	GO:0002011	morphogenesis of an epithelial sheet	7.70E-06

Table S16 Intersection GO term set. This table shows the intersection of all approaches (without gene length normalization); however, *embryonic viscerocranium morphogenesis* (and *eye morphogenesis*) also remain in the intersections including approaches with length normalization (not shown). The intersection has been applied to the full GO term profiles, i.e., not to the keyword-based subsets; hence, very basic processes are listed.

GO ID	Description
**GO:0001578**	microtubule bundle formation
**GO:0001841**	neural tube formation
**GO:0002724**	regulation of T cell cytokine production
**GO:0006171**	cAMP biosynthetic process
**GO:0006307**	DNA dealkylation involved in DNA repair
**GO:0006468**	protein phosphorylation
**GO:0006470**	protein dephosphorylation
**GO:0006606**	protein import into nucleus
**GO:0006811**	ion transport
**GO:0006812**	cation transport
**GO:0006874**	cellular calcium ion homeostasis
**GO:0006897**	endocytosis
**GO:0007155**	cell adhesion
**GO:0007156**	homophilic cell adhesion via plasma membrane adhesion molecules
**GO:0007165**	signal transduction
**GO:0007169**	transmembrane receptor protein tyrosine kinase signaling pathway
**GO:0007205**	protein kinase C-activating G-protein coupled receptor signaling pathway
**GO:0007264**	small GTPase mediated signal transduction
**GO:0007268**	chemical synaptic transmission
**GO:0007269**	neurotransmitter secretion

GO ID	Description
GO:0007275	multicellular organism development
GO:0007405	neuroblast proliferation
GO:0007411	axon guidance
GO:0007413	axonal fasciculation
GO:0007626	locomotory behavior
GO:0010735	positive regulation of transcription via serum response element binding
GO:0015701	bicarbonate transport
GO:0016048	detection of temperature stimulus
GO:0016199	axon midline choice point recognition
GO:0021535	cell migration in hindbrain
GO:0030036	actin cytoskeleton organization
GO:0030042	actin filament depolymerization
GO:0030317	flagellated sperm motility
GO:0030334	regulation of cell migration
GO:0030509	BMP signaling pathway
GO:0030513	positive regulation of BMP signaling pathway
GO:0030947	regulation of vascular endothelial growth factor receptor signaling pathway
GO:0031290	retinal ganglion cell axon guidance
GO:0032012	regulation of ARF protein signal transduction
GO:0035023	regulation of Rho protein signal transduction
GO:0035315	hair cell differentiation
GO:0035552	oxidative single-stranded DNA demethylation
GO:0035553	oxidative single-stranded RNA demethylation
GO:0035556	intracellular signal transduction
GO:0038084	vascular endothelial growth factor signaling pathway
GO:0040014	regulation of multicellular organism growth
GO:0042245	RNA repair
GO:0043010	**camera-type eye development**
GO:0043547	positive regulation of GTPase activity
GO:0045197	establishment or maintenance of epithelial cell apical/basal polarity
GO:0046856	phosphatidylinositol dephosphorylation
GO:0048048	**embryonic eye morphogenesis**
GO:0048703	**embryonic viscerocranium morphogenesis**
GO:0048791	calcium ion-regulated exocytosis of neurotransmitter
GO:0050804	modulation of chemical synaptic transmission
GO:0050808	synapse organization
GO:0050896	response to stimulus
GO:0050951	sensory perception of temperature stimulus
GO:0051056	regulation of small GTPase mediated signal transduction
GO:0051262	protein tetramerization
GO:0051660	establishment of centrosome localization
GO:0051969	regulation of transmission of nerve impulse
GO:0055072	iron ion homeostasis
GO:0060030	dorsal convergence
GO:0061001	regulation of dendritic spine morphogenesis
GO:0061337	cardiac conduction
GO:0070509	calcium ion import
GO:0070588	calcium ion transmembrane transport
GO:0097264	self proteolysis

So far, genes in the GO group *pharyngeal system development* and target genes collected from literature (especially from the cited studies on facial shapes in humans; see Tab. S17) have been further analyzed in more detail. The approach **T1** (see Tab. S14b) is just a convenient way to retrieve the variant carrying genes belonging to the GO terms of interest.

Table S17 Target gene set. The genes in this list were selected in a literature research; they include known morphogenes, in part, with relevance to the pharyngeal system.

ACAD9	CTNNBL1	HOXB6B	PITX2-LIKE	SRL
ALAS1	CXCR5	HOXD	PKDCC	SUPT3H
ALAS2	DCHS2	HSPA8	PKP1	SWS1
ALOXE3-LIKE	DES-LIKE	HSPB1	PTCH1	SWS2A
ALPL	DLC	HSPB8	PTGS2	SWS2B
ANXA6-LIKE	DLX2	IER2	RAB7A	SYT2
ASPM	DLX2A	IL20RA	RARG	TBX15
BARX1	DLX6	IL22RA1	RGS2	TGF
BARX1-LIKE	DVL3	KCNJ11	RH1	THBS3
BC039327	DYNC1L1	KCTD15	RH2A-ALPHA	TNFSF11
BMP16	EDA-LIKE	KIR7	RH2A-BETA	TNNC1

BMP2	EDAR	KITA	RH2B	TNNC1B
BMP2B	EPHB3	KITLA	RPS12	TNNC2
BMP4	EVE1-LIKE	KITLGA	RUNX1-LIKE	TNNI
BMPK2	EYA4	KLF4	RUNX2	TNNI1
BMPR1B	FAT3	LHX6-LIKE	RUNX2B	TNNI1AL
C1QL2	FBXO36B	LHX8	RUNX2B-LIKE	TNNI2
C1QL4	FHL1	LMOD2	RUNX3-LIKE	TNNI-LIKE
C1Q-LIKE	FHL2	LWS	RX1	TNNT1
CACNG6	FHL5	MITFA	RXRA	TNNT1-1-LIKE
CCR7-LIKE	FNDC1	MYBPH-LIKE	RYR1	TNNT3
CDH18	FOS	NHP2	RYR1-LIKE	TPM1(ALPHA)
CFOS	GJA1	NOG	SEMA3C	TPM2
C-FOS	GLI3	NOG2-LIKE	SHH	TPM4
COL12A1	GPR126-LIKE	NOS1	SLMAP	TPMA
COL6A1	HAG	OSX	SMTLB	USP28
COL6A3	HAGOROMO	PAX1	SOX10	WNT
CREB1	HAPLN1	PAX3	SOX9	WNT5A
CRFB1	HAPLN1A	PAX9	SP7	WNT7B
CRFB4	HOX	PDLIM3-LIKE	SPON2A-LIKE	ZNF354A
CSF1RA	HOXB5A-LIKE	PITX2	SPON2-LIKE	

Table S18 GO analysis result for targeted approach – biological process terms. This table shows results from approach **T1** (see Tab. S14b). Enrichment was assessed via a Fisher's exact test with a cutoff of p<=0.001 and GO topology was accounted for (R package topGO, method *weight*). In the **Type** column biological species are coded as A and B; identical and nonidentical variants at same nucleotide positions, and unique variants are indicated. The categories (AA, AB, BA and BB) refer to the within and between comparison; identical (AA) means that the intraspecific variants (SMVs and SVs) in this group have also been called in the related interspecific (AB) comparison at the same location, with nonidentical (AA) a different variant has been called at the same location (e.g., A→T within and A→G between species), with unique(AA) only within species A and with unique(AB) only between species A and B a variant was called at that position; the same holds for species B and the BB and BA categories. Comparisons have been conducted two-way, i.e., A vs B and B vs A; the groups were tested against a gene universe containing all genes with GO information. SMV: small variant/s (SNPs and InDels); SV: structural variant/s (insertions, deletions, duplications, inversions and translocations).

Type	GO Term	Description	p-value
**SMV:identical (AA)**	GO:0072358	cardiovascular system development	1.30E-04
**SMV:identical (AA)**	GO:0060037	**pharyngeal system development**	2.70E-04
**SMV:identical (AA)**	GO:0043282	**pharyngeal muscle development**	5.80E-04
**SMV:identical (AA)**	GO:0060023	**soft palate development**	5.80E-04
**SMV:identical (AA)**	GO:0060017	parathyroid gland development	5.80E-04
**SMV:identical (AB)**	GO:0072358	cardiovascular system development	1.30E-04
**SMV:identical (AB)**	GO:0060037	**pharyngeal system development**	2.70E-04
**SMV:identical (AB)**	GO:0043282	**pharyngeal muscle development**	5.80E-04
**SMV:identical (AB)**	GO:0060023	**soft palate development**	5.80E-04
**SMV:identical (AB)**	GO:0060017	parathyroid gland development	5.80E-04
**SMV:identical (BA)**	GO:0060037	**pharyngeal system development**	2.50E-07
**SMV:identical (BB)**	GO:0060037	**pharyngeal system development**	2.50E-07
**SMV:nonidentical (AA)**	GO:0007420	brain development	5.10E-04
**SMV:nonidentical (AB)**	GO:0007420	brain development	5.10E-04
**SMV:unique (AA)**	GO:0060037	**pharyngeal system development**	1.20E-05
**SMV:unique (AA)**	GO:0036342	post-anal tail morphogenesis	7.90E-04
**SMV:unique (AA)**	GO:0070654	sensory epithelium regeneration	2.00E-04
**SMV:unique (AB)**	GO:0060346	bone trabecula formation	3.30E-04
**SMV:unique (AB)**	GO:0060037	**pharyngeal system development**	1.10E-08
**SMV:unique (AB)**	GO:0060023	**soft palate development**	3.30E-04
**SMV:unique (AB)**	GO:0060017	parathyroid gland development	3.30E-04
**SMV:unique (AB)**	GO:0072358	cardiovascular system development	4.00E-04
**SMV:unique (AB)**	GO:0043010	camera-type eye development	6.80E-04
**SMV:unique (AB)**	GO:0048639	positive regulation of developmental growth	9.80E-04
**SMV:unique (AB)**	GO:0035162	embryonic hemopoiesis	6.10E-04
**SMV:unique (AB)**	GO:0061072	iris morphogenesis	9.90E-04
**SMV:unique (AB)**	GO:0014835	myoblast differentiation involved in skeletal muscle regeneration	3.30E-04
**SMV:unique (AB)**	GO:0070654	sensory epithelium regeneration	6.60E-04
**SMV:unique (BA)**	GO:0060037	**pharyngeal system development**	1.10E-08
**SMV:unique (BA)**	GO:0060324	face development	3.10E-04
**SMV:unique (BA)**	GO:0043010	camera-type eye development	4.90E-04
**SMV:unique (BA)**	GO:0048635	negative regulation of muscle organ development	9.20E-04
**SMV:unique (BA)**	GO:0031101	fin regeneration	3.30E-04
**SMV:unique (BB)**	GO:0060037	**pharyngeal system development**	3.10E-08
**SMV:unique (BB)**	GO:0060324	face development	4.50E-04
**SV:unique (BB)**	GO:0031101	fin regeneration	1.50E-04

At this point, working with two different draft genomes without high-quality coordinate alignment, the method of variant matching is approximate – and not automated. When a variant (location) was called in both A-B and B-A comparisons it was reported, but, e.g., an *insertion* call in A without a related *deletion* call in B would be omitted. The following example describes the approach leading to **Tab. 11**.

As example:

TM1_T0000027731-R1 (longest transcript)

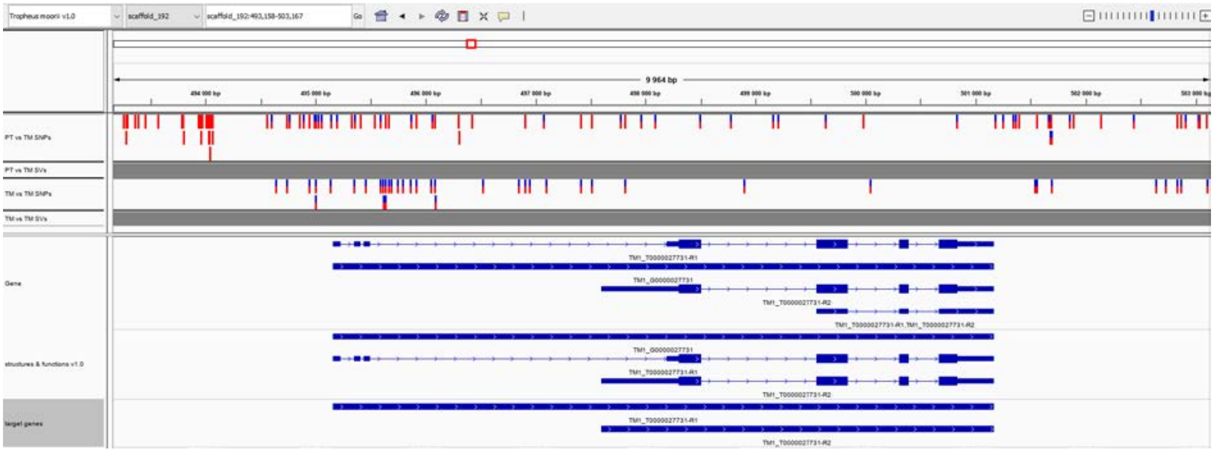

scaffold_192:495158-501167

Type: mRNA

ID: TM1_T0000027731-R1

Parent: TM1_G0000027731

Hit in protein DB (annotation tag: related_db_entry):

PREDICTED: homeobox protein BarH-like 1 [Maylandia zebra] || gi|499018237|ref|XP_004559914.1

Hit in nucleotide DB (annotation tag: nt_db_hit):

PREDICTED: Oreochromis niloticus BARX homeobox 1 (barx1) mRNA || gi|1110941298|ref|XM_019358568.1

PT vs TM SNPs/InDels :

5’UTR, 3’UTR, intron, exon, downstream, upstream, splice

PT vs TM SVs :

inversion

PT1_T0000036546-R2 (longest transcript)

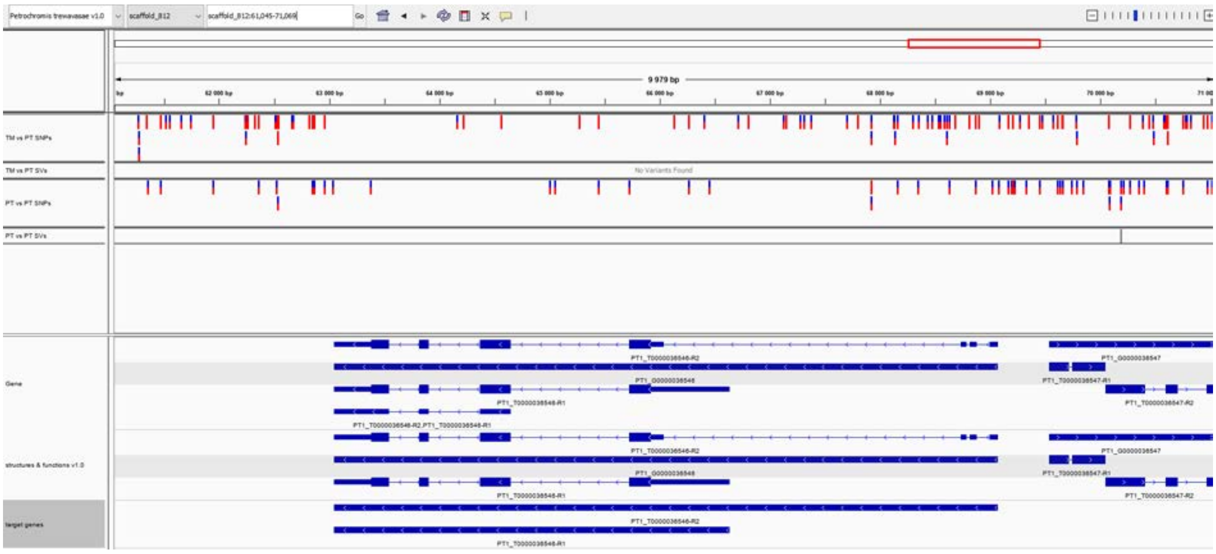

scaffold_812:63045-69069

Type: mRNA

ID: PT1_T0000036546-R2

Parent: PT1_G0000036546

Hit in protein DB (annotation tag: related_db_entry):

PREDICTED: homeobox protein BarH-like 1 [Maylandia zebra] || gi|499018237|ref|XP_004559914.1

Hit in nucleotide DB (annotation tag: nt_db_hit):

PREDICTED: Oreochromis niloticus BARX homeobox 1 (barx1) mRNA || gi|1110941298|ref|XM_019358568.1

TM vs PT SNPs/InDels :

3'UTR, intron, exon, downstream, upstream, splice

TM vs PT SVs :

none

TM1_T0000030271-R1

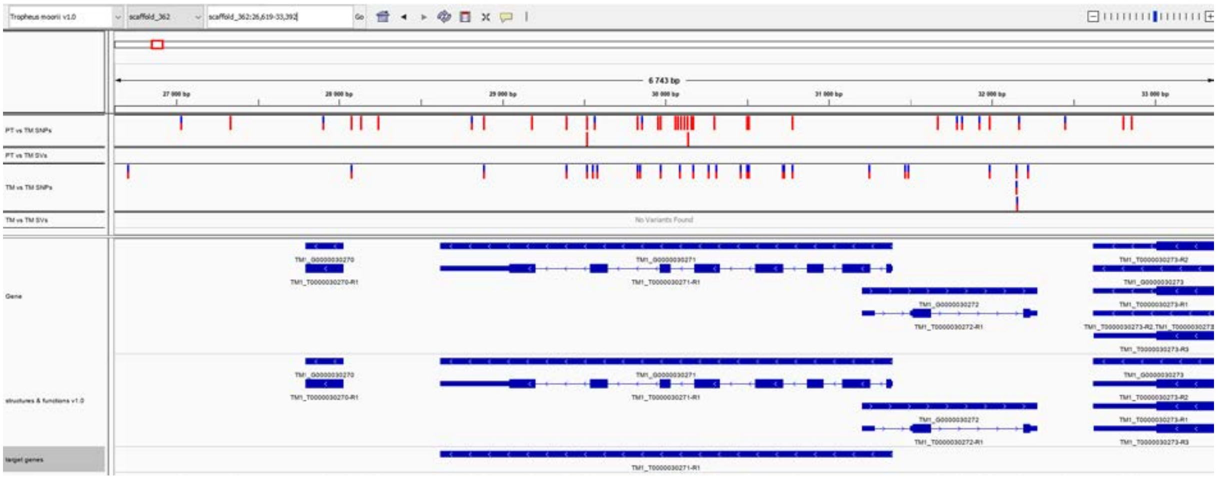

scaffold_362:28619-31392

Type: mRNA

ID: TM1_T0000030271-R1

Parent: TM1_G0000030271

Hit in protein DB (annotation tag: related_db_entry):

PREDICTED: T-box transcription factor TBX10 [Haplochromis burtoni] || gi|554864868|ref|XP_005942488.1

Hit in nucleotide DB (annotation tag: nt_db_hit):

PREDICTED: Haplochromis burtoni T-box 10 (tbx10) mRNA || gi|930760788|ref|XM_005942426.2

PT vs TM SNPs/InDels :

3'UTR, intron, exon, downstream, upstream, splice

PT vs TM SVs :

downstream

PT1_T0000029159-R1

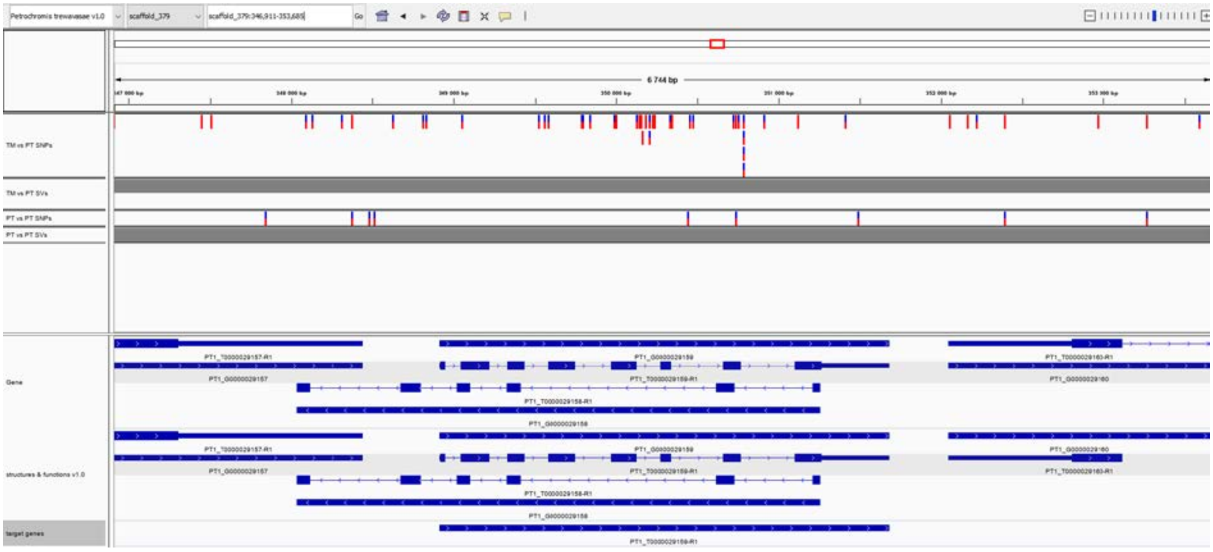

scaffold_379:348911-351685

Type: mRNA

ID: PT1_T0000029159-R1

Parent: PT1_G0000029159

Hit in protein DB (annotation tag: related_db_entry):

PREDICTED: T-box transcription factor TBX10 [Haplochromis burtoni] || gi|554864868|ref|XP_005942488.1

Hit in nucleotide DB (annotation tag: nt_db_hit):

PREDICTED: Haplochromis burtoni T-box 10 (tbx10) mRNA || gi|930760788|ref|XM_005942426.2

TM vs PT SNPs/InDels :

3'UTR, intron, exon, downstream, upstream, splice

TM vs PT SVs :

downstream

Table S19 Variant location overview per gene and transcript. At this point, working with two different draft genomes without high-quality coordinate alignment, the two-way variant matching is approximate. When a variant location was called in both A-B and B-A comparisons it was added in this listing, but, e.g., an *insertion* call in A without a related *deletion* call in B would be omitted. For instance, the inversion called in *barx1* for TM (mapping of PT against TM) but not in PT (mapping of TM against PT) was not included in the final **Tab. 11** (see this example in **Tab. S20**). Moreover, locations regarding individual transcripts were merged at this step to compact the final table.

Gene	ID	Location	SMV	SV	ID	Location	SMV	SV
**PREDICTED: BARX homeobox 1 (barx1)**	**PT1_T0000036546-R1**	scaffold_812: 63045-66634	5'UTR, intron, exon, downstream, upstream	**none**	**TM1_T0000027731-R1**	scaffold_192: 495158-501167	5'UTR, 3'UTR, intron, exon, downstream, upstream, splice	**inversion**
	**PT1_T0000036546-R2**	scaffold_812: 63045-69069	3'UTR, intron, exon, downstream, upstream, splice	**none**	**TM1_T0000027731-R2**	scaffold_192: 497594-501167	5'UTR, 3'UTR, intron, exon, downstream, upstream	**inversion**
**PREDICTED: T-box 10 (tbx10)**	**PT1_T0000029159-R1**	scaffold_379: 348911-351685	3'UTR, intron, exon, downstream, upstream, splice	down-stream	**TM1_T0000030271-R1**	scaffold_362: 28619-31392	3'UTR, intron, exon, downstream, upstream, splice	down-stream
…	…	…	…	…	…	…	…	…

Table S20 Example of Table 11. This table shows a subset of Tab. 11 which highlights the examples shown above.

GENE	DESCRIPTION	VARIANT TYPE	VARIANT LOCATION
**PREDICTED: BARX HOMEOBOX 1 (BARX1)**	*BARX1* represses joints and promotes cartilage formation in the craniofacial skeleton in zebrafish.	SMV<br><br>SV	5' UTR, 3' UTR, intron, exon, downstream, upstream, splice<br><br>-
**PREDICTED: T-BOX 10 (TBX10) ****	Mutations in the *Tbx10* gene in mice and humans are thought to be a cause of isolated cleft lip with or without cleft palate. T-box genes make major contributions to craniofacial development (***Tbx1***, ***Tbx10***, *Tbx15*, *Tbx22*) and to development of the brain (*Tbr1*, *Eomes*), mammary gland (*Tbx2*, *Tbx3*), pituitary gland (*Tbx3*, *Tbx19*), thymus (*Tbx1*), liver (*Tbx3*), lung (*Tbx2*, *Tbx4*, *Tbx5*), pigmentation (*Tbx15*) and the immune system (*Tbx21*), among others.	SMV<br><br>SV	3' UTR, intron, exon, downstream, upstream, splice<br><br>downstream
**…**	…	…	…

Table S21 Additional genes of interest. This table shows genes which are to be analyzed in more detail; it also contains some of the genes listed in Tab. 11. These genes are related to the development of the viscerocranium, the neurocranium and the pharyngeal system; however, there are other GO terms of interest which are consistently enriched over different analysis approaches such as BMP signaling (i.e., bmp family, bmpr family, dlx family, etc.), for instance.

Gene symbol	alt. gene symbol	full name
**aldh1a2**	-	aldehyde dehydrogenase 1 family member A2
**auts2**	-	activator of transcription and developmental regulator AUTS2
**barx1**	-	BARX homeobox 1
**dlx2**	-	distal-less homeobox 2
**edn1**	-	endothelin 1
**faf1**	-	Fas associated factor 1
**fgf8**	-	fibroblast growth factor 8
**foxe1**	-	forkhead box E1
**fras1**	-	Fraser extracellular matrix complex subunit 1
**furin**	-	furin, paired basic amino acid cleaving enzyme
**hand2**	-	heart and neural crest derivatives expressed 2
**kat6a**	-	lysine acetyltransferase 6A
**kctd15**	-	potassium channel tetramerization domain containing 15
**loxl3**	-	lysyl oxidase like 3
**med12**	-	mediator complex subunit 12
**mkx**	-	mohawk homeobox
**nkx3-2**	-	NK3 homeobox 2
**pitx2**	-	paired like homeodomain 2
**plcb3**	-	phospholipase C beta 3
**prdm1**	-	PR/SET domain 1
**rere**	-	arginine-glutamic acid dipeptide repeats
**sec24d**	-	SEC24 homolog D, COPII coat complex component
**shh**	-	sonic hedgehog signaling molecule
**smo**	-	smoothened, frizzled class receptor
**sparc**	-	secreted protein acidic and cysteine rich
**tbx1**	-	T-box transcription factor 1
**tbx10**	-	T-box transcription factor 10
**vgll2**	-	vestigial like family member 2
**wnt9a**	-	Wnt family member 9A
**LOC100705375**	-	GTPase IMAP family member 8-like
**LOC101464109**	sec23a	Sec23 homolog A, coat complex II component
**LOC101466658, LOC102294447**	sec23b	Sec23 homolog B, coat complex II component
**LOC101473565, LOC101474437**	-	placenta-specific gene 8 protein
**LOC101479275, LOC102198405, LOC102199991, LOC102297486, LOC102298608, LOC102299558, LOC102790017, LOC109194420**	-	cornifelin homolog B-like
**LOC102079862, LOC102291746, LOC102310364**	-	caspase-1-like
**LOC102193779**	-	retinoic acid receptor gamma-A-like
**LOC102197772**	alcamb	activated leukocyte cell adhesion molecule b
**LOC102198130, LOC102302319, LOC102798211, LOC102798500,**	-	placenta-specific gene 8 protein-like

Gene symbol	alt. gene symbol	full name
**LOC102798563, LOC102798796, LOC106456499**		
**LOC102206642**	-	uncharacterized LOC102206642; ncRNA
**LOC102209904, LOC102302976, LOC102798646**	igfbp3	insulin-like growth factor binding protein 3
**LOC102210295, LOC102300480, LOC102301658**	-	protein NLRC3-like
**LOC102302136**	-	NACHT, LRR and PYD domains-containing protein 12-like
**LOC102310802**	furina	furin (paired basic amino acid cleaving enzyme) a
**LOC102311194**	kat6a	K(lysine) acetyltransferase 6A
**LOC102777676**	-	furin-1-like
**LOC102779520**	loxl3b	lysyl oxidase-like 3b
**LOC102779574**	pitx2	paired-like homeodomain 2
**LOC102780724**	smo	smoothened, frizzled class receptor
**LOC102786427**	wnt9a	wingless-type MMTV integration site family, member 9A
**LOC102786604, LOC106633073**	fgf20b	fibroblast growth factor 20b
**LOC102788852**	aldh1a2	aldehyde dehydrogenase 1 family, member A2
**LOC102794062**	fras1	Fraser extracellular matrix complex subunit 1
**LOC102796146**	-	retinoic acid receptor gamma-A-like
**LOC102799285, LOC109140771**	rerea	arginine-glutamic acid dipeptide (RE) repeats a
**LOC104950366, LOC109110828**	-	arginine-glutamic acid dipeptide repeats protein-like
**LOC106096905**		protein NLRC3
**LOC108873231**		uncharacterized LOC108873231; protein coding
**LOC109074259**	-	huntingtin-like

Table S22 Parameter settings. This table shows the most important tools used in the project and the associated non-standard parameter settings.

Tool	Task	Parameters (non-listed are set to default)	Comment
**Nextclip**	read filtering	-d -e -m 25 -t 19 -x '34,18' -y '32,17'	Nextera, Illumina MP
**Cutadapt**	read filtering	-e 0.1 -n 3 -O 5	Nextera, Illumina MP
		-e 0.15 -n 3 -O 3	Illumina PE stringent
		-e 0.1 -n 1 -O 5	Illumina PE relaxed
		-e 0.2 -n 3 -O 5	454 stringent
		-e 0.1 -n 2 -O 7	454 relaxed
		-e 0.15 -n 3 -O 3	Illumina RNA-seq stringent
		-e 0.1 -n 1 -O 5	Illumina RNA-seq relaxed
**Reaper**	read filtering	-geom no-bc --clean-length 0 --bcq-late -dust-suffix-late 20 -swp 4/1/3 -nnn-check 5/10 -sc-max 0.25 -polya 3 -dust-suffix-late 20/ATCG	Illumina PE stringent
		-geom no-bc --clean-length 0 -swp 4/1/3 -nnn-check 5/10 -sc-max 0.25	Illumina PE relaxed
		-geom no-bc --clean-length 0 -swp 4/1/3 -nnn-check 5/10 -sc-max 0.25 -dust-suffix-late 20/ATCG	454 stringent
		-geom no-bc --clean-length 0 -swp 4/1/3 -nnn-check 5/10 -sc-max 0.25	454 relaxed
		-geom no-bc --clean-length 0 --bcq-late -swp 4/1/3 -nnn-check 5/10 -sc-max 0.25 -polya 3 -dust-suffix-late 20/TA	Illumina RNA-seq stringent
		-geom no-bc --clean-length 0 -swp 4/1/3 -nnn-check 5/10 -sc-max 0.25	Illumina RNA-seq relaxed
**CLC qualtiy trim**	read filtering	-c 20 -m 30 -b 0.1 -l 0.5	Nextera, Illumina MP
		-c 20 -m 30 -b 0.1 -l 0.5	Illumina PE stringent
		-c 10 -m 25 -b 0.15 -l 0.4	Illumina PE relaxed
		-c 15 -m 30 -b 0.1 -l 0.5	454 stringent
		-c 5 -m 20 -b 0.15 -l 0.4	454 relaxed
		-c 15 -m 25 -b 0.1 -l 0.5	Illumina RNA-seq stringent
		-c 10 -m 20 -b 0.15 -l 0.3	Illumina RNA-seq relaxed
**sffToCA**	read filtering	-clear 454 -trim chop	454 all
**Proovread**	read error correction	-m sr+utg	PacBio
**Musket**	read error correction	-multik 17 25 -maxerr 4 -maxiter 2 -minmulti 0 -maxtrim 0	Illumina DNA-seq all
**Rcorrector**	read error correction	-k 25	Illumina RNA-seq
**SEECER**	read error correction	-k 19	Illumina RNA-seq
**Bbmerge**	read merging	qtrim=t,qtrim=10,minoverlap=12,minoverlap0=8	Illumina PE overlap libraries
**FastUniq**	deduplication	default	all

Tool	Task	Parameters (non-listed are set to default)	Comment
**BWA**	read mapping	-M (-R)	Illumina all
	read mapping	-x pacbio	PacBio
**Bowtie**	read mapping	default	Illumina PE
**Bowtie2**	read mapping	default	Illumina PE
**BLASR**	read mapping	default	PacBio
**STAR**	read mapping	--twopassMode Basic	Illumina RNA-seq
**SMALT (REAPR)**	read mapping	-k 13 -s 2 -y 0.8	
**Platanus**	assembly	-k 25 -s 10 -n 0 -c 2 -a 10 -u 0.1 -d 0.5 -t 32 -m 400	genome; library-related parameters not listed
	scaffolding	-s 32 -v 32 -l 3 -u 0.2 -t 32	
	gap closing	-s 32 -k 32 -d 5000 -vo 32 -vd 32 -eo 1 -ed 0.05 -ro 0.66 -rs 0.9 -t 20	
**MaSuRCA**	assembly | scaffolding | gap closing	GRAPH_KMER_SIZE = auto, USE_LINKING_MATES = 1, LIMIT_JUMP_COVERAGE = 300, CA_PARAMETERS = cgwErrorRate=0.15 ovlMemory=4GB, KMER_COUNT_THRESHOLD = 1, NUM_THREADS = 32, JF_SIZE = 1000000000, DO_HOMOPOLYMER_TRIM = 0	genome; library-related parameters not listed
**Redundans**	polishing (reduction) | scaffolding | gap closing	-t 32 --identity 0.90 --overlap 0.90 --minLength 200 -j 5 -l 0.9 -q 10 --iters 5 --sspacebin SSPACE_Standard_v3.0.pl	genome; library-related parameters not listed
**Celera Assembler**	assembly | scaffolding | gap closing	standard spec.file	genome; library-related parameters not listed
**PBJelly**	assembly | scaffolding | gap closing	default	genome; library-related parameters not listed
**Metassembler**	assembly | scaffolding | gap closing	mateAn_n=10, asseMerge_c=15, asseMerge_i=10, asseMerge_L=10, meta2fasta_keepUnaligned=3, meta2fasta_keepDF=3	genome; library-related parameters not listed
**GMcloser**	scaffolding | gap closing	--long_read --iterate 3 --blast --hetero	PacBio and Illumina PE
**Sealer**	gap closing	-b100G -B 2000 -P10 -k90 -k80 -k70 -k60 -k50 -k40	Illumina PE
**Trinity**	assembly	(--trimmomatic) (--genome_guided_max_intron 500000)	transcriptome
**PASA**	assembly	-C -R --ALIGNERS blat,gmap --transcribed_is_aligned_orient -T --cufflinks --MAX_INTRON_LENGTH 500000 --TRANSDECODER	transcriptome
**Stringtie**	assembly	default	transcriptome
**Cufflinks**	assembly	default	transcriptome
**QUAST**	quality evaluation	--scaffolds --gene-finding --eukaryote	
**REAPR**	quality evaluation	default	
**CEGMA**	quality evaluation	--vrt	
**BUSCO**	quality evaluation	-c 20 -e 0.001 -m geno (-m tran) -sp zebrafish	
**DOGMA**	quality evaluation	proteome -a pfamscan.out -r eukaryotes -c 2 -s 3 -cov 0.5	
**Infernal (cmscan)**	annotation	--rfam	small ncRNAs (rRNAs, microRNAs, etc.); using Rfam v13.0
**FEELnc**	annotation	--mode=shuffle	lncRNAs
**getorf**	annotation	-table 1 -find 1 -minsize 30 -maxsize 10000000	
**cpgplot**	annotation	-window 100 -minlen 200 -minoe 0.6 -minpc 50.	
**RepeatMasker**	annotation	-e ncbi -lib general_and_specific_repeat_library.fa -cutoff 225 -a -inv -poly -lcambig	species-specific library generated with RepeatModeler (default)
**PfamScan**	annotation	-clan_overlap -align -as	using Pfam v32.0
**BRAKER1**	annotation	default	
**MAKER3**	annotation	pcov_blastn=0.8, pid_blastn=0.85, eval_blastn=1e-10, bit_blastn=40, pcov_blastx=0.5, pid_blastx=0.4, eval_blastx=1e-06, bit_blastx=30, pcov_tblastx=0.8, pid_tblastx=0.85, eval_tblastx=1e-10, bit_tblastx=40, pcov_rm_blastx=0.5, pid_rm_blastx=0.4, eval_rm_blastx=1e-06, bit_rm_blastx=30, ep_score_limit=20, en_score_limit=20, evmtrans=10, evmtrans:blastn=1, evmtrans:est2genome=10, evmtrans:tblastx=1, evmtrans:cdna2genome=7, evmprot=10, evmprot:blastx=2, evmprot:protein2genome=10, evmab=10, evmab:snap=7, evmab:augustus=10, evmab:fgenesh=10,	

Tool	Task	Parameters (non-listed are set to default)	Comment
		evmab:genemark=7, est_pass=1, altest_pass=1, protein_pass=1, rm_pass=1, model_pass=1, pred_pass=1, other_pass=1,model_org=all, rmlib=repeatmodeler.lib.fa, repeat_protein=te_proteins.fa, softmask=1, snaphmm=maker3_snap.hmm, gmhmm=braker_gmhmm.mod, augustus_species=pt/tm, pred_gff= ..., run_evm=1, trna=1, pred_flank=200, pred_stats=1, AED_threshold=1, alt_splice=1, keep_preds=0.1, split_hit=500000, single_exon=1, correct_est_fusion=1	
**Funannotate**	annotation	--max_intronlen 500000 --keep_no_stops	
**BLASTP**	annotation	-evalue 1e-3 -word_size 3	coverage and identity thresholds in R post-processing
**BLASTN**	annotation	-task megablast -evalue 1e-3 -word_size 28	coverage and identity thresholds in R post-processing
**BLASTN**	annotation	-task dc-megablast -evalue 1e-3 -word_size 11	coverage and identity thresholds in R post-processing
**BLASTN**	annotation	-perc_identity 90 -qcov_hsp_perc 90	
**eggNOG-Mapper**	annotation	--database fiNOG --dbtype hmmdb --qtype seq --hmm_maxhits 1 --hmm_evalue 0.001 --hmm_score 20 --hmm_maxseqlen 50000 --seed_ortholog_evalue 0.001 --seed_ortholog_score 60 -m hmmer	
**InterProScan 5**	annotation	-dp -goterms -iprlookup -pa -t p	
**GATK**	variant calling	default	best practices implementation
**DELLY**	variant calling	-q 20 (-s 10)	
**SNPeff**	variant effect calling	default	
**Whippet**	alternative splicing calling	(--bam-both-novel) (--bam-min-reads 2)	

Table S23 Overview on sequencing data. Unused libraries are marked in red.

species	origin	technology	version	paired	insert size [mean|sd|median|5mad]	read length [mean|sd|median|5mad]			# of bases			avg. base coverage		
						raw	std	lib	raw	std	lib	raw	std	lib
TM	genomic	Illumina HiSe	PE 2x101	y	269|80|308|70	101|0|101|0	89|15|96|37	94|13|101|0	4 241 839 006	3 123 427 225	3 755 608 468	4.713	3.470	4.173
TM	genomic	Illumina HiSe	PE 2x101	y	269|80|308|70	101|0|101|0	86|17|93|59	92|13|99|15	4 241 839 006	2 996 515 405	3 685 339 939	4.713	3.329	4.095
TM	genomic	Illumina HiSe	PE 2x101	y	270|80|308|70	101|0|101|0	90|14|96|37	96|11|101|0	4 261 760 953	3 326 662 117	3 919 545 967	4.735	3.696	4.355
TM	genomic	Illumina HiSe	PE 2x101	y	270|80|308|70	101|0|101|0	89|15|95|44	94|12|101|0	4 261 760 953	3 280 906 146	3 866 813 581	4.735	3.645	4.296
									**17 007 199 918**	**12 727 510 893**	**15 227 307 955**	**18.897**	**14.142**	**16.919**
TM	genomic	Illumina HiSe	PE 2x100	y	262|39|264|75	100|0|100|0	96|7|98|15	99|5|100|0	4 816 560 800	4 195 977 010	4 581 378 698	5.352	4.662	5.090
TM	genomic	Illumina HiSe	PE 2x100	y	262|39|264|75	100|0|100|0	96|8|98|15	98|6|100|0	4 816 560 800	4 188 982 633	4 565 087 587	5.352	4.654	5.072
									**9 633 121 600**	**8 384 959 643**	**9 146 466 285**	**10.703**	**9.317**	**10.163**
TM	genomic	Illumina HiSe	PE 2x101	y	386|179|456|540	101|0|101|0	87|16|94|52	92|13|98|22	3 933 549 737	2 685 241 766	3 331 958 010	4.371	2.984	3.702
TM	genomic	Illumina HiSe	PE 2x101	y	386|179|456|540	101|0|101|0	82|18|86|96	89|15|96|37	3 933 549 737	2 514 225 496	3 225 995 889	4.371	2.794	3.584
TM	genomic	Illumina HiSe	PE 2x101	y	396|178|496|355	101|0|101|0	91|13|96|37	96|10|101|0	3 846 228 773	2 997 204 212	3 531 902 547	4.274	3.330	3.924
TM	genomic	Illumina HiSe	PE 2x101	y	396|178|496|355	101|0|101|0	87|16|94|52	93|12|98|22	3 846 228 773	2 860 298 792	3 419 154 931	4.274	3.178	3.799
									**15 559 557 020**	**11 056 970 266**	**13 509 011 377**	**17.288**	**12.286**	**15.010**
TM	genomic	Illumina HiSe	MP 2x50	y	9182|3986|10398|7275	50|0|50|0	50|0|50|0	50|0|50|0	178 283 150	151 332 200	151 332 200	0.198	0.168	0.168
TM	genomic	Illumina HiSe	MP 2x50	y	9182|3986|10398|7275	50|0|50|0	50|0|50|0	50|0|50|0	178 283 150	151 332 200	151 332 200	0.198	0.168	0.168
									**356 566 300**	**302 664 400**	**302 664 400**	**0.396**	**0.336**	**0.336**
TM	genomic	Illumina MiSe	PE 2x250	y	305|73|329|145	212|38|242|44	173|25|176|111	212|38|242|44	987 872 669	326 085 670	987 781 616	1.098	0.362	1.098
TM	genomic	Illumina MiSe	PE 2x250	y	305|73|329|145	146|32|168|30	115|17|115|82	146|32|168|30	678 937 930	216 244 647	678 821 016	0.754	0.240	0.754
TM	genomic	Illumina MiSe	PE 2x250	n	-|-|-|-	252|92|234|652	240|85|222|615	246|85|234|652	1 332 238 817	1 238 310 834	1 304 004 881	1.480	1.376	1.449
TM	genomic	Illumina MiSe	PE 2x250	y	301|84|327|190	217|37|244|30	171|25|172|111	217|37|244|30	1 220 533 027	524 307 719	1 220 473 638	1.356	0.583	1.356
TM	genomic	Illumina MiSe	PE 2x250	y	301|84|327|190	146|29|162|15	113|15|115|67	146|29|162|15	819 579 084	345 833 132	819 497 488	0.911	0.384	0.911
TM	genomic	Illumina MiSe	PE 2x250	n	-|-|-|-	259|95|269|630	241|86|225|638	252|87|269|534	934 394 533	833 035 812	909 138 859	1.038	0.926	1.010
									**5 973 556 060**	**3 483 817 814**	**5 919 717 498**	**6.637**	**3.871**	**6.577**
									**2 266 633 350**	**2 071 346 646**	**2 213 143 740**	**2.518**	**2.301**	**2.459**
TM	genomic	Illumina HiSe	Nextera MP 2x150	y	2590|476|2568|1295	150|0|150|0	93|38|106|185	93|38|106|185	4 730 759 550	1 823 563 488	1 823 563 488	5.256	2.026	2.026
TM	genomic	Illumina HiSe	Nextera MP 2x150	y	2590|476|2568|1295	150|0|150|0	94|37|108|170	94|37|108|170	4 730 759 550	1 841 851 223	1 841 851 223	5.256	2.047	2.047
									**9 461 519 100**	**3 665 414 711**	**3 665 414 711**	**10.513**	**4.073**	**4.073**
TM	genomic	Illumina HiSe	Nextera MP 2x150	y	2589|474|2568|1295	150|0|150|0	93|38|106|185	93|38|106|185	4 639 140 600	1 810 502 911	1 810 502 911	5.155	2.012	2.012
TM	genomic	Illumina HiSe	Nextera MP 2x150	y	2589|474|2568|1295	150|0|150|0	94|38|108|170	94|38|108|170	4 639 140 600	1 824 975 300	1 824 975 300	5.155	2.028	2.028
									**9 278 281 200**	**3 635 478 211**	**3 635 478 211**	**10.309**	**4.039**	**4.039**
TM	genomic	Illumina HiSe	Nextera MP 2x150	y	2659|494|2637|1420	150|0|150|0	94|37|107|178	94|37|107|178	5 574 600 900	2 158 299 497	2 158 299 497	6.194	2.398	2.398
TM	genomic	Illumina HiSe	Nextera MP 2x150	y	2659|494|2637|1420	150|0|150|0	95|37|109|163	95|37|109|163	5 574 600 900	2 179 956 271	2 179 956 271	6.194	2.422	2.422
									**11 149 201 800**	**4 338 255 768**	**4 338 255 768**	**12.388**	**4.820**	**4.820**
TM	genomic	Illumina HiSe	Nextera MP 2x150	y	2659|499|2636|1415	150|0|150|0	94|37|107|178	94|37|107|178	5 490 599 850	2 152 519 311	2 152 519 311	6.101	2.392	2.392
TM	genomic	Illumina HiSe	Nextera MP 2x150	y	2659|499|2636|1415	150|0|150|0	95|37|109|163	95|37|109|163	5 490 599 850	2 170 534 778	2 170 534 778	6.101	2.412	2.412
									**10 981 199 700**	**4 323 054 089**	**4 323 054 089**	**12.201**	**4.803**	**4.803**

Table S23 cont. Overview on sequencing data. Unused libraries are marked in red.

species	origin	technology	version	paired	insert size [mean|sd|median|5mad]	read length [mean|sd|median|5mad]			# of bases			avg. base coverage		
						raw	std	lib	raw	std	lib	raw	std	lib
TM	genomic	Illumina HiSe	Nextera MP 2x150	y	5539|1367|5691|3115	150|0|150|0	93|37|104|200	93|37|104|200	6 327 439 500	2 478 812 485	2 478 812 485	7.030	2.754	2.754
TM	genomic	Illumina HiSe	Nextera MP 2x150	y	5539|1367|5691|3115	150|0|150|0	93|37|106|185	93|37|106|185	6 327 439 500	2 496 871 827	2 496 871 827	7.030	2.774	2.774
									**12 654 879 000**	**4 975 684 312**	**4 975 684 312**	**14.061**	**5.529**	**5.529**
TM	genomic	Illumina HiSe	Nextera MP 2x150	y	5538|1362|5691|3105	150|0|150|0	93|37|105|193	93|37|105|193	6 225 111 600	2 463 445 618	2 463 445 618	6.917	2.737	2.737
TM	genomic	Illumina HiSe	Nextera MP 2x150	y	5538|1362|5691|3105	150|0|150|0	93|38|106|185	93|38|106|185	6 225 111 600	2 477 581 049	2 477 581 049	6.917	2.753	2.753
									**12 450 223 200**	**4 941 026 667**	**4 941 026 667**	**13.834**	**5.490**	**5.490**
TM	genomic	454 Life Scien	GS FLX	y	7732|1951|7706|4420	159|98|148|571	159|87|148|519	158|92|148|549	27 195 633	23 260 990	25 854 309	0.030	0.026	0.029
TM	genomic	454 Life Scien	GS FLX	y	7732|1951|7706|4420	169|98|161|578	169|88|162|526	168|93|162|556	28 791 169	24 807 846	27 454 365	0.032	0.028	0.031
TM	genomic	454 Life Scien	GS FLX	y	7722|1973|7704|4490	164|100|151|578	162|89|151|526	162|94|151|556	21 956 501	18 735 540	20 769 616	0.024	0.021	0.023
TM	genomic	454 Life Scien	GS FLX	y	7722|1973|7704|4490	173|102|165|600	174|91|167|549	173|95|166|578	23 284 733	20 127 908	22 140 548	0.026	0.022	0.025
TM	genomic	454 Life Scien	GS FLX	y	7758|1930|7742|4480	155|96|143|549	154|85|143|489	154|90|143|526	25 998 832	21 998 297	24 601 998	0.029	0.024	0.027
TM	genomic	454 Life Scien	GS FLX	y	7758|1930|7742|4480	166|98|158|571	167|87|159|512	166|92|159|549	27 856 420	23 884 550	26 492 803	0.031	0.027	0.029
TM	genomic	454 Life Scien	GS FLX	y	7735|1998|7708|4530	167|103|155|600	166|91|155|541	166|96|156|578	22 331 642	19 136 314	21 181 510	0.025	0.021	0.024
TM	genomic	454 Life Scien	GS FLX	y	7735|1998|7708|4530	176|103|168|615	176|91|169|556	174|96|168|593	23 428 926	20 263 430	22 269 006	0.026	0.023	0.025
									**200 843 856**	**172 214 875**	**190 764 155**	**0.223**	**0.191**	**0.212**
TM	genomic	454 Life Scien	GS FLX	y	7203|6318|4316|19235	171|104|159|615	172|92|161|519	171|96|161|586	3 250 436	2 756 141	3 071 013	0.004	0.003	0.003
TM	genomic	454 Life Scien	GS FLX	y	7203|6318|4316|19235	174|104|163|593	172|90|166|534	171|95|164|571	3 290 736	2 762 638	3 071 105	0.004	0.003	0.003
TM	genomic	454 Life Scien	GS FLX	y	7194|6334|4914|22370	160|99|151|578	162|86|151|512	161|92|152|549	2 939 922	2 481 654	2 782 514	0.003	0.003	0.003
TM	genomic	454 Life Scien	GS FLX	y	7194|6334|4914|22370	167|100|158|571	166|87|160|504	165|93|158|541	3 064 660	2 543 996	2 859 901	0.003	0.003	0.003
									**12 545 754**	**10 544 429**	**11 784 533**	**0.014**	**0.012**	**0.013**
TM	genomic	454 Life Scien	GS FLX	n	-|-|-|-	322|118|344|549	265|92|308|245	265|93|308|245	82 827 451	66 209 305	66 852 919	0.092	0.074	0.074
TM	genomic	454 Life Scien	GS FLX	n	-|-|-|-	307|110|325|534	260|90|293|356	259|91|292|363	108 469 881	89 120 874	89 970 982	0.121	0.099	0.100
TM	genomic	454 Life Scien	GS FLX	n	-|-|-|-	325|124|353|563	266|96|316|185	264|97|315|193	81 018 916	64 053 766	64 710 222	0.090	0.071	0.072
TM	genomic	454 Life Scien	GS FLX	n	-|-|-|-	314|113|336|526	263|92|303|282	262|93|302|289	106 282 173	86 405 654	87 290 154	0.118	0.096	0.097
TM	genomic	454 Life Scien	GS FLX	n	-|-|-|-	344|122|372|534	287|83|341|0	286|84|341|0	186 900 129	150 365 798	151 884 036	0.208	0.167	0.169
TM	genomic	454 Life Scien	GS FLX	n	-|-|-|-	323|118|349|534	277|86|322|141	277|87|322|141	182 241 492	150 749 403	152 283 183	0.202	0.167	0.169
TM	genomic	454 Life Scien	GS FLX	n	-|-|-|-	347|134|386|578	287|92|341|0	288|90|341|0	211 278 155	173 715 264	175 537 936	0.235	0.193	0.195
TM	genomic	454 Life Scien	GS FLX	n	-|-|-|-	336|134|374|600	282|94|341|0	283|93|341|0	193 113 023	161 157 830	162 903 041	0.215	0.179	0.181
									**1 152 131 220**	**941 777 894**	**951 432 473**	**1.280**	**1.046**	**1.057**
TM	transcriptomi	Illumina HiSe	PE 2x100	y	423|697|256|325	100|0|100|0	96|10|100|0	100|2|100|0	8 585 023 000	7 439 124 585	8 563 995 474			
TM	transcriptomi	Illumina HiSe	PE 2x100	y	423|697|256|325	100|0|100|0	95|11|100|0	100|2|100|0	8 585 023 000	7 349 595 252	8 562 588 860			
									**17 170 046 000**	**14 788 719 837**	**17 126 584 334**			
TM	transcriptomi	Illumina HiSe	PE 2x100	y	424|693|260|320	100|0|100|0	96|10|100|0	100|2|100|0	8 643 238 800	7 496 505 980	8 627 841 620			
TM	transcriptomi	Illumina HiSe	PE 2x100	y	424|693|260|320	100|0|100|0	95|11|100|0	100|2|100|0	8 643 238 800	7 403 799 661	8 622 317 150			
									**17 286 477 600**	**14 900 305 641**	**17 250 158 770**			

species	origin	technology	version	paired	insert size [mean|sd|median|5mad]	read length [mean|sd|median|5mad]			# of bases			avg. base coverage		
						raw	trimmed	untrimmed	raw	trimmed	untrimmed	raw	trimmed	untrimmed
TM	genomic	Pacbio		n	-|-|-|-	9738|6647|8662|6885	3182|3027|2120|1913	10166|6223|9239|6350	**11 747 148 050**	**8 191 500 541**	**9 661 264 804**	**13.052**	**9.102**	**10.735**

Table S23 cont. Overview on sequencing data. Unused libraries are marked in red.

<table>
<tr><th>species</th><th>origin</th><th>technology</th><th>version</th><th>paired</th><th>insert size [mean|sd|median|5mad]</th><th colspan="3">read length [mean|sd|median|5mad]</th><th colspan="3"># of bases</th><th colspan="3">avg. base coverage</th></tr>
<tr><th></th><th></th><th></th><th></th><th></th><th></th><th>raw</th><th>std</th><th>lib</th><th>raw</th><th>std</th><th>lib</th><th>raw</th><th>std</th><th>lib</th></tr>
<tr><td>PT</td><td>genomic</td><td>Illumina HiSeq 2000</td><td>PE 2x101</td><td>y</td><td>301|56|315|40</td><td>101|0|101|0</td><td>90|15|97|30</td><td>95|11|101|0</td><td>3 825 192 291</td><td>2 693 013 219</td><td>3 322 674 507</td><td>4.250</td><td>2.992</td><td>3.692</td></tr>
<tr><td>PT</td><td>genomic</td><td>Illumina HiSeq 2000</td><td>PE 2x101</td><td>y</td><td>301|56|315|40</td><td>101|0|101|0</td><td>86|16|93|59</td><td>94|12|101|0</td><td>3 825 192 291</td><td>2 584 730 088</td><td>3 263 301 887</td><td>4.250</td><td>2.872</td><td>3.626</td></tr>
<tr><td>PT</td><td>genomic</td><td>Illumina HiSeq 2000</td><td>PE 2x101</td><td>y</td><td>301|55|315|40</td><td>101|0|101|0</td><td>90|14|96|37</td><td>96|10|101|0</td><td>4 057 254 436</td><td>3 079 884 325</td><td>3 752 079 147</td><td>4.508</td><td>3.422</td><td>4.169</td></tr>
<tr><td>PT</td><td>genomic</td><td>Illumina HiSeq 2000</td><td>PE 2x101</td><td>y</td><td>301|55|315|40</td><td>101|0|101|0</td><td>89|15|95|44</td><td>96|10|101|0</td><td>4 057 254 436</td><td>3 057 483 334</td><td>3 724 707 007</td><td>4.508</td><td>3.397</td><td>4.139</td></tr>
<tr><td></td><td></td><td></td><td></td><td></td><td></td><td></td><td></td><td></td><td>15 764 893 454</td><td>11 415 110 966</td><td>14 062 762 548</td><td>17.517</td><td>12.683</td><td>15.625</td></tr>
<tr><td>PT</td><td>genomic</td><td>Illumina HiSeq 2500</td><td>PE 2x100</td><td>y</td><td>261|48|263|75</td><td>100|0|100|0</td><td>96|8|98|15</td><td>99|5|100|0</td><td>5 579 691 700</td><td>4 798 906 774</td><td>5 278 407 168</td><td>6.200</td><td>5.332</td><td>5.865</td></tr>
<tr><td>PT</td><td>genomic</td><td>Illumina HiSeq 2500</td><td>PE 2x100</td><td>y</td><td>261|48|263|75</td><td>100|0|100|0</td><td>96|8|98|15</td><td>98|6|100|0</td><td>5 579 691 700</td><td>4 817 850 855</td><td>5 266 022 881</td><td>6.200</td><td>5.353</td><td>5.851</td></tr>
<tr><td></td><td></td><td></td><td></td><td></td><td></td><td></td><td></td><td></td><td>11 159 383 400</td><td>9 616 757 629</td><td>10 544 430 049</td><td>12.399</td><td>10.685</td><td>11.716</td></tr>
<tr><td>PT</td><td>genomic</td><td>Illumina HiSeq2000</td><td>PE 2x101</td><td>y</td><td>504|130|557|70</td><td>101|0|101|0</td><td>89|16|96|37</td><td>93|13|101|0</td><td>3 599 896 035</td><td>2 446 762 722</td><td>3 061 794 550</td><td>4.000</td><td>2.719</td><td>3.402</td></tr>
<tr><td>PT</td><td>genomic</td><td>Illumina HiSeq2000</td><td>PE 2x101</td><td>y</td><td>504|130|557|70</td><td>101|0|101|0</td><td>77|18|80|104</td><td>86|16|90|82</td><td>3 599 896 035</td><td>2 118 664 878</td><td>2 823 585 700</td><td>4.000</td><td>2.354</td><td>3.137</td></tr>
<tr><td>PT</td><td>genomic</td><td>Illumina HiSeq2000</td><td>PE 2x101</td><td>y</td><td>505|126|557|70</td><td>101|0|101|0</td><td>90|14|97|30</td><td>96|10|101|0</td><td>3 797 039 854</td><td>2 848 680 626</td><td>3 460 748 391</td><td>4.219</td><td>3.165</td><td>3.845</td></tr>
<tr><td>PT</td><td>genomic</td><td>Illumina HiSeq2000</td><td>PE 2x101</td><td>y</td><td>505|126|557|70</td><td>101|0|101|0</td><td>84|17|90|74</td><td>91|13|98|22</td><td>3 797 039 854</td><td>2 643 531 694</td><td>3 290 235 002</td><td>4.219</td><td>2.937</td><td>3.656</td></tr>
<tr><td>PT</td><td>genomic</td><td>Illumina HiSeq2500</td><td>PE 2x100</td><td>y</td><td>503|129|558|70</td><td>100|0|100|0</td><td>95|11|100|0</td><td>98|7|100|0</td><td>12 363 909 300</td><td>10 470 237 450</td><td>11 933 206 067</td><td>13.738</td><td>11.634</td><td>13.259</td></tr>
<tr><td>PT</td><td>genomic</td><td>Illumina HiSeq2500</td><td>PE 2x100</td><td>y</td><td>503|129|558|70</td><td>100|0|100|0</td><td>94|11|98|15</td><td>97|8|100|0</td><td>12 363 909 300</td><td>10 367 567 933</td><td>11 835 236 423</td><td>13.738</td><td>11.520</td><td>13.150</td></tr>
<tr><td>PT</td><td>genomic</td><td>Illumina HiSeq2500</td><td>PE 2x100</td><td>y</td><td>495|136|556|80</td><td>100|0|100|0</td><td>94|12|98|15</td><td>97|8|100|0</td><td>14 962 889 400</td><td>11 005 412 843</td><td>13 992 497 562</td><td>16.625</td><td>12.228</td><td>15.547</td></tr>
<tr><td>PT</td><td>genomic</td><td>Illumina HiSeq2500</td><td>PE 2x100</td><td>y</td><td>495|136|556|80</td><td>100|0|100|0</td><td>92|13|98|15</td><td>95|11|100|0</td><td>14 962 889 400</td><td>10 785 726 311</td><td>13 735 525 240</td><td>16.625</td><td>11.984</td><td>15.262</td></tr>
<tr><td></td><td></td><td></td><td></td><td></td><td></td><td></td><td></td><td></td><td>69 447 469 178</td><td>52 686 584 457</td><td>64 132 828 935</td><td>77.164</td><td>58.541</td><td>71.259</td></tr>
<tr><td>PT</td><td>genomic</td><td>Illumina MiSeq</td><td>PE 2x250</td><td>y</td><td>361|189|406|450</td><td>215|39|230|170</td><td>214|48|239|96</td><td>215|39|230|170</td><td>924 021 802</td><td>230 439 131</td><td>923 864 336</td><td>1.027</td><td>0.256</td><td>1.027</td></tr>
<tr><td>PT</td><td>genomic</td><td>Illumina MiSeq</td><td>PE 2x250</td><td>y</td><td>361|189|406|450</td><td>143|29|159|15</td><td>132|30|143|126</td><td>143|29|159|15</td><td>616 342 511</td><td>141 796 698</td><td>616 192 880</td><td>0.685</td><td>0.158</td><td>0.685</td></tr>
<tr><td>PT</td><td>genomic</td><td>Illumina MiSeq</td><td>PE 2x250</td><td>n</td><td>-|-|-|-</td><td>222|65|213|304</td><td>217|62|211|304</td><td>221|62|213|304</td><td>3 018 946 305</td><td>2 917 942 990</td><td>2 998 998 102</td><td>3.354</td><td>3.242</td><td>3.332</td></tr>
<tr><td>PT</td><td>genomic</td><td>Illumina MiSeq</td><td>PE 2x250</td><td>y</td><td>272|190|260|385</td><td>203|41|177|126</td><td>211|46|234|89</td><td>203|40|177|126</td><td>1 482 865 035</td><td>212 128 206</td><td>1 482 654 271</td><td>1.648</td><td>0.236</td><td>1.647</td></tr>
<tr><td>PT</td><td>genomic</td><td>Illumina MiSeq</td><td>PE 2x250</td><td>y</td><td>272|190|260|385</td><td>233|37|247|15</td><td>167|40|168|200</td><td>233|37|247|15</td><td>1 704 048 895</td><td>167 978 771</td><td>1 702 943 641</td><td>1.893</td><td>0.187</td><td>1.892</td></tr>
<tr><td>PT</td><td>genomic</td><td>Illumina MiSeq</td><td>PE 2x250</td><td>n</td><td>-|-|-|-</td><td>292|103|271|571</td><td>258|71|261|393</td><td>266|72|271|519</td><td>2 782 263 272</td><td>2 418 095 266</td><td>2 532 229 569</td><td>3.091</td><td>2.687</td><td>2.814</td></tr>
<tr><td></td><td></td><td></td><td></td><td></td><td></td><td></td><td></td><td></td><td>10 528 487 820</td><td>6 088 381 062</td><td>10 256 882 799</td><td>11.698</td><td>6.765</td><td>11.397</td></tr>
<tr><td></td><td></td><td></td><td></td><td></td><td></td><td></td><td></td><td></td><td>5 801 209 577</td><td>5 336 038 256</td><td>5 531 227 671</td><td>6.446</td><td>5.929</td><td>6.146</td></tr>
<tr><td>PT</td><td>genomic</td><td>Illumina HiSeq 2500</td><td>MP 2x50</td><td>y</td><td>24022|6944|24902|6270</td><td>50|0|50|0</td><td>50|0|50|0</td><td>50|0|50|0</td><td>126 233 500</td><td>121 030 550</td><td>121 030 550</td><td>0.140</td><td>0.134</td><td>0.134</td></tr>
<tr><td>PT</td><td>genomic</td><td>Illumina HiSeq 2500</td><td>MP 2x50</td><td>y</td><td>24022|6944|24902|6270</td><td>50|0|50|0</td><td>50|0|50|0</td><td>50|0|50|0</td><td>126 233 500</td><td>121 030 550</td><td>121 030 550</td><td>0.140</td><td>0.134</td><td>0.134</td></tr>
<tr><td></td><td></td><td></td><td></td><td></td><td></td><td></td><td></td><td></td><td>252 467 000</td><td>242 061 100</td><td>242 061 100</td><td>0.281</td><td>0.269</td><td>0.269</td></tr>
<tr><td>PT</td><td>genomic</td><td>Illumina HiSeq 2500</td><td>Nextera MP 2x150</td><td>y</td><td>2494|1450|3077|4200</td><td>50|0|50|0</td><td>50|0|50|0</td><td>50|0|50|0</td><td>264 339 700</td><td>187 537 850</td><td>187 537 850</td><td>0.294</td><td>0.208</td><td>0.208</td></tr>
<tr><td>PT</td><td>genomic</td><td>Illumina HiSeq 2500</td><td>Nextera MP 2x150</td><td>y</td><td>2494|1450|3077|4200</td><td>50|0|50|0</td><td>50|0|50|0</td><td>50|0|50|0</td><td>264 339 700</td><td>187 537 850</td><td>187 537 850</td><td>0.294</td><td>0.208</td><td>0.208</td></tr>
<tr><td></td><td></td><td></td><td></td><td></td><td></td><td></td><td></td><td></td><td>528 679 400</td><td>375 075 700</td><td>375 075 700</td><td>0.587</td><td>0.417</td><td>0.417</td></tr>
<tr><td>PT</td><td>genomic</td><td>Illumina HiSeq 2500</td><td>Nextera MP 2x150</td><td>y</td><td>4755|1432|4922|3015</td><td>150|0|150|0</td><td>92|38|103|208</td><td>92|38|103|208</td><td>7 378 837 200</td><td>2 788 304 600</td><td>2 788 304 600</td><td>8.199</td><td>3.098</td><td>3.098</td></tr>
<tr><td>PT</td><td>genomic</td><td>Illumina HiSeq 2500</td><td>Nextera MP 2x150</td><td>y</td><td>4755|1432|4922|3015</td><td>150|0|150|0</td><td>93|38|105|193</td><td>93|38|105|193</td><td>7 378 837 200</td><td>2 808 645 777</td><td>2 808 645 777</td><td>8.199</td><td>3.121</td><td>3.121</td></tr>
<tr><td></td><td></td><td></td><td></td><td></td><td></td><td></td><td></td><td></td><td>14 757 674 400</td><td>5 596 950 377</td><td>5 596 950 377</td><td>16.397</td><td>6.219</td><td>6.219</td></tr>
<tr><td>PT</td><td>genomic</td><td>Illumina HiSeq 2500</td><td>Nextera MP 2x150</td><td>y</td><td>4756|1424|4923|3010</td><td>150|0|150|0</td><td>92|38|104|200</td><td>92|38|104|200</td><td>7 232 523 600</td><td>2 763 603 659</td><td>2 763 603 659</td><td>8.036</td><td>3.071</td><td>3.071</td></tr>
<tr><td>PT</td><td>genomic</td><td>Illumina HiSeq 2500</td><td>Nextera MP 2x150</td><td>y</td><td>4756|1424|4923|3010</td><td>150|0|150|0</td><td>93|38|105|193</td><td>93|38|105|193</td><td>7 232 523 600</td><td>2 777 699 147</td><td>2 777 699 147</td><td>8.036</td><td>3.086</td><td>3.086</td></tr>
<tr><td></td><td></td><td></td><td></td><td></td><td></td><td></td><td></td><td></td><td>14 465 047 200</td><td>5 541 302 806</td><td>5 541 302 806</td><td>16.072</td><td>6.157</td><td>6.157</td></tr>
<tr><td>PT</td><td>genomic</td><td>Illumina HiSeq 2500</td><td>Nextera MP 2x150</td><td>y</td><td>2748|566|2738|1000</td><td>150|0|150|0</td><td>92|38|103|208</td><td>92|38|103|208</td><td>12 817 713 000</td><td>4 834 846 811</td><td>4 834 846 811</td><td>14.242</td><td>5.372</td><td>5.372</td></tr>
<tr><td>PT</td><td>genomic</td><td>Illumina HiSeq 2500</td><td>Nextera MP 2x150</td><td>y</td><td>2748|566|2738|1000</td><td>150|0|150|0</td><td>92|38|104|200</td><td>92|38|104|200</td><td>12 817 713 000</td><td>4 836 060 946</td><td>4 836 060 946</td><td>14.242</td><td>5.373</td><td>5.373</td></tr>
<tr><td></td><td></td><td></td><td></td><td></td><td></td><td></td><td></td><td></td><td>25 635 426 000</td><td>9 670 907 757</td><td>9 670 907 757</td><td>28.484</td><td>10.745</td><td>10.745</td></tr>
<tr><td>PT</td><td>genomic</td><td>Illumina HiSeq 2500</td><td>Nextera MP 2x150</td><td>y</td><td>6194|1541|6269|2485</td><td>150|0|150|0</td><td>95|37|111|148</td><td>95|37|111|148</td><td>11 511 042 300</td><td>4 392 439 126</td><td>4 392 439 126</td><td>12.790</td><td>4.880</td><td>4.880</td></tr>
<tr><td>PT</td><td>genomic</td><td>Illumina HiSeq 2500</td><td>Nextera MP 2x150</td><td>y</td><td>6194|1541|6269|2485</td><td>150|0|150|0</td><td>95|37|112|141</td><td>95|37|112|141</td><td>11 511 042 300</td><td>4 399 775 005</td><td>4 399 775 005</td><td>12.790</td><td>4.889</td><td>4.889</td></tr>
<tr><td></td><td></td><td></td><td></td><td></td><td></td><td></td><td></td><td></td><td>23 022 084 600</td><td>8 792 214 131</td><td>8 792 214 131</td><td>25.580</td><td>9.769</td><td>9.769</td></tr>
</table>

Table S23 cont. Overview on sequencing data. Unused libraries are marked in red.

<table>
<tr><th>species</th><th>origin</th><th>technology</th><th>version</th><th>paired</th><th>insert size [mean|sd|median|5mad]</th><th colspan="3">read length [mean|sd|median|5mad]</th><th colspan="3"># of bases</th><th colspan="3">avg. base coverage</th></tr>
<tr><th></th><th></th><th></th><th></th><th></th><th></th><th>raw</th><th>std</th><th>lib</th><th>raw</th><th>std</th><th>lib</th><th>raw</th><th>std</th><th>lib</th></tr>
<tr><td>PT</td><td>genomic</td><td>454 Life Sciences</td><td>GS FLX</td><td>y</td><td>8239|2044|8175|2245</td><td>135|82|123|452</td><td>134|74|122|408</td><td>134|79|123|437</td><td>13 696 810</td><td>11 295 917</td><td>12 929 258</td><td>0.015</td><td>0.013</td><td>0.014</td></tr>
<tr><td>PT</td><td>genomic</td><td>455 Life Sciences</td><td>GS FLX</td><td>y</td><td>8239|2044|8175|2245</td><td>145|86|137|497</td><td>147|77|138|445</td><td>146|82|138|474</td><td>14 761 537</td><td>12 397 325</td><td>14 107 699</td><td>0.016</td><td>0.014</td><td>0.016</td></tr>
<tr><td>PT</td><td>genomic</td><td>456 Life Sciences</td><td>GS FLX</td><td>y</td><td>6536|1862|6987|2815</td><td>157|97|145|556</td><td>157|86|146|504</td><td>156|91|145|541</td><td>27 431 443</td><td>23 196 680</td><td>25 947 218</td><td>0.030</td><td>0.026</td><td>0.029</td></tr>
<tr><td>PT</td><td>genomic</td><td>457 Life Sciences</td><td>GS FLX</td><td>y</td><td>6536|1862|6987|2815</td><td>166|99|156|578</td><td>166|88|157|519</td><td>165|93|157|556</td><td>28 902 906</td><td>24 481 924</td><td>27 460 329</td><td>0.032</td><td>0.027</td><td>0.031</td></tr>
<tr><td>PT</td><td>genomic</td><td>458 Life Sciences</td><td>GS FLX</td><td>y</td><td>6552|1847|6991|2785</td><td>152|94|140|541</td><td>152|84|141|489</td><td>151|89|140|526</td><td>22 202 838</td><td>18 750 514</td><td>21 047 756</td><td>0.025</td><td>0.021</td><td>0.023</td></tr>
<tr><td>PT</td><td>genomic</td><td>459 Life Sciences</td><td>GS FLX</td><td>y</td><td>6552|1847|6991|2785</td><td>161|96|152|563</td><td>162|86|154|512</td><td>161|91|154|549</td><td>23 605 378</td><td>19 962 836</td><td>22 502 332</td><td>0.026</td><td>0.022</td><td>0.025</td></tr>
<tr><td>PT</td><td>genomic</td><td>460 Life Sciences</td><td>GS FLX</td><td>y</td><td>6539|1868|6979|2835</td><td>149|91|138|526</td><td>149|82|139|474</td><td>148|87|138|512</td><td>22 201 268</td><td>18 706 550</td><td>21 057 803</td><td>0.025</td><td>0.021</td><td>0.023</td></tr>
<tr><td>PT</td><td>genomic</td><td>461 Life Sciences</td><td>GS FLX</td><td>y</td><td>6539|1868|6979|2835</td><td>158|93|149|541</td><td>158|83|149|482</td><td>158|88|150|519</td><td>23 534 549</td><td>19 821 412</td><td>22 449 483</td><td>0.026</td><td>0.022</td><td>0.025</td></tr>
<tr><td>PT</td><td>genomic</td><td>462 Life Sciences</td><td>GS FLX</td><td>y</td><td>6570|1862|7023|2770</td><td>136|83|125|474</td><td>136|74|126|423</td><td>136|79|125|452</td><td>22 615 577</td><td>18 854 612</td><td>21 441 877</td><td>0.025</td><td>0.021</td><td>0.024</td></tr>
<tr><td>PT</td><td>genomic</td><td>463 Life Sciences</td><td>GS FLX</td><td>y</td><td>6570|1862|7023|2770</td><td>145|84|138|489</td><td>146|76|138|430</td><td>146|81|139|467</td><td>24 231 365</td><td>20 180 921</td><td>23 079 947</td><td>0.027</td><td>0.022</td><td>0.026</td></tr>
<tr><td>PT</td><td>genomic</td><td>464 Life Sciences</td><td>GS FLX</td><td>y</td><td>8226|2096|8173|2250</td><td>137|84|125|467</td><td>137|75|125|415</td><td>137|80|126|452</td><td>14 554 501</td><td>12 101 902</td><td>13 749 630</td><td>0.016</td><td>0.013</td><td>0.015</td></tr>
<tr><td>PT</td><td>genomic</td><td>465 Life Sciences</td><td>GS FLX</td><td>y</td><td>8226|2096|8173|2250</td><td>150|88|141|512</td><td>151|79|142|460</td><td>151|84|142|489</td><td>15 865 829</td><td>13 344 734</td><td>15 142 053</td><td>0.018</td><td>0.015</td><td>0.017</td></tr>
<tr><td></td><td></td><td></td><td></td><td></td><td></td><td></td><td></td><td></td><td>253 604 001</td><td>213 095 327</td><td>240 915 385</td><td>0.282</td><td>0.237</td><td>0.268</td></tr>
<tr><td>PT</td><td>genomic</td><td>454 Life Sciences</td><td>GS FLX</td><td>y</td><td>6843|5320|3577|11210</td><td>191|94|181|608</td><td>181|85|176|571</td><td>187|88|181|608</td><td>367 816</td><td>328 702</td><td>354 989</td><td>0.000</td><td>0.000</td><td>0.000</td></tr>
<tr><td>PT</td><td>genomic</td><td>455 Life Sciences</td><td>GS FLX</td><td>y</td><td>6843|5320|3577|11210</td><td>170|87|167|541</td><td>178|81|168|526</td><td>172|85|167|534</td><td>327 822</td><td>323 636</td><td>327 005</td><td>0.000</td><td>0.000</td><td>0.000</td></tr>
<tr><td>PT</td><td>genomic</td><td>456 Life Sciences</td><td>GS FLX</td><td>y</td><td>7178|5390|3577|11210</td><td>186|90|176|552</td><td>177|82|176|549</td><td>183|85|176|552</td><td>352 642</td><td>317 475</td><td>339 895</td><td>0.000</td><td>0.000</td><td>0.000</td></tr>
<tr><td>PT</td><td>genomic</td><td>457 Life Sciences</td><td>GS FLX</td><td>y</td><td>7178|5390|3577|11210</td><td>162|85|157|519</td><td>169|81|162|526</td><td>164|84|157|512</td><td>305 976</td><td>302 412</td><td>305 206</td><td>0.000</td><td>0.000</td><td>0.000</td></tr>
<tr><td></td><td></td><td></td><td></td><td></td><td></td><td></td><td></td><td></td><td>1 354 256</td><td>1 272 225</td><td>1 327 095</td><td>0.002</td><td>0.001</td><td>0.001</td></tr>
<tr><td>PT</td><td>genomic</td><td>454 Life Sciences</td><td>GS FLX</td><td>n</td><td>-|-|-|-</td><td>314|114|336|519</td><td>264|91|302|289</td><td>263|93|302|289</td><td>105 608 833</td><td>85 738 311</td><td>86 861 282</td><td>0.117</td><td>0.095</td><td>0.097</td></tr>
<tr><td>PT</td><td>genomic</td><td>455 Life Sciences</td><td>GS FLX</td><td>n</td><td>-|-|-|-</td><td>299|114|321|541</td><td>255|93|288|393</td><td>254|95|287|400</td><td>90 968 228</td><td>74 639 486</td><td>75 610 245</td><td>0.101</td><td>0.083</td><td>0.084</td></tr>
<tr><td>PT</td><td>genomic</td><td>456 Life Sciences</td><td>GS FLX</td><td>n</td><td>-|-|-|-</td><td>300|103|318|467</td><td>260|87|288|393</td><td>259|88|288|393</td><td>94 410 425</td><td>79 265 523</td><td>80 293 825</td><td>0.105</td><td>0.088</td><td>0.089</td></tr>
<tr><td>PT</td><td>genomic</td><td>457 Life Sciences</td><td>GS FLX</td><td>n</td><td>-|-|-|-</td><td>276|92|290|437</td><td>246|84|264|445</td><td>246|84|264|445</td><td>111 484 711</td><td>96 664 313</td><td>97 963 317</td><td>0.124</td><td>0.107</td><td>0.109</td></tr>
<tr><td>PT</td><td>genomic</td><td>458 Life Sciences</td><td>GS FLX</td><td>n</td><td>-|-|-|-</td><td>277|97|291|489</td><td>245|85|264|482</td><td>245|86|264|489</td><td>111 357 923</td><td>95 552 039</td><td>96 810 857</td><td>0.124</td><td>0.106</td><td>0.108</td></tr>
<tr><td>PT</td><td>genomic</td><td>459 Life Sciences</td><td>GS FLX</td><td>n</td><td>-|-|-|-</td><td>268|96|280|497</td><td>239|86|254|489</td><td>238|86|254|489</td><td>106 496 920</td><td>91 683 429</td><td>92 956 103</td><td>0.118</td><td>0.102</td><td>0.103</td></tr>
<tr><td>PT</td><td>genomic</td><td>460 Life Sciences</td><td>GS FLX</td><td>n</td><td>-|-|-|-</td><td>350|113|370|497</td><td>292|78|341|0</td><td>292|77|341|0</td><td>115 538 705</td><td>94 608 132</td><td>95 755 590</td><td>0.128</td><td>0.105</td><td>0.106</td></tr>
<tr><td>PT</td><td>genomic</td><td>461 Life Sciences</td><td>GS FLX</td><td>n</td><td>-|-|-|-</td><td>336|107|355|467</td><td>287|78|330|82</td><td>288|78|332|67</td><td>113 432 394</td><td>95 278 484</td><td>96 415 113</td><td>0.126</td><td>0.106</td><td>0.107</td></tr>
<tr><td>PT</td><td>genomic</td><td>462 Life Sciences</td><td>GS FLX</td><td>n</td><td>-|-|-|-</td><td>343|133|382|578</td><td>286|92|341|0</td><td>287|91|341|0</td><td>223 301 430</td><td>184 552 133</td><td>186 700 709</td><td>0.248</td><td>0.205</td><td>0.207</td></tr>
<tr><td>PT</td><td>genomic</td><td>463 Life Sciences</td><td>GS FLX</td><td>n</td><td>-|-|-|-</td><td>338|123|370|541</td><td>289|87|341|0</td><td>290|85|341|0</td><td>235 540 726</td><td>199 528 557</td><td>201 821 412</td><td>0.262</td><td>0.222</td><td>0.224</td></tr>
<tr><td></td><td></td><td></td><td></td><td></td><td></td><td></td><td></td><td></td><td>1 308 140 295</td><td>1 097 510 407</td><td>1 111 188 453</td><td>1.453</td><td>1.219</td><td>1.235</td></tr>
<tr><td>PT</td><td>transcriptomic</td><td>Illumina HiSeq 2500</td><td>PE 2x100</td><td>y</td><td>473|970|268|290</td><td>100|0|100|0</td><td>96|10|100|0</td><td>100|2|100|0</td><td>11 613 862 800</td><td>10 037 776 507</td><td>11 517 741 977</td><td></td><td></td><td></td></tr>
<tr><td>PT</td><td>transcriptomic</td><td>Illumina HiSeq 2500</td><td>PE 2x100</td><td>y</td><td>473|970|268|290</td><td>100|0|100|0</td><td>94|12|99|7</td><td>100|3|100|0</td><td>11 613 862 800</td><td>9 879 492 321</td><td>11 509 517 371</td><td></td><td></td><td></td></tr>
<tr><td></td><td></td><td></td><td></td><td></td><td></td><td></td><td></td><td></td><td>23 227 725 600</td><td>19 917 268 828</td><td>23 027 259 348</td><td></td><td></td><td></td></tr>
<tr><td>PT</td><td>transcriptomic</td><td>Illumina HiSeq 2500</td><td>PE 2x100</td><td>y</td><td>522|1106|280|305</td><td>100|0|100|0</td><td>96|10|100|0</td><td>100|2|100|0</td><td>11 729 958 200</td><td>9 880 359 444</td><td>11 654 289 630</td><td></td><td></td><td></td></tr>
<tr><td>PT</td><td>transcriptomic</td><td>Illumina HiSeq 2500</td><td>PE 2x100</td><td>y</td><td>522|1106|280|305</td><td>100|0|100|0</td><td>94|12|98|15</td><td>100|2|100|0</td><td>11 729 958 200</td><td>9 675 478 615</td><td>11 643 633 858</td><td></td><td></td><td></td></tr>
<tr><td></td><td></td><td></td><td></td><td></td><td></td><td></td><td></td><td></td><td>23 459 916 400</td><td>19 555 838 059</td><td>23 297 923 488</td><td></td><td></td><td></td></tr>
</table>

<table>
<tr><th>species</th><th>origin</th><th>technology</th><th>version</th><th>paired</th><th>insert size [mean|sd|median|5mad]</th><th colspan="3">read length [mean|sd|median|5mad]</th><th colspan="3"># of bases</th><th colspan="3">avg. base coverage</th></tr>
<tr><th></th><th></th><th></th><th></th><th></th><th></th><th>raw</th><th>trimmed</th><th>untrimmed</th><th>raw</th><th>trimmed</th><th>untrimmed</th><th>raw</th><th>trimmed</th><th>untrimmed</th></tr>
<tr><td>PT</td><td>genomic</td><td>Pacbio</td><td></td><td>n</td><td>-|-|-|-</td><td>8079|6070|6717|5871</td><td>3562|3299|2444|2233</td><td>8108|5948|6792|5809</td><td>12 231 345 577</td><td>9 421 813 094</td><td>10 807 790 909</td><td>13.590</td><td>10.469</td><td>12.009</td></tr>
</table>
